# *gem*-Difluorobisarylic derivatives: design, synthesis and anti-inflammatory effect

**DOI:** 10.1186/s13065-019-0640-5

**Published:** 2019-10-31

**Authors:** Abeer J. Ayoub, Layal Hariss, Nehme El-Hachem, Ghewa A. El-Achkar, Sandra E. Ghayad, Oula K. Dagher, Nada Borghol, René Grée, Bassam Badran, Ali Hachem, Eva Hamade, Aida Habib

**Affiliations:** 1 Department of Biochemistry and Molecular Genetics, Faculty of Medicine, American University of Beirut, Beirut, Lebanon; 2 Laboratory of Cancer Biology and Molecular Immunology, Faculty of Sciences I, Lebanese University, Hadath, Beirut, Lebanon; 3 Laboratory for Medicinal Chemistry and Natural Products, Faculty of Sciences I and PRASE-EDST Lebanese University, Beirut, Lebanon; 4 Integrative Systems Biology, Institut de Recherches Cliniques de Montréal, Montreal, QC Canada; 5 Department of Biology, Faculty of Sciences II, EDST, Lebanese University, Fanar, Lebanon; 6 Université de Rennes, CNRS, ISCR (Institut des Sciences Chimiques de Rennes) UMR 6226, 35000 Rennes, France; 7 Université de Paris, Centre de Recherche sur l’Inflammation (CRI), INSERM, UMR1149, CNRS, ERL 8252, 75018 Paris, France; 8 Present Address: Department of Electrical and Computer Engineering, American University of Beirut, Beirut, Lebanon

**Keywords:** Fluorine, Diaryl ethers, Macrophages, Cyclooxygenase, Inflammation

## Abstract

**Introduction:**

New fluorinated diaryl ethers and bisarylic ketones were designed and evaluated for their anti-inflammatory effects in primary macrophages.

**Methods:**

The synthesis of the designed molecules started from easily accessible and versatile *gem*-difluoro propargylic derivatives. The desired aromatic systems were obtained using Diels–Alder/aromatization sequences and this was followed by Pd-catalyzed coupling reactions and, when required, final functionalization steps. Both direct inhibitory effects on cyclooxygenase-1 or -2 activities, protein expression of cyclooxygenase-2 and nitric oxide synthase-II and the production of prostaglandin E_2_, the pro-inflammatory nitric oxide and interleukin-6 were evaluated in primary murine bone marrow-derived macrophages in response to lipopolysaccharide. Docking of the designed molecules in cyclooxygenase-1 or -2 was performed.

**Results:**

Only fluorinated compounds exerted anti-inflammatory activities by lowering the secretion of interleukin-6, nitric oxide, and prostaglandin E_2_, and decreasing the protein expression of inducible nitric oxide synthase and cyclooxygenase-2 in mouse primary macrophages exposed to lipopolysaccharide, as well as cyclooxygenase activity for some inhibitors with different efficiencies depending on the R-groups. Docking observation suggested an inhibitory role of cyclooxygenase-1 or -2 for compounds **A3**, **A4** and **A5** in addition to their capacity to inhibit nitrite, interleukin-6, and nitric oxide synthase-II and cyclooxygenase-2 expression.

**Conclusion:**

The new fluorinated diaryl ethers and bisarylic ketones have anti-inflammatory effects in macrophages. These fluorinated compounds have improved potential anti-inflammatory properties due to the fluorine residues in the bioactive molecules.

## Introduction

Diaryl ethers are key scaffolds present in many natural or synthetic organic molecules, which are often used in medicinal chemistry [1]. Fenoprofen for instance is one of the synthetic diarylethers [2] with nonsteroidal anti-inflammatory, analgesic and antirheumatic effects [3]. More precisely, it is a derivative of 2-aryl propanoic acids, which is an important class of nonsteroidal anti-inflammatory drugs including flurbiprofen, ibuprofen, naproxen and fenoprofen.

Moreover, benzophenone analogues, such as ketoprofen, recently have been reported also as potent anti-inflammatory agents by inhibiting prostaglandin (PG) production [4, 5]. It has been shown that benzoylphenyl acetic acid for instance has anti-inflammatory activity by decreasing the volume of paw edema in treated rats [6].

On the other hand, the introduction of fluorine into organic molecules may cause profound pharmacological effects by improving the activity and selectivity of the bioactive molecules [7]. The utility of fluorine in the design of drugs results mainly from its ability to modify some functional activities, such as increasing lipophilicity [8] and extending its bioavailability [9]. Moreover, carbon forms stronger bond with fluorine (CF)_n_, with a higher oxidative and thermal stability than a carbon–hydrogen bond [10]. The CF_2_ unit for instance is generally considered as a bioisostere of the oxygen atom or of a carbonyl group [11].

We therefore synthesized new *gem*-difluorobisarylic derivatives and evaluated their anti-inflammatory effects. We first investigated their effects on PGE_2_ production in mouse primary macrophages in response to lipopolysaccharide (LPS) and their anti-cyclooxygenase (COX)-1 and -2 activities. We next studied their effects on the production of the pro-inflammatory nitric oxide (NO) and interleukin (IL)-6 and the expression of NO synthase-II (NOS-II) and COX-2.

## Results and discussion

### Synthesis of bisarylic derivatives

**Scheme 1:**
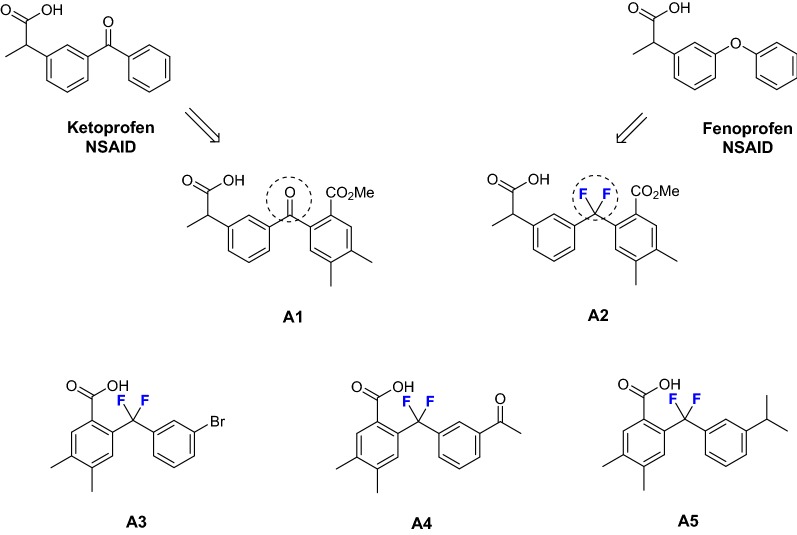
Design of target molecules

Based on our previous study [12], five bisarylic compounds **A1** to **A5** were designed as indicated in Scheme 1.

In our strategy, the *gem*-difluoro unit has been chosen as a mimic of either the ether oxygen (fenoprofen series) or of a carbonyl group (ketoprofen series). First, two phenylpropionic acid derivatives **A1** (as a non-fluorinated reference) and the corresponding *gem*-difluoro derivative **A2** were proposed as analogues of fenoprofen and ketoprofen. The comparison of the inhibitory activities of compounds **A1** and **A2** would allow establishing the impact of fluorine atom on the efficiency of these compounds. On the other hand, three other derivatives **A3**, **A4**, and **A5** were designed as simplified benzoic acid-type derivatives, with three different substituents in *meta* position on the second aromatic ring (Scheme 1).

### Synthetic procedures

**Scheme 2:**
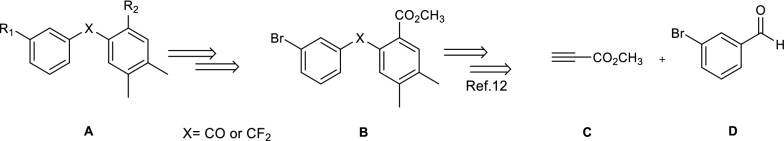
Retrosynthetic analysis for the preparation of compounds **A**

All these molecules were synthesized from bromo intermediates **B** (Scheme 2, Table 1) and were tested for their anti-inflammatory activity.

**Table 1:** Preparation of five bisarylic compounds 
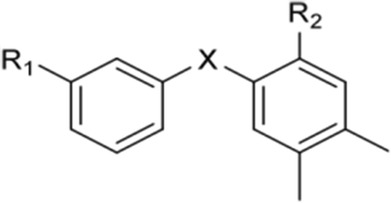

Compound	X	R_1_	R_2_
**A1**	C=O		–CO_2_CH_3_
**A2**	CF_2_		–CO_2_CH_3_
**A3**	CF_2_	Br	–CO_2_H
**A4**	CF_2_	–COCH_3_	–CO_2_H
**A5**	CF_2_	–CH(CH_3_)_2_	–CO_2_H

### Synthesis of key intermediates **7** and **10**

**Scheme 3:**
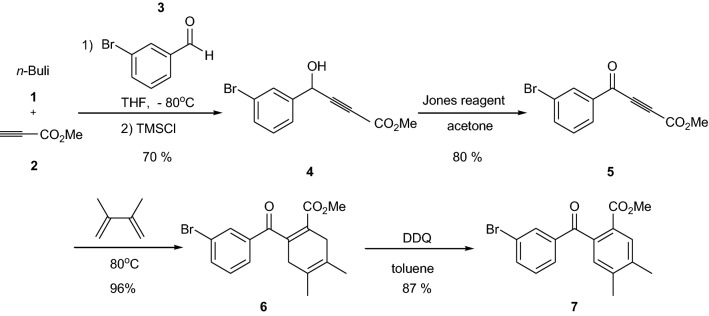
Synthesis of the non-fluorinated key intermediate **7**

Addition of the lithium salt of compound **2** to 3-bromobenzaldehyde **3** at low temperature (− 80 °C) gave propargyl alcohol **4** in 70% yield. After oxidation with Jones reagent, propargylic ketone **5** was isolated in 80% yield. Then, Diels–Alder reaction and DDQ aromatization provided the intermediate **7** with a good yield for both steps (Scheme 3).

**Scheme 4:**
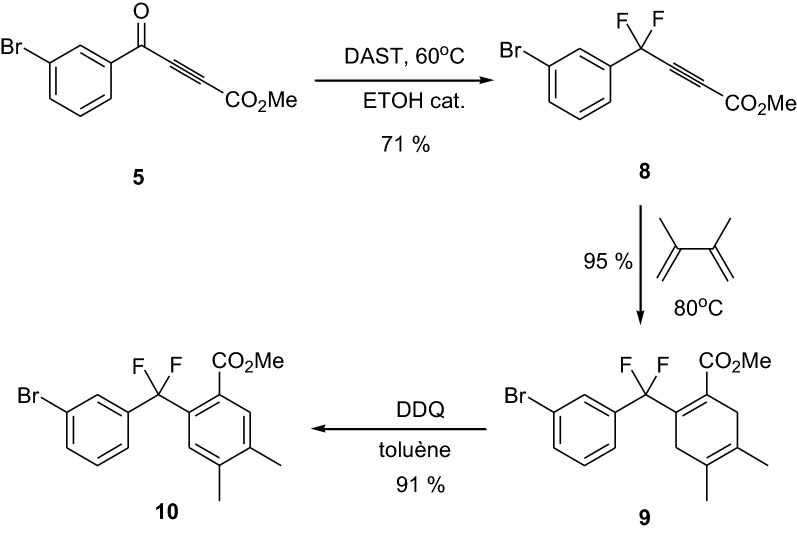
Synthesis of the fluorinated key intermediate **10**

After treatment of ketone **5** by DAST, compound **8** was obtained in 71% yield. Similarly, Diels–Alder reaction and DDQ aromatization proceeded well by giving the fluorinated intermediate **10** with excellent yields (Scheme 4).

### Preparation of compounds **A1** and **A2**

**Scheme 5:**
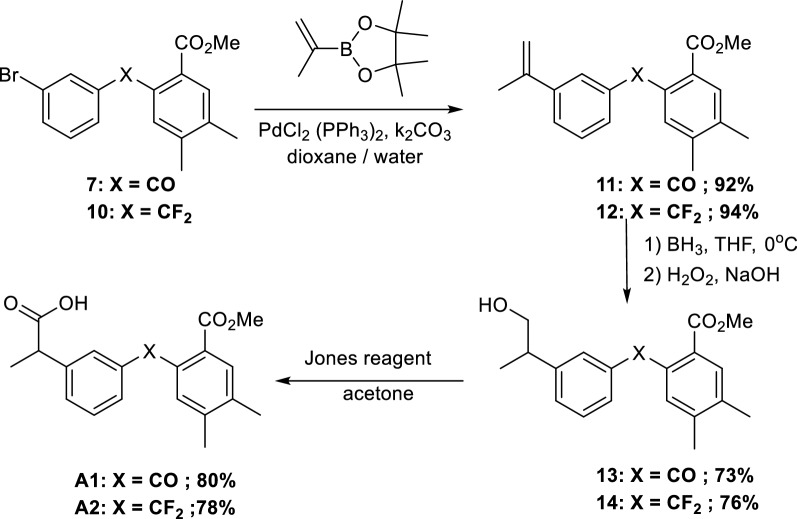
Synthesis of derivatives **A1** and **A2**

Starting from the key scaffolds **7** and **10**, Suzuki–Miyaura couplings, with boronic acid, afforded biphenyl type compounds **11** and **12** in 92 and 94% yield, respectively. Then, hydroboration to **13** and **14**, followed by oxidation with Jones reagent led to the desired analogues **A1** and **A2** in good yields (Scheme 5).

### Preparation of compounds **A3**, **A4**, and **A5**

**Scheme 6:**
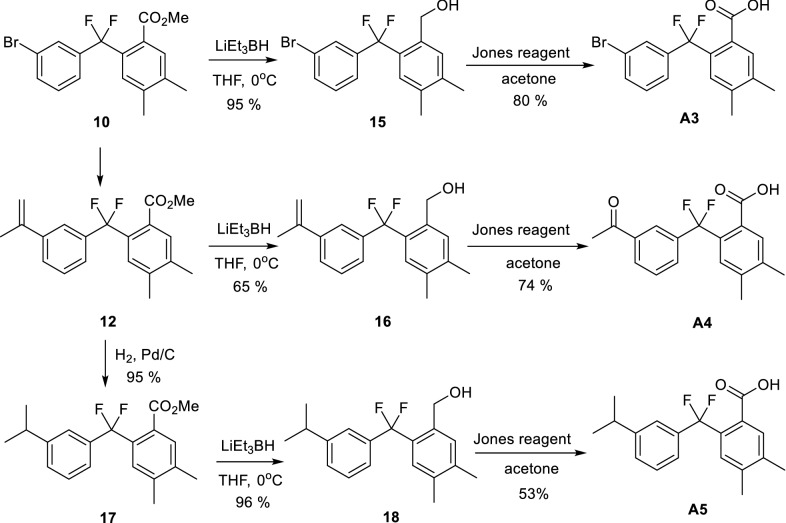
Synthesis of *gem*-diaryl derivatives **A3**, **A4**, and **A5**

Starting from intermediates **10** and **12**, reduction with LiBEt_3_H furnished alcohols **15** and **16** respectively, then oxidation by Jones reagent gave the desired acid **A3**. However, in the case of **16**, an unexpected cleavage of the double bond occurred, affording acid **A4**. Using the same *gem*-difluoro intermediate **12**, catalytic hydrogenation to **17**, followed by reduction and oxidation afforded the desired derivative **A5** in good yields (Scheme 6).

### Biological activities

**Fig. 1:**
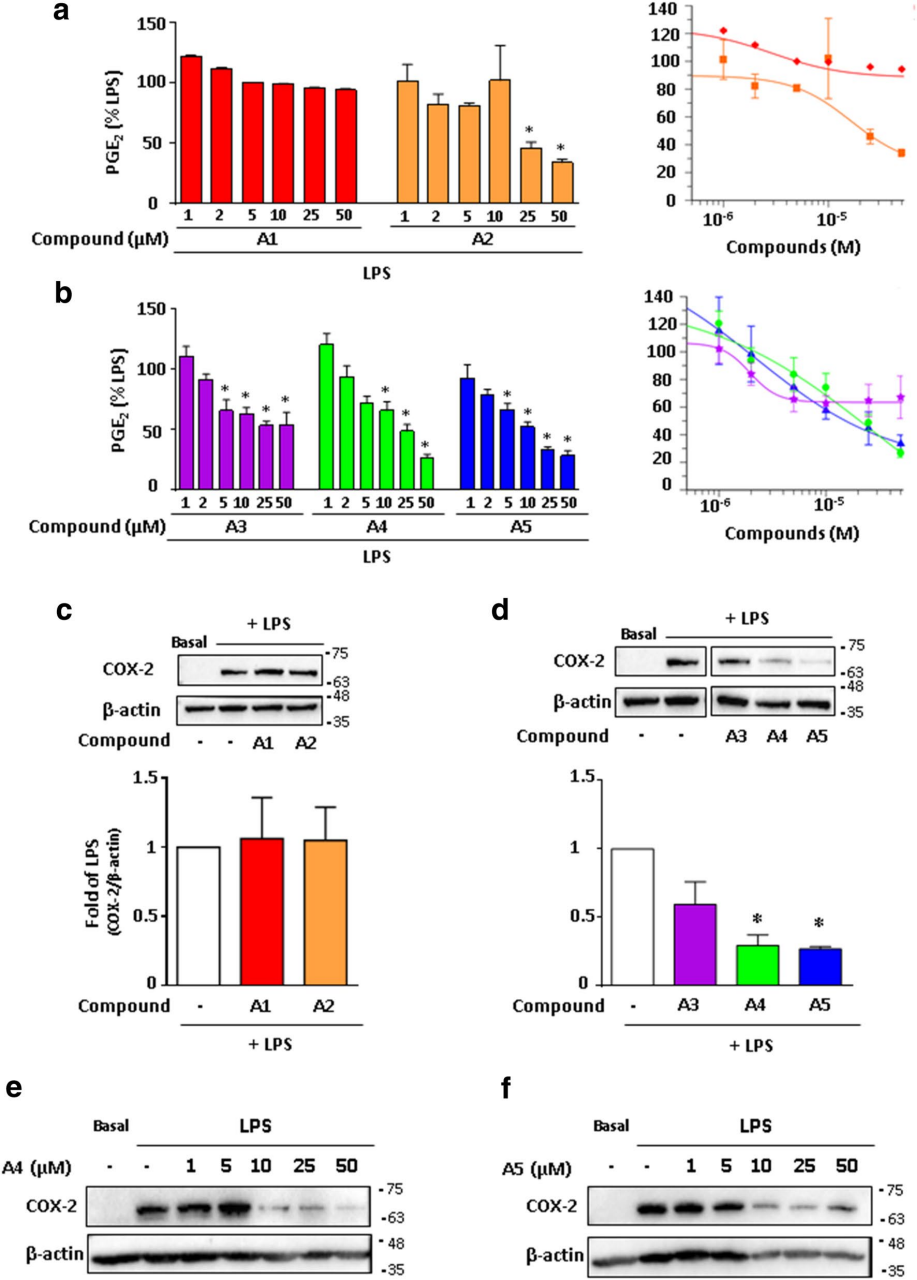
Effects of the *gem*-difluorobisarylic derivatives on PGE_2_ production and COX-2 expression in activated macrophages. BMDM were treated with 6 increasing concentrations, prior to the addition of 10 ng/mL LPS for 24 h. PGE_2_ secretion was measured and expressed as percentage of LPS for **a** compounds **A1** and **A2** and **b** compounds **A3**, **A4** and **A5**. Corresponding IC_50_ fitting curves are shown. **c**, **d** COX-2 and β-actin expression in basal and LPS-treated BMDM with 50 µM of all compounds. Results are obtained from the same blot. Protein bands for basal or LPS-treated macrophages, in the absence of inhibitors, as shown in **c** and **d**, are identical for illustration purpose. Dose–response effect of compounds **e A4** and **f A5** on COX-2 expression. β-actin was used as loading control. Ratio of COX-2/β-actin was calculated after densitometry analysis using ImageJ software. Data are represented as mean ± SEM (n = 4), *p < 0.05 versus LPS (One-way Anova followed by the Dunnett’s test)

We investigated the effects of these derivatives on inflammation in bone marrow-derived macrophages (BMDM) by first evaluating their capacity to decrease LPS-dependent increase of PGE_2_ secretion and COX-2 expression. Compound **A1** (non-fluorinated) and compound **A2** (fluorinated) effects were compared to evaluate the importance of the fluorine atom. Only compound **A2** inhibited significantly in a dose-dependent manner the secretion of PGE_2_ (Fig. 1a) (IC_50_ = 16.5 ± 8.9 µM) with no effect on COX-2 expression (Fig. 1c) supporting the importance of fluorine in inhibiting PGE_2_ production.

In parallel, we compared the inhibitory effects of compounds **A3**, **A4** and **A5**, which are all fluorinated but present differences in R1 group (Table 1). Compound **A3** is the bromine intermediate obtained in the synthetic reaction of compound **A5**. Compound **A4** is a ketone intermediate obtained unexpectedly with good yield during the synthesis of compound **A5**. Compounds **A3**, **A4** and **A5** have the carboxyl group attached to the benzene ring in the ortho position relative to CF_2_ group.

**Fig. 2:**
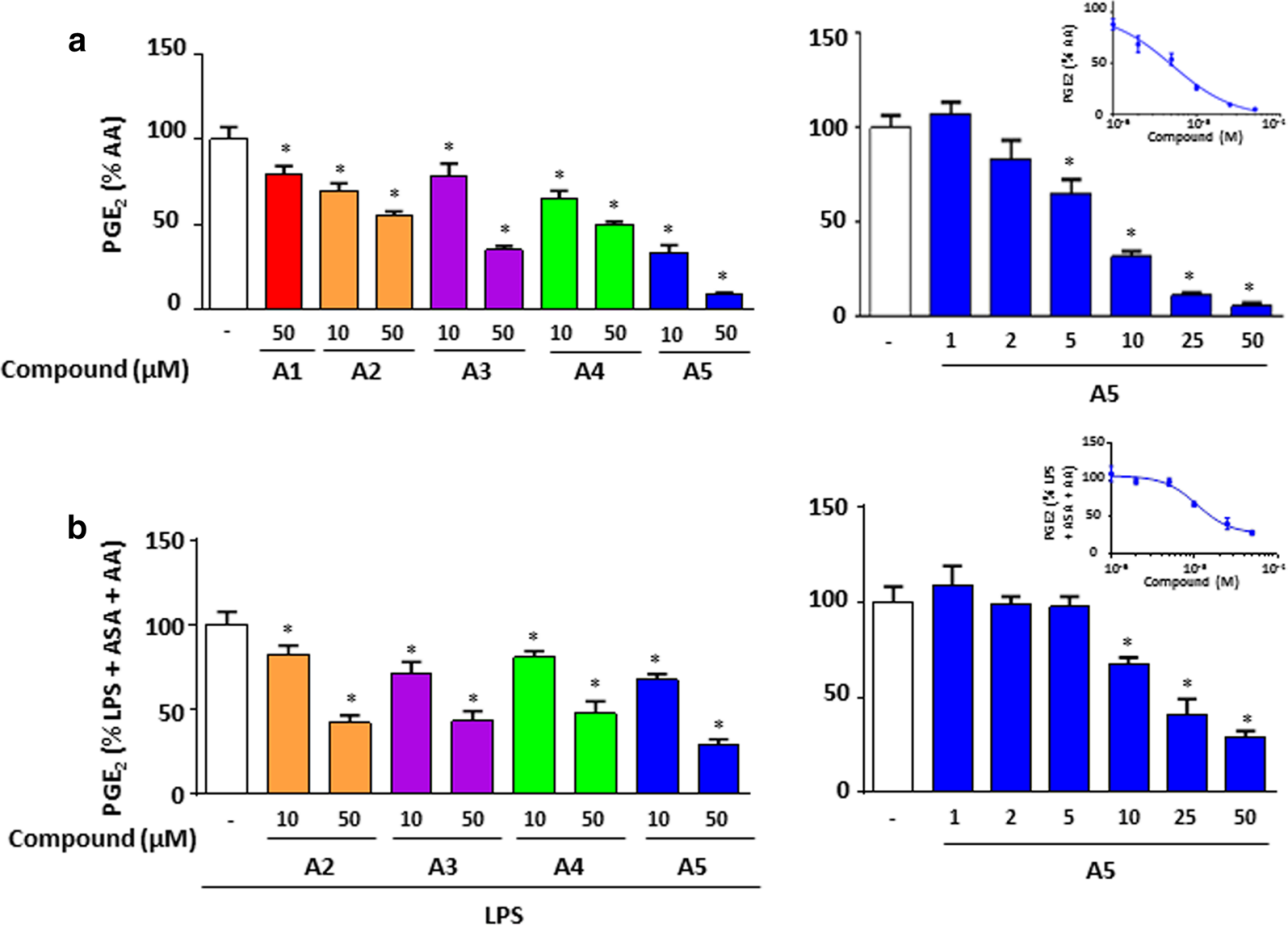
Effects of the *gem*-difluorobisarylic derivatives on COX-1 and COX-2 activity. **a** COX-1 activity. HEK-293 cells overexpressing recombinant COX-1 were treated with 10 and 50 µM of all compounds for 45 min prior to the addition of 10 µM arachidonic acid (AA). PGE_2_ was measured. **b** COX-2 activity. BMDM cells were treated with 10 µM of ASA for 30 min, washed, and 10 ng/mL LPS was added for 24 h to induce COX-2. Cells were further incubated with 10 and 50 µM of each compound prior to the addition of 10 µM AA. PGE_2_ was measured. Data are represented as mean ± SEM (n = 4), *p < 0.05 versus AA for COX-1 activity, and versus LPS + ASA + AA for COX-2 activity (One-way Anova followed by the Dunnett’s test)

Figure 1b showed a dose response effect of these derivatives on PGE_2_ secretion, in which compounds **A4** and **A5** significantly decreased PGE_2_ secretion at 25 and 50 µM with IC_50_ of 28.1 ± 22.8 and 22.4 ± 21.5 µM, respectively. Compound **A3** did not show a strong inhibition at similar concentrations. Under these conditions, only compounds **A4** and **A5** significantly downregulated COX-2 expression (Fig. 1d). Further analysis showed a dose-dependent inhibitory effect on COX-2 expression for compounds **A4** and **A5** (Fig. 1e and f, respectively). Thus, the nature of R groups in compounds **A4** and **A5** is important for their inhibitory effect on COX-2 expression and consequently PGE_2_ production. We next addressed the question whether COX activity was inhibited. We performed COX-1 activity using Human Embryonic kidney (HEK)-293 cells stably overexpressing COX-1. Cells were treated with all compounds at 10 and 50 µM and PGE_2_ was measured after the addition of arachidonic acid (AA). The results showed that compound **A5** had the maximal inhibitory effect on COX-1 activity with more than 80% inhibition at 50 µM (Fig. 2a) with an IC_50_ of 5.2 µM.

COX-2 activity was also assessed on BMDM treated for 30 min with aspirin to inhibit basal COX activity prior to the addition of 10 ng/mL LPS for 24 h which induces COX-2. These cells were then treated with 10 and 50 µM of derivatives and further incubated with AA. PGE_2_ production revealed that compound **A5** inhibited strongly COX-2 activity with an IC_50_ of 13.3 µM, whereas moderate effect was observed for compounds **A2**, **A3** and **A4** (Fig. 2b). Indeed, the assay used for COX-2 activity cannot exclude an effect on mPGES-1.

**Fig. 3:**
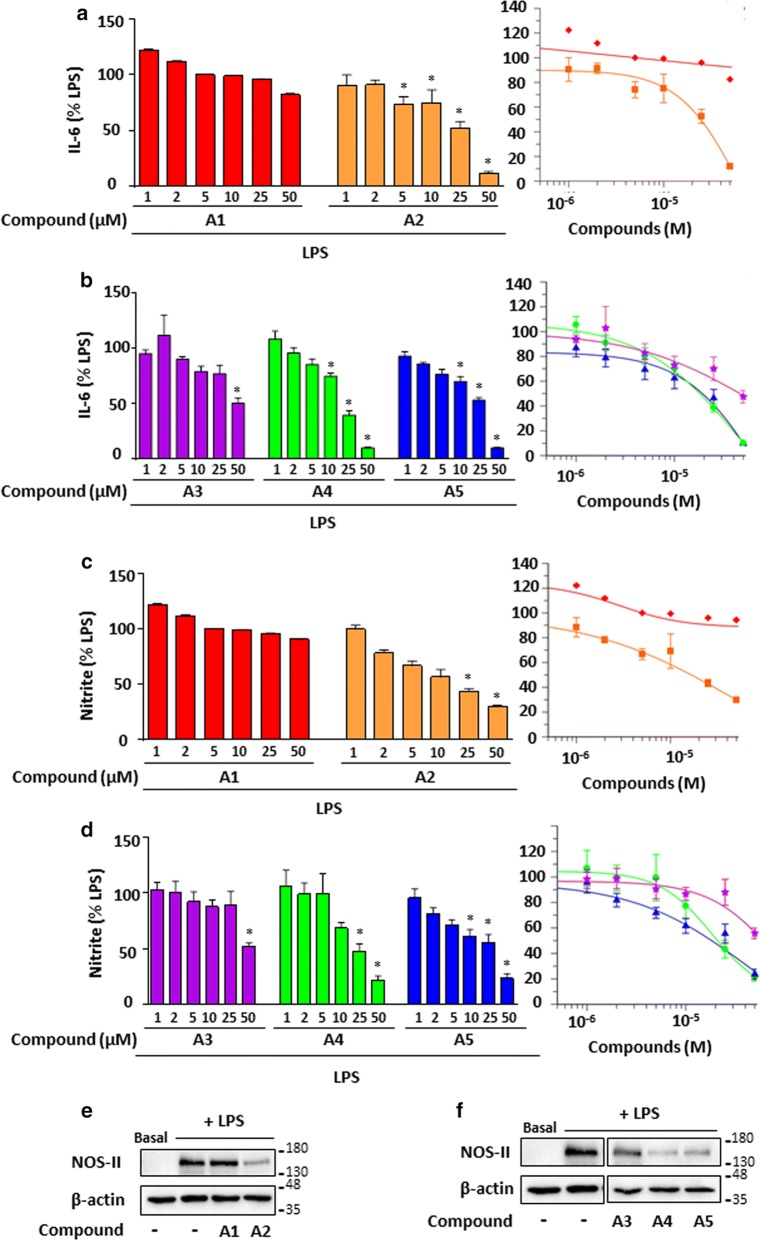
Effects of the *gem*-difluorobisarylic derivatives IL-6 and nitrite, and NOS-II expression. BMDM were treated with 6 increasing concentrations of all compounds prior to the addition of 10 ng/mL LPS for 24 h. IL-6 and NO production was measured and expressed as percentage of LPS for **a**, **c** compounds **A1** and **A2**, and **b**, **d**, compounds **A3**, **A4** and **A5**, respectively. Corresponding IC_50_ fitting curves are shown. **e**, **f** NOS-II and β-actin expression in basal and LPS-treated BMDMs with 50 µM of all compounds. Results are obtained from the same blot. Protein bands for basal or LPS-treated in macrophages in the absence of inhibitors, as shown in **e** and **f**, are identical for illustration purpose. β-actin was used as loading control. Data are represented as mean ± SEM (n = 4), *p < 0.05 versus LPS (One-way Anova followed by the Dunnett’s test)

In parallel, we assessed the effect of these compounds on the production of IL-6 and NO measured by its breakdown product nitrite. Figure 3 showed a dose response inhibition of compounds **A2** (Fig. 3a, c), **A4** and **A5** (Fig. 3b, d) for both IL-6 and NO secretion. IC_50_ are presented in Table 2 and were significant for compounds **A4** and **A5**.

**Table 2:** In vitro inhibition activity of compounds **A1**, **A2**, **A3**, **A4** and **A5** on inflammatory mediators in macrophages

Compounds	IC_50_ (μM)	
	IL-6	NO
**A1**	ND	ND
**A2**	64.5 ± 21.1^a^	45.2 ± 24.6
**A3**	60 ± 60.2	ND
**A4**	51.2 ± 17.4	18.5 ± 2.7
**A5**	82.6 ± 24.0	40.9 ± 25.4
**Fenoprofen**	300 ± 100	43.8 ± 39.2

*ND* not determined

^a^Mean ± SEM

NOS-II is the inducible form of nitric oxide synthase, and is responsible for the production of the measured NO. For this, NOS-II protein expression was analyzed in LPS-stimulated BMDM, treated with 50 µM of bisarylic derivatives for 24 h. Results revealed that the fluorinated compounds **A2**, **A4** and **A5** inhibited NOS-II expression in parallel to NO production (Fig. 3e, f).

## Molecular docking

**Fig. 4:**
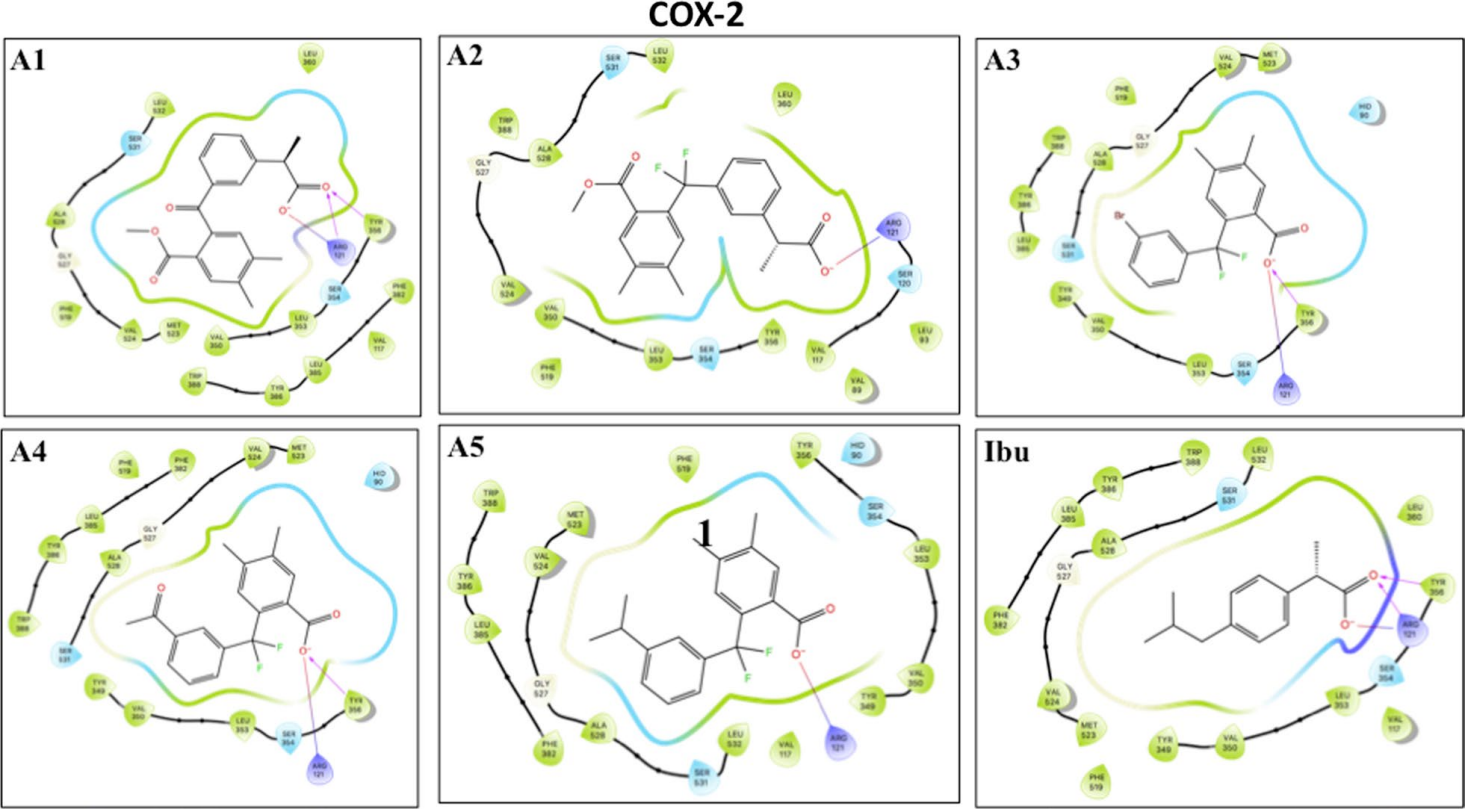
Two-dimensional pose of compounds** A1** to** A5** and Ibuprofen inside the binding pocket of mouse COX-2 as crystallized by [13]. Ligand-receptor interactions as highlighted by Maestro (Shrodinger, LLC). Ligands are represented in stick, and amino acids within the binding pocket are labeled. An arrow represents the H-bonds between an amino acid and ligand groups. A line shows a potential salt bridge between two charged groups

**Fig. 5:**
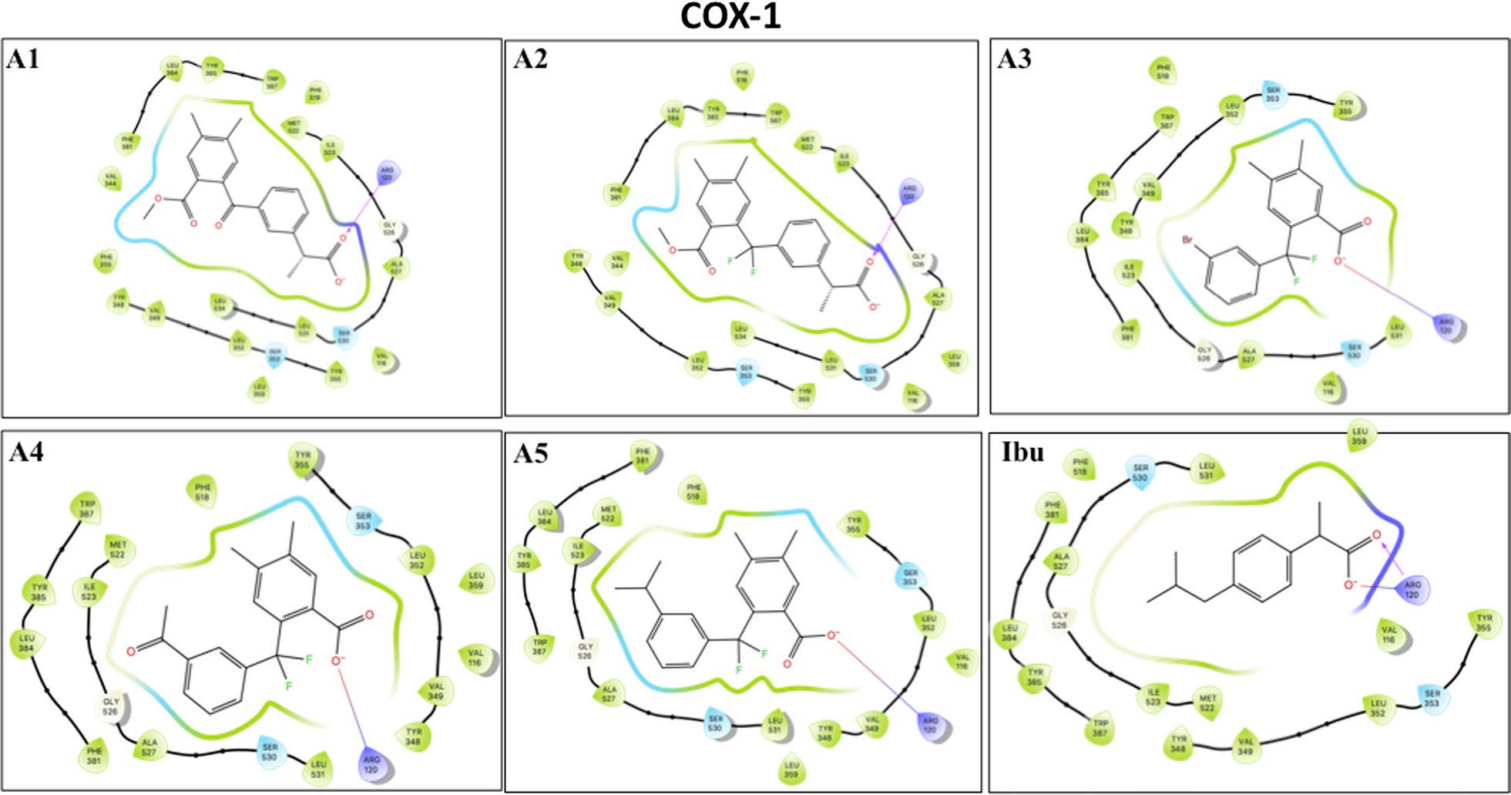
Two-Dimensional pose of compounds **A1** to **A5** and Ibuprofen inside the binding pocket of human COX-1 as crystallized by [14]. Ligand receptor interactions were evaluated for COX-1 as described in legend for Fig. 4

We finally carried out model analysis of the inhibitors with ovine COX-1 [13] and murine COX-2 [14], to examine how these compounds dock with the active sites of the enzymes and to determine the amino acids involved in the interaction with the compounds. Ibuprofen docked into the hydrophobic cavity of COX-2 formed by Arg121, Tyr356, Ser354, Leu353, Val350 and Tyr349, where the carboxyl group of ibuprofen interacts with Arg121 and Tyr356 by a salt bridge and a hydrogen bond. The compounds **A3**, **A4** and **A5** were docked near Arg121, similarly to ibuprofen. Compounds **A3** and **A4** showed interaction with Tyr356 (Fig. 4). The binding scores of compounds **A3**, **A4** and **A5** (− 7.7 kcal/mol, − 7.7 kcal/mol and − 7.5 kcal/mol respectively) are comparable to ibuprofen (Table 3). Furthermore, the difluoromethyl group present in these compounds, which introduces a strong electrostatic field in this hydrophobic pocket, would be in favor the interaction with Arg121. Compounds **A1** and **A2**, even though they occupy the same active pocket, interact with Arg121 through the carboxylate group with less binding energy, have a bulky side chains that negatively would affect the stability of these molecules in the hydrophobic pocket. For COX-1, ibuprofen docked into the hydrophobic pocket composed of the amino acids Arg120, Tyr355, Ser353, Leu352, Val349, Tyr348, Val116, Leu531, Ser530, Ala527, Gly526 and Ile523. All compounds docked in the same active hydrophobic pocket of COX-1. Only Arg120 interacts with the carboxylate group by a salt bridge (Fig. 5). Similarly, to ibuprofen, compounds **A2**, **A3**, **A4** and **A5** showed a moderate binding energy compared to ibuprofen (− 7 kcal/mol and −  7.8 kcal/mol respectively, Table 3) whereas compound **A1** showed the lowest binding energy, which is compatible with biological activities.

**Table 3:** Comparison of COX-1 and COX-2 molecular docking data

Compound	COX-1 kcal/mol	COX-2 kcal/mol
**A1**	− 6.5	− 6.6
**A2**	− 7	− 6.7
**A3**	− 7	− 7.7
**A4**	− 7	− 7.7
**A5**	− 7	− 7.5
**Ibuprofen**	− 7.8	− 7.7

More analyses are required to fully understand the key role of the fluorine atoms on the biological activity of these molecules. However, to explain these results, it is possible that the bulky and lipophilic CF_2_ group could fit better in the pocket of these proteins than the carbonyl of ketoprofen or the oxygen atom of fenoprofen. Further, in the case of compounds **A3**, **A4** and **A5** it can also increase the acidity of the CO_2_H in ortho position.

## Conclusion

In conclusion, five bisarylic derivatives were prepared and tested in comparison with fenoprofen. This type of compounds is endowed with certain anti-inflammatory activities in mouse primary macrophages with a significant difference between the fluorinated analogues and the non-fluorinated one, showing the importance of the CF_2_ group. All fluorinated derivatives blocked PGE_2_, nitrite and IL-6 production in activated macrophages. Derivatives **A4** and **A5** showed additional strong inhibition of COX-2 and NOS-II expression. In addition, derivatives **A3**, **A4** and **A5** showed better anti-inflammatory activities than the other derivatives, with compound **A5** having COX-1 and COX-2 direct inhibitory activities. Molecular docking of the compounds COX-1 and COX-2 are in support of the biological activity.

## Methods

### Chemistry experimental part

Reactions were carried out as described previously and monitored as described by ^19^F NMR and by thin-layer chromatography (TLC) [15]. Yields refer to chromatographically and spectroscopically (^1^H, ^13^C, and ^19^F NMR) homogeneous materials. Nuclear magnetic resonance (NMR) spectra have been recorded as previously described [15]. Mass spectral analyses have been performed at the Centre Régional de Mesures Physiques de l’Ouest (CRMPO) in Rennes (France).

### Synthesis of methyl 4-(3-bromophenyl)-4-hydroxybut-2-ynoate 4

To a solution of methylpropiolate (2.6 mL, 29.20 mmol, 1.2 equiv.) in anhydrous THF (20 mL) cooled at − 90 °C and set under nitrogen, *n*-BuLi (11.4 mL, 2.5 M, 1.2 equiv.) was added dropwise. The reaction mixture was stirred for 30 min at T − 80 °C before the dropwise addition of a solution of 3-bromobenzaldehyde (4 g, 21.60 mmol) in anhydrous THF (20 mL). After stirring for additional 20 min at the same temperature, TMSCl (7 mL, 55.00 mmol, 2.5 equiv.) was added dropwise to the reaction mixture that was stirred for 1 h at − 80 °C and then left to rise at room temperature while continuous stirring for additional 2 h. The mixture was treated with concentrated solution of NH_4_Cl, extracted with ethyl acetate (3 times), dried over Na_2_SO_4_ and concentrated by evaporating the solvent. Alcohol **4** was isolated over silica gel by column chromatography.

### Synthesis of methyl 4-(3-bromophenyl)-4-oxobut-2-ynoate 5

To alcohol **4** (2.1 g, 7.46 mmol) in acetone (18 mL) was added dropwise under magnetic stirring at room temperature, a concentrated (5.4 M) solution of Jones reagent until disappearance of the starting material (TLC analysis). After addition of isopropanol (5 equiv.), the reaction mixture was filtered, and the filtrate was extracted with ethyl acetate. The combined organic phases were dried over Na_2_SO_4_, filtered and concentrated in vacuum. After purification by chromatography on silica gel, ketone **5** was obtained.

### Synthesis of methyl 4-(3-bromophenyl)-4,4-difluorobut-2-ynoate 8

To propargylic ketone **5** (350 mg, 1.31 mmol) were added one drop of 95% ethanol and DAST (1.05 mL, 7.96 mmol, 6 equiv.). The reaction mixture was stirred at 60 °C for 7 h. After coming back to room temperature and hydrolysis, the reaction mixture was extracted with ethyl acetate (3 times). The organic layers were separated, washed with water (3 times), dried over Na_2_SO_4_ and concentrated under vacuum. After purification by chromatography on silica gel, fluorinated compound **8** was obtained.

### Synthesis of methyl 2-(3-bromobenzoyl)-4,5-dimethylcyclohexa-1,4-dienecarboxylate 6 and methyl 2-((3-bromophenyl) difluoromethyl)-4,5-dimethylcyclohexa-1,4-dienecarboxylate 9

Difluoro propagylic ester (1.54 mmol) and 2,3-dimethyl-1,3-butadiene (14 equiv.) were refluxed neat at nearly 80 °C. The reaction was controlled by ^19^F NMR after 5 h and was stopped by that time. Finally, the unreacted butadiene was evaporated. After purification by column chromatography on silica gel, cyclohexadienes **6** and **9** were isolated.

### Synthesis of methyl 2-(3-bromobenzoyl)-4,5-dimethylbenzoate 7 and methyl 2-((3-bromophenyl) difluoromethyl)-4,5-dimethylbenzoate 10

A solution of the cyclohexadiene (2.18 mmol) and DDQ (1.2 equiv.) in toluene (7 mL) was stirred at 42 °C for 2 h. The reaction mixture was filtered on silica gel and the residues were washed with ethyl acetate. The organic phase was concentrated in vacuo and compounds **7** and **10** were isolated by chromatography on silica gel.

### Synthesis of methyl 4,5-dimethyl-2-(3-(prop-1-en-2-yl) benzoyl) benzoate 11 and methyl 2-(difluoro(3-(prop-1-en-2-yl) phenyl) methyl)-4,5-dimethylbenzoate 12

A solution of bromo-ester (1.74 mmol), isopropenylboronic acid pinacol ester (2 equiv.), palladium dichlorobistriphenylphosphine (5% mol) and potassium carbonate (2 equiv.) in a 5/1 mixture of dioxane and water (15/3 mL) was stirred at 90 °C for 20 h. The reaction mixture was extracted by ethyl acetate (3 times). The combined organic phases were washed with water, dried over Na_2_SO_4_ and concentrated in vacuo. After purification by chromatography on silica gel, the compounds **11** and **12** were isolated.

### Synthesis of methyl 2-(3-(1-hydroxypropan-2-yl)benzoyl)-4,5-dimethylbenzoate 13 and methyl 2-(difluoro(3-(1-hydroxypropan-2-yl)phenyl)methyl)-4,5-dimethylbenzoate 14

To the alkene (0.72 mmol) in anhydrous THF (5 mL) was added, dropwise under magnetic stirring and under N_2_ at 0 °C, a solution of BH_3_ in THF (5.5 equiv.). The reaction mixture was stirred overnight at room temperature. After 24 h, the mixture was oxidized by addition of H_2_O_2_ 30% (4.4 equiv.) and NaOH 3 M (4.4 equiv.) and was stirred for 2 h. The organic phase was separated, while the aqueous phase was extracted by ethyl acetate. The organic fractions were collected, dried over Na_2_SO_4_, and concentrated in vacuo. After purification by flash chromatography on silica gel, alcohols **13** and **14** were isolated.

### Synthesis of (2-((3-bromophenyl)difluoromethyl)-4,5-dimethylphenyl)methanol 15, (2-(difluoro(3-(prop-1-en-2-yl)phenyl)methyl)-4,5-dimethylphenyl)methanol 16 and (2-(difluoro(3-isopropylphenyl)methyl)-4,5-dimethylphenyl)methanol 18

To the ester (0.27 mmol) in anhydrous THF (4 mL) was added, dropwise under magnetic stirring and under N_2_ at 0 °C, a 1 M solution of LiEt_3_BH in THF (2.5 equiv.). The reaction mixture was stirred at 0 °C for 15 min and then quenched by addition of a saturated NH_4_Cl solution. The organic phase was separated, while the aqueous phase was extracted by ethyl acetate. The organic fractions were collected, dried over Na_2_SO_4_, and concentrated in vacuo. After purification by flash chromatography on silica gel, alcohols **15**, **16** and **18** were isolated.

### Synthesis of methyl 2-(difluoro(3-isopropylphenyl)methyl)-4,5-dimethylbenzoate 17

To a solution of **12** (498 mg, 1.51 mmol) in AcOEt (15 mL), was added 50 mg of palladium-charcoal catalyst (10%). The mixture was stirred at room temperature under hydrogen atmosphere. After 2 h, it was filtered and compound **17** was obtained, after purification on silica gel.

The physicochemical properties and the spectral data of intermediates **4**–**18** are presented in the Tables 4 and 5, respectively and in Tables 6 and 7 for the synthesized bisarylic derivatives **A1** to **A5**.

**Table 4:** The physicochemical properties of intermediates **4**–**18**

Compound	IUPAC Name	Aspect	Mass of the starting material	Mass of the product	*Rf* value (PE:EtOAc)	m.p. (^o^C)	% yield
**4**	Methyl 4-(3-bromophenyl)-4-hydroxybut-2- ynoate	Yellow oil	4 g	3.9 g	0.30 (8:2)	–	70
**5**	Methyl 4-(3-bromophenyl)-4-oxobut-2-ynoate	Yellow solid	2.10 g	1.59 g	0.40 (9:1)	102–104	80
**6**	Methyl 2-(3-bromobenzoyl)-4,5-dimethylcyclohexa-1,4-dienecarboxylate	Yellow solid	410 mg	560 mg	0.43 (9:1)	100–102	96
**7**	Methyl 2-(3-bromobenzoyl)-4,5-dimethylbenzoate	Yellow solid	937 mg	810 mg	0.34 (9:1)	122–124	87
**8**	Methyl 4-(3-bromophenyl)-4,4-difluorobut-2-ynoate	Colorless oil	350 mg	270 mg	0.44 (9:1)	–	71
**9**	Methyl 2-((3-bromophenyl)difluoromethyl)-4,5-dimethylcyclohexa-1,4-dienecarboxylate	White solid	757 mg	925 mg	0.44 (9:1)	72–74	95
**10**	Methyl 2-((3-bromophenyl)difluoromethyl)-4,5-dimethylbenzoate	Yellow solid	808 mg	731 mg	0.51 (9:1)	60–62	91
**11**	Methyl 4,5-dimethyl-2-(3-(prop-1-en-2-yl)benzoyl)benzoate	White solid	782 mg	640 mg	0.34 (9:1)	82–48	92
**12**	Methyl 2-(difluoro(3-(prop-1-en-2-yl)phenyl)methyl)-4,5-dimethylbenzoate	Yellow oil	640 mg	539 mg	0.35 (9:1)	–	94
**13**	Methyl 2-(3-(1-hydroxypropan-2-yl)benzoyl)-4,5-dimethylbenzoate	Yellow oil	400 mg	309 mg	0.42 (7:3)	–	73
**14**	Methyl 2-(difluoro(3-(1-hydroxypropan-2-yl)phenyl)methyl)-4,5-dimethylbenzoate	Yellow oil	240 mg	192 mg	0.36 (7.5:2.5)	–	76
**15**	(2-((3-bromophenyl)difluoromethyl)-4,5-dimethylphenyl)methanol	White solid	100 mg	88 mg	0.26 (9:1)	62–64	95
**16**	(2-(difluoro(3-(prop-1-en-2-yl)phenyl)methyl)-4,5-dimethylphenyl)methanol	Yellow oil	148 mg	88 mg	0.37 (8:2)	–	65
**17**	Methyl 2-(difluoro(3-isopropylphenyl)methyl)-4,5-dimethylbenzoate	White solid	498 mg	476 mg	0.72 (9:1)	116–118	95
**18**	(2-(difluoro(3-isopropylphenyl)methyl)-4,5-dimethylphenyl)methanol	White solid	294 mg	260 mg	0.69 (9:1)	80–82	96

**Table 5:** Spectral data of intermediates **4**–**18**

Compound	^1^H NMR	^13^C NMR	^19^F NMR
**4**	**CDCl**_**3**_**, 300** **MHz**: 7.56 (m, 1H), 7.36 (m, 2H), 7.18 (m, 1H), 5.44 (s, 1H), 3.70 (s, 3H), 3.64 (br. s, 1H)	**CDCl**_**3**_**, 75** **MHz**: 153.7, 140.5, 131.8, 130.2, 129.6, 125.1, 122.7, 86.0, 77.6, 63.2, 53.0	
**5**	**CDCl**_**3**_**, 300** **MHz**: 8.02 (t, 1H, *J* = 1.7 Hz), 7.86 (ddd, 1H, *J* = 7.9, 1.7 and 1.0 Hz), 7.61 (ddd, 1H, *J *= 7.9, 1.7 and 1.0 Hz), 7.23 (t, 1H, *J *= 7.9 Hz), 3.73 (s, 3H)	**CDCl**_**3**_**, 75** **MHz**: 173.6, 151.4, 137.0, 136.1, 131.3, 129.5, 127.4 (3C), 79.7, 78.3, 52.6.	
**6**	**CDCl**_**3**_**, 300** **MHz**: 8.01 (t, 1H, *J* = 1.9 Hz), 7.80 (m, 1H), 7.68 (m, 1H), 7.33 (t, 1H, *J *= 7.8 Hz), 3.53 (s, 3H), 2.97–2.98 (m,4H), 1.74 (s, 3H), 1.67 (s, 3H)	**CDCl**_**3**_**, 75** **MHz**: 197.1, 165.9, 146.8, 137.0, 136.0, 131.2, 130.3, 127.1, 125.6, 123.1, 123.0, 120.6, 51.8, 36.5, 32.6, 18.2, 17.8	
**7**	**CDCl**_**3**_**, 300** **MHz**: 7.89 (t, 1H, *J* = 1.7 Hz). 7.81 (s, 1H), 7.62 (m, 2H), 7.27 (t, 1H, *J *= 7.9 Hz), 7.13 (s, 1H), 3.61 (s, 3H), 2.36 (s, 3H), 2.33 (s, 3H)	**CDCl**_**3**_**, 75** **MHz**: 195.9, 166.3, 142.1, 139.3, 138.8, 138.6, 135.6, 131.8, 131.1, 129.9, 128.8, 127.7, 126.4, 122.7, 52.0, 19.9, 19.6	
**8**	**CDCl**_**3**_**, 300** **MHz**: 7.70 (s, 1H), 7.55 (m, 1H), 7.49 (m, 1H), 7.26 (t, 1H, *J* = 8.0 Hz), 3.76 (s, 3H)	**CDCl**_**3**_**, 75** **MHz**: 152.1 (t, ^*4*^*J* = 2.4 Hz), 136.2 (t, ^*2*^*J* = 27.5 Hz), 134.5 (t, ^*4*^*J *= 1.6 Hz), 130.4, 128.3 (t, ^*3*^*J* = 5.0 Hz), 123.9 (t, ^*3*^*J* = 4.9 Hz), 122.7, 110.4 (t, ^*1*^*J* = 236.3 Hz), 78.4 (t, ^3^*J* = 5.9 Hz), 76.5 (t, ^2^*J* = 43.7 Hz), 53.4	**CDCl**_**3**_**, 282** **MHz**: -80.06 (s)
**9**	**CDCl**_**3**_**, 300** **MHz**: 7.70 (s, 1H), 7.50 (s, 1H), 7.48 (s, 1H), 7.23 (m, 1H), 3.68 (s, 3H), 2.86 (t, 2H, *J* = 7.2 Hz), 2.50 (t, 2H, *J* = 7.2 Hz), 1.57 (s, 3H), 1.52 (s, 3H)	**CDCl**_**3**_**, 75** **MHz**: 170.3, 137.7 (t, ^*2*^*J* = 28.4 Hz), 133.2, 130.0, 129.9 (t, ^*3*^*J* = 5.0 Hz), 128.9 (t, ^*3*^*J* = 5.9 Hz), 128.8 (t, ^*2*^*J* = 25.4 Hz), 124.5 (t, ^4^*J* = 5.6 Hz), 122.5, 121.6, 121.2, 119.4 (t, ^*1*^*J* = 244.4 Hz), 52.1, 35.8, 31.6, 18.0, 17.7	**CDCl**_**3**_**, 282** **MHz**: − 93.06 (s)
**10**	**CDCl**_**3**_**, 300** **MHz**: 7.62 (s, 1H), 7.54 (m, 1H), 7.49 (s, 1H), 7.47 (s, 1H), 7.40 (m, 1H), 7.26 (m, 1H), 3.65 (s, 3H), 2.36 (s, 3H), 2.33 (s, 3H)	**CDCl**_**3**_**, 75** **MHz**: 168.2, 140.1 (t, ^*2*^*J* = 28.3 Hz), 140.0, 139.1 (t, ^*4*^*J* = 1.3 Hz), 132.8 (t, ^*4*^*J* = 2.7 Hz), 131.9 (t, ^*2*^*J* = 26.9 Hz), 131.1, 129.7, 129.0 (t, ^*3*^*J* = 5.3 Hz), 128.5 (t, ^*3*^*J* = 7.9 Hz), 128.4 (t, ^*3*^*J* = 3.4 Hz), 124.6 (t, ^3^*J* = 5.0 Hz), 122.1, 119.7 (t, ^*1*^*J* = 242.3 Hz), 52.1, 19.9, 19.4	**CDCl**_**3**_**, 282** **MHz**: –82.83 (s)
**11**	**CDCl**_**3**_**, 300** **MHz**: 7.93 (t, 1H, *J* = 1.6 Hz). 7.80 (s, 1H), 7.64 (ddd, 1H, *J* = 7.7, 1.6, 1.2 Hz), 7.53 (dt, 1H, *J *= 7.7, 1.2 Hz), 7.35 (t, 1H, *J* = 7.7 Hz), 7.18 (s, 1H), 5.38 (m, 1H), 5.12 (m, 1H), 3.56 (s, 3H), 2.37 (s, 3H), 2.34 (s, 3H), 2.15 (m, 3H)	**CDCl**_**3**_**, 75** **MHz**: 197.3, 166.7, 142.4, 141.9, 141.6, 139.2, 138.6, 137.5, 131.0, 129.9, 129.1, 128.6, 128.3, 126.8, 125.8, 113.5, 51.9, 21.7, 19.9, 19.6	
**12**	**CDCl**_**3**_**, 300** **MHz**: 7.55 (s, 1H), 7.42 (m, 1H), 7.38 (s, 1H), 7.36 (s, 1H), 7.26 (m, 1H), 7.24 (m, 1H), 5.29 (m, 1H), 5.03 (m, 1H), 3.54 (s, 3H), 2.25 (s, 3H), 2.23 (s, 3H), 2.06 (m, 3H)	**CDCl**_**3**_**, 75** **MHz**: 168.6, 142.7, 141.2, 139.7, 138.8, 137.8 (t, ^*2*^*J* = 27.6 Hz), 132.5 (t, ^*2*^*J* = 27.3 Hz), 130.7, 128.7 (t, ^*3*^*J* = 3.4 Hz), 128.6 (t, ^*3*^*J* = 7.6 Hz), 128.0, 126.8 (t, ^*4*^*J* = 1.9 Hz), 125.0 (t, ^*3*^*J* = 5.2 Hz), 122.9 (t, ^*3*^*J* = 5.2 Hz), 120.6 (t, ^*1*^*J* = 241.6 Hz), 113.2, 52.0, 21.7, 19.9, 19.3	**CDCl**_**3**_**, 282** **MHz**: – 82.49 (s)
**13**	**CDCl**_**3**_**, 300** **MHz**: 7.77 (s, 1H), 7.67 (t, 1H, *J* = 1.6 Hz), 7.54 (dt, 1H, *J* = 7.5, 1.6 Hz), 7.42 (dt, 1H, *J* = 7.5, 1.6 Hz), 7.36 (t, 1H, *J *= 7.5 Hz), 7.20 (s, 1H), 3.70 (d, 2H, *J* = 6.9 Hz), 3.51 (s, 3H), 2.97 (sext., 1H, *J* = 6.9 Hz), 2.37 (s, 3H), 2.34 (s, 3H), 1.71 (br. s, 1H), 1.26 (d, 3H, *J *= 6.9 Hz)	**CDCl**_**3**_**, 75** **MHz**: 197.3, 167.0, 144.4, 141.9, 139.0, 138.7, 137.8, 132.2, 130.9, 129.2, 128.7, 127.8 (2C), 127.1, 68.4, 51.9, 42.3, 19.9, 19.6, 17.5197.3, 167.0, 144.4, 141.9, 139.0, 138.7, 137.8, 132.2, 130.9, 129.2, 128.7, 127.8 (2C), 127.1, 68.4, 51.9, 42.3, 19.9, 19.6, 17.5	
**14**	**CDCl**_**3**_**, 300** **MHz**: 7.43 (s, 1H), 7.32 (s, 1H), 7.25 (s, 1H), 7.23 (s, 2H), 7.16–7.21 (m, 1H), 3.53 (d, 2H, *J* = 6.9 Hz), 3.46 (s, 3H), 2.83 (sext., 1H, *J* = 6.9 Hz), 2.24 (s, 3H), 2.21 (s, 3H), 2.14 (br. s, 1H), 1.14 (d, 3H, *J *= 6.9 Hz)	**CDCl**_**3**_**, 75** **MHz**: 168.7, 143.9, 139.7, 138.8, 137.7 (t, ^2^*J* = 27.4 Hz), 132.4 (t, ^*2*^*J* = 27.3 Hz), 130.6, 128.8 (t, ^*4*^*J* = 1.7 Hz), 128.7 (t, ^*3*^*J* = 3.5 Hz), 128.3 (t, ^*3*^*J* = 7.7 Hz), 128.2, 125.1 (t, ^*3*^*J* = 5.1 Hz), 124.1 (t, ^*3*^*J* = 5.2 Hz), 120.4 (t, ^*1*^*J* = 241.3 Hz), 68.3, 52.0, 42.2, 19.8, 19.3, 17.3	**CDCl**_**3**_**, 282** **MHz**: – 82.21 (s)
**15**	**CDCl**_**3**_**, 300** **MHz**: 7.52 (m, 1H), 7.74 (m, 1H), 7.28 (m, 1H), 7.26 (m, 1H), 7.14–7.19 (m, 2H), 4.43 (s, 2 H), 2.21 (s, 3H), 2.19 (s, 3H), 1.82 (s, 1H)	**CDCl**_**3**_**, 75** **MHz**: 139.5 (t, ^*4*^*J* = 1.5 Hz),139.5 (t, ^*2*^*J* = 28.8 Hz),136.2 (t, ^*3*^*J* = 2.1 Hz), 135.8, 133.3 (t, ^*4*^*J* = 1.8 Hz), 130.9, 130.4 (t, ^2^*J* = 26.2 Hz), 130.0, 129.1 (t, ^*3*^*J* = 5.2 Hz), 127.8 (t, ^*3*^*J* = 7.9 Hz), 124.7 (t, ^3^*J* = 5.2 Hz), 122.5, 120.8 (t, ^*1*^*J* = 241.8 Hz), 62.0 (t,^*4*^*J* = 3.4 Hz), 19.5 (2C)	**CDCl**_**3**_**, 282** **MHz**: -83.28 (s)
**16**	**CDCl**_**3**_**, 300** **MHz**: 7.53 (s, 1H), 7.45 (m, 1H), 7.29 (s, 1H), 7.25 (m, 1H), 7.21 (s, 2H), 5.29 (s, 1H), 5.05 (t, 1H, *J *= 1.4 Hz), 4.48 (s, 2H), 2.23 (s, 3H), 2.20 (s, 3H), 2.06 (dd, 3H, *J *= 1.4, 0.8 Hz)	**CDCl**_**3**_**, 75** **MHz**: 142.5, 141.6, 139.3, 137.4 (t, ^*2*^*J* = 28.0 Hz), 136.3 (t, ^*4*^*J* = 2.1 Hz), 135.7, 131.3 (t,^*2*^*J* = 26.5 Hz), 131.1, 128.3, 128.0 (t, ^*3*^*J* = 7.8 Hz), 127.3 (t, ^*3*^*J* = 1.8 Hz), 125.1 (t, ^*3*^*J* = 5.2 Hz), 122.9 (t, ^*3*^*J* = 5.2 Hz), 121.7 (t, ^*1*^*J* = 241.5 Hz), 113.5, 62.2 (t, ^*4*^*J* = 3.2 Hz), 21.7, 19.5 (2C)	**CDCl**_**3**_**, 282** **MHz**: – 82.87 (s)
**17**	**(CDCl**_**3**_**, 500** **MHz)**: 7.44 (s, 1H), 7.42 (s, 1H), 7.36 (s, 1H), 7.23–7.29 (m, 2H), 7.21 (m, 1H), 3.57 (s, 3H), 2.9 (sext., 1H, *J* = 6.9 Hz), 2.31 (s, 3H), 2.30 (s, 3H), 1.23 (d, 6H, *J* = 6.9 Hz)	**(CDCl**_**3**_**, 125** **MHz)**:168.7, 148.8, 139.6, 138.7, 137.7 (t, ^*2*^*J* = 27.3 Hz), 132.6 (t, ^*2*^*J* = 27.3 Hz), 130.7, 128.8 (t, ^*3*^*J* = 3.4 Hz), 128.6 (t, ^*3*^*J* = 7.6 Hz), 128.0, 127.8 (t, ^*4*^*J* = 1.8 Hz), 123.9 (t, ^*3*^*J* = 5.0 Hz), 123.6 (t, ^*3*^*J* = 5.2 Hz), 120.7 (t, ^*1*^*J* = 241.2 Hz), 52.0, 34.1, 23.9 (2C), 19.9, 19.4	**(CDCl**_**3**_**, 470** **MHz)**: – 81.94 (s)
**18**	**CDCl**_**3**_**, 300** **MHz**: 7.25 (s, 1H), 7.23 (s, 1H), 7.16 (s, 1H), 7.14 (s, 1H), 7.12 (m, 1H), 7.05 (m, 1H), 4.39 (s, 2 H), 2.76 (sext., 1H, *J* = 6.9 Hz), 2.41 (br. s, 1H), 2.13 (s, 3H), 2.12 (s, 3H), 1.1 (d, 6H, *J* = 6.9 Hz)	**CDCl**_**3**_**, 75** **MHz**: 149.1138.9, 137.3 (t, ^*2*^*J* = 27.8 Hz), 136.4, 135.2, 131.1 (t, ^*2*^*J* = 26.5 Hz), 130.5, 128.3, 128.0 (t, ^*4*^*J* = 1.7 Hz), 127.7 (t, ^*3*^*J* = 7.8 Hz), 123.9 (t, ^*3*^*J* = 5.0 Hz), 123.6 (t, ^3^*J* = 5.2 Hz),121.7 (t, ^*1*^*J* = 240.8 Hz), 61.6 (t, ^*4*^*J* = 3.2 Hz), 33.9, 23.7 (2C), 19.3 (2C)	**CDCl**_**3**_**, 282** **MHz**: − 82.66 (s)

**Table 6:** The physicochemical properties of synthesized bisarylic derivatives **A1** to **A5**

Compound	IUPAC Name	Aspect	Mass of the starting material (mg)	Mass of the product	*Rf* value (PE:EtOAc)	m.p. (^o^C)	% yield
**A1**	2-(3-(2-benzoyl-4,5-dimethylbenzoyl)phenyl)propanoic acid	White solid	270	225	0.17 (5:5)	86–88	80
**A2**	2-(3-(difluoro(2-(methoxycarbonyl)-4,5-dimethylphenyl)methyl)phenyl)propanoic acid	White solid	74	60	0.40 (9.5:0.5)	98–100	78
**A3**	2-((3-bromophenyl)difluoromethyl)-4,5-dimethylbenzoic acid	White solid	100	83	0.28 (6:4)	64–66	80
**A4**	2-((3-acetylphenyl)difluoromethyl)-4,5-dimethylbenzoic acid	White solid	103	80	0.38 (8:2)	118–120	74
**A5**	2-(difluoro(3-isopropylphenyl)methyl)-4,5-dimethylbenzoic acid	White solid	100	55	0.26 (7:3)	152–154	53

**Table 7:** Spectral data of synthesized bisarylic derivatives **A1** to **A5**

Compound	^1^H NMR	^13^C NMR (CDCl_3_, 75 MHz)	^19^F NMR (CDCl_3_, 282 MHz)	HRMS (ESI)
**A1**	**CDCl**_**3**_**, 300** **MHz**: 7.78 (m, 2H), 7.53 (m, 2H), 7.36 (t, 1H, *J* = 7.7 Hz), 7.17 (s, 1H), 3.77 (quad, 1H, *J* = 7.2 Hz), 3.51 (s, 3H), 2.36 (s, 3H), 2.33 (s, 3H), 1.51 (d, 3H, *J *= 7.2 Hz)	**CDCl**_**3**_**, 75** **MHz**: 197.0, 179.7, 166.7, 141.9, 140.2, 138.9, 138.7, 137.9, 132.0, 131.0, 129.2, 128.7, 128.6, 128.1, 126.8, 51.9, 45.1, 19.9, 19.6, 18.0		calcd. For C_20_H_20_O_5_Na: m/z [M + Na]^+^ 363.12029; found: 363.1203 (0 ppm); C_20_H_19_O_5_Na_2_: m/z [M-H + 2Na]^+^ 385.10224; found: 385.1014 (2 ppm).
**A2**	**CDCl**_**3**_**, 300** **MHz**: 7.44–7.46 (m, 3H), 7.33–7.40 (m, 3H), 3.76 (quad, 1H, *J* = 7.1 Hz), 3.56 (s, 3H), 2.33 (s, 3H), 2.32 (s, 3H), 1.50 (d, 3H, *J *= 7.2 Hz)	**CDCl**_**3**_**, 75** **MHz**: 179.6, 168.5, 139.8, 139.7, 138.9, 138.3 (t, ^2^*J* = 27.7 Hz), 132.3 (t, ^*2*^*J* = 27.1 Hz), 130.8, 128.9 (t, ^*4*^*J* = 1.7 Hz), 128.7 (t, ^*3*^*J* = 3.4 Hz), 128.5 (2C), 125.2 (t, ^*3*^*J* = 5.1 Hz), 120.4 (t, ^*1*^*J* = 241.6 Hz), 68.3, 52.0, 45.2, 19.9, 19.4, 18.1	**CDCl**_**3**_**, 282** **MHz**: – 82.30 (s)	calcd. For C_20_H_20_F_2_O_4_Na: m/z [M + Na]^+^ 385.12219; found: 385.1223 (0 ppm); C_20_H_19_O_4_F_2_Na_2_: m/z [M + Na]^+^ 407.10413; found: 407.1045 (0 ppm).
**A3**	**deuterated acetone, 300** **MHz**: 7.29–7.63 (m, 6H), 2.22 (s, 3H), 2.20 (s, 3H)	**deuterated acetone, 75** **MHz:** 170.0, 142.4 (t, ^2^*J* = 28.6 Hz), 141.7, 141.1 (t, ^*4*^*J* = 1.3 Hz), 134.6 (t, ^*3*^*J* = 1.7 Hz), 133.7 (t, ^2^*J* = 26.9 Hz), 132.7, 132.1, 131.3, 130.7 (t, ^*3*^*J* = 5.3 Hz), 130.0 (t, ^3^*J* = 8.0 Hz), 126.8 (t,^*3*^*J* = 5.3 Hz), 123.4, 122.0 (t, ^*1*^*J* = 241.6 Hz), 20.8, 20.4	**CDCl**_**3**_**, 282** **MHz**: − 82.34 (s)	calcd. For C_16_H_13_O_2_F_2_^79^BrNa: m/z [M + Na]^+^ 376.99592; found: 376.9958 (0 ppm); C_16_H_12_O_2_F_2_^79^BrNa_2_: m/z [M-H + 2Na]^+^ 398.97786; found: 398.9779 (0 ppm); C_16_H_12_O_2_F^79^BrNa: m/z [M-HF + Na]^+^ 356.98969; found: 356.9905 (2 ppm)
**A4**	**deuterated acetone, 300** **MHz**: 8.18 (m, 1H), 8.07 (m, 1H), 7.77 (m, 1H), 7.56–7.61 (m, 3H), 2.60 (s, 3H), 2.39 (s, 3H), 2.36 (s, 3H)	**deuterated acetone, 75** **MHz**: 198.5, 170.0, 141.7, 141.1, 140.6 (t, ^*2*^*J* = 28.5 Hz), 138.9, 133.9 (t, ^*2*^*J* = 27.1 Hz), 132.6, 132.1 (t,^*3*^*J* = 7.5 Hz), 131.4, 131.1 (t, ^*4*^*J* = 3.4 Hz), 130.4, 130.0 (t, ^*3*^*J* = 7.9 Hz), 127.2 (t, ^*3*^*J* = 5.3 Hz), 122.5 (t, ^*1*^*J* = 241.7 Hz), 27.7, 20.8, 20.3	**CDCl**_**3**_**, 282** **MHz**: – 82.87 (s)	calcd. For C_18_H_16_O_3_F_2_Na: m/z [M + Na]^+^ 341.09597; found: 341.0960 (0 ppm)
**A5**	**CDCl**_**3**_**, 300** **MHz**: 7.47 (s, 1H), 7.43 (s, 1H), 7.30 (s, 1H), 7.15–7.17 (m, 3H), 2.81 (sext., 1H, *J* = 6.9 Hz), 2.27 (s, 3H), 2.24 (s, 3H), 1.14 (d, 6H, *J* = 6.9 Hz)	**CDCl**_**3**_**, 75** **MHz**: 172.7, 148.7, 140.5, 138.6, 137.6 (t, ^*2*^*J* = 27.2 Hz), 133.6 (t, ^*2*^*J* = 27.7 Hz), 131.4, 128.8 (t, ^*3*^*J* = 7.8 Hz), 127.9 (2C), 127.7 (t, ^*4*^*J* = 1.6 Hz), 124.1 (t, ^*3*^*J* = 5.0 Hz), 123.6 (t, ^*3*^*J* = 5.2 Hz), 120.6 (t, ^*1*^*J* = 241.7 Hz), 34.1, 23.8 (2C), 19.9, 19.4	**CDCl**_**3**_**, 282** **MHz**: –81.93 (s)	calcd. For C_19_H_20_O_2_F_2_Na: m/z [M + Na]^+^ 341.13236; found: 341.1321 (1 ppm); C_19_H_19_O_2_FNa: m/z [M-HF + Na]^+^ 321.12613; found: 321.1260 (0 ppm); C_19_H_19_O_2_F_2_Na_2_: m/z [M-H + 2Na]^+^ 363.1143; found: 363.1157 (4 ppm); C_19_H_20_O_2_F_2_K: m/z [M + K]^+^ 357.10629; found: 357.1059 (1 ppm); C_19_H_19_O_2_: m/z [M-HF-F]^+^ 279.13796; found: 279.1379 (0 ppm)

### Synthesis of *gem*-difluorobisarylic derivatives A1, A2, A3, A4 and A5

To alcohol in acetone was added dropwise under magnetic stirring at room temperature, a concentrated (5.4 M) solution of Jones reagent until disappearance of the starting material (TLC analysis). After addition of isopropanol (5 equiv.), the reaction mixture was filtered, and the filtrate was extracted with ethyl acetate. The combined organic phases were dried over Na_2_SO_4_, filtered and concentrated in vacuum. After purification by chromatography on silica gel, carboxylic acid was obtained.

### Supporting information

Experimental details and characterization data of new compounds with copies of ^1^H, ^13^C and ^19^F NMR spectra are presented in the supplementary section, Additional file 1.

#### Evaluation of inflammation in macrophages

C57BL/6J male mice (20–25 g, 8 week-old) were obtained from Charles River (Ecully, France) and the animal facility of the American University of Beirut. Mice were housed 5 per cage in temperature- and humidity-controlled rooms, kept on a 12-h light–dark cycle, and provided with standard food and water ad lib and with enrichment environment (cotton cocoon) in the animal facility of the American University of Beirut. Body weight and food intake were monitored three times a week throughout the study period. ARRIVE guidelines were followed (Additional file 2). Approval for use of animals was obtained from the Institutional Animal Care and Use Committee of the American University of Beirut (IACUC # 16-09-m379).

On the day of the procedure, 2–3 mice were euthanatized after 3 min exposure to carbon dioxide. BMDM were isolated as previously described and were plated at 0.8 million cells per well [16]. Flow cytometry analysis was performed using F4/80 -APC antibody (BioLegend 123115) and showed 90% macrophages. BMDM were then treated for 24 h with different concentrations of the bisarylic derivative compounds for 30 min prior to the addition of 10 ng/mL LPS. DMSO concentration did not exceed 0.4% with no effect. The supernatants were assessed for IL-6, PGE_2_ and nitrite, the stable derivative of NO. Cells were washed with PBS and lysed in RIPA buffer containing inhibitors of protease. Total protein concentration was determined using DC protein assay (Bio-Rad 500-0115) with BSA as standard. IL-6, nitrite and PGE_2_ were measured as described previously [17]. Western blot of NOS-II and COX-2 was performed as previously described [17–19]. 10 µg of total protein was assessed. The primary antibodies were developed and characterized as previously described: for COX-2, mouse monoclonal antibody anti-COX-2 (clone COX-214, 1/5000) [20]; for NOS-II, rabbit polyclonal antibody anti-NOS-II (dilution1/2000) [21], and mouse β-actin (dilution 1/10,000) (Sigma-Aldrich A5441). Clarity™ western ECL substrate (Bio-Rad 170-5061) was used according to the manufacturer’s instructions to reveal positive bands visualized using Bio-Rad ChemiDoc.

#### COX-1 and COX-2 activities

For COX-1 activity, human embryonic kidney (HEK)-293 cells (ATCC CRL-1573, Manassas, VA USA) stably overexpressing human recombinant COX-1 were used [17]. Cells were treated with compounds **A1** to **A5** for 45 min in Hanks buffer and then 10 µM arachidonic acid (AA) were added for 30 min. PGE_2_ was measured and corresponded to the breakdown metabolism of PGH_2_ and PGE_2_.

For COX-2 activity, BMDM were treated with 10 μM acetylsalicylic acid (ASA) for 30 min to irreversibly inhibit COX-1 and then washed and treated with LPS 10 ng/mL for 24 h. BMDM were treated with compounds **A1** to **A5** for 45 min in Hanks buffer, pH 7.4 containing 1 mg/mL BSA prior to the addition of 10 µM of AA for 30 min. Supernatants were collected and PGE_2_ was determined.

#### Toxicity assay

WST-1 assay was used to determine the toxicity of the synthesized compounds (Cell proliferation WST-1 assay, Sigma-Aldrich 5015944001). Briefly, macrophages (50,000 cells per well) were plated in a 96 well plate in RPMI culture medium containing 10% FBS and grown for 24 h. Cells (in triplicates) were treated at 25 and 50 μM of the tested compounds. Culture medium without cells and cells without treatment were used as control. Results were expressed as percentage of cells without treatment. All compounds showed 95% viability at 50 μM.

### Molecular docking

#### Target and small molecule preparation

All small molecules (**A1** to **A5**) were built using Openbabel chemical toolbox (PMID: 21982300) and subsequent low energy 3D conformations were generated using Frog2 (PMID: 20444874). Protonation state corresponded to a pH of 7. The 3D structure of the ovine COX-1 (PDBID: 1EQG) [13] and murine COX-2 (4ph9) [14] complexed with ibuprofen were selected for docking simulations using Autodock Vina (PMID: 19499576) optimized for virtual screening. The numbering of the amino acid residues in the PDB is different between ovine COX-1 and murine COX-2, i.e. Arg120 in COX-1 corresponds to Arg121 in murine COX-2. Water and other heteroatoms were removed from the structure. Chain A was retained including ibuprofen and heme group. Hydrogen atoms were added, atom typing, and partial charges were assigned using Amber forcefield in Chimera (PMID: 15264254). Corresponding ligand-receptor binding energies were estimated in kcal/mol and averaged for best poses that recapitulate ibuprofen binding. A single interacting conformation was retained after visual inspection in Maestro (Schrödinger, LLC).

#### Data analysis

IL-6, nitrite and PGE_2_ measurement were determined from 3 to 5 independent experiments and expressed as percentage of LPS alone and expressed as mean ± SEM. COX-1 activity for the compounds were expressed as percentage of PGE_2_ measured in cells exposed to vehicle and AA, and as percentage of PGE_2_ in cells treated with LPS, ASA and AA for COX-2. Curve fitting and calculation of the IC_50_ values were done using Grafit7 Software (Erithacus software, Staines, UK) and GraphPad Prism 6. Images of western blot were analyzed using ImageJ Software (version 1.52a, NIH, MA). Ratio of COX-2 to β-actin was determined and the results are expressed as fold of LPS signal.

Statistical analysis was performed using one-way ANOVA, followed by Dunnett’s multiple comparisons test. Differences were considered significant when p < 0.05 (GraphPad Prism 6 Software, La Jolla, CA, USA).

## Supplementary information

**Additional file 1.** Proton (H), carbon (C) and fluorine (F) NMR spectra for intermediates 4 to 18 and for compounds A1 to A5.

**Additional file 2.** ARRIVE Check list.

## Data Availability

The data used to support the findings of this study are available from the corresponding author upon request.

## Abbreviations

AA: arachidonic acid
ASA: acetylsalicylic acid
BMDM: bone marrow-derived macrophages
COX: cyclooxygenase
FBS: fetal bovine serum
IL-6: interleukin-6
LPS: lipopolysaccharide
NO: nitric oxide
NOS-II: nitric oxide synthase-II
NSAIDs: non-steroidal anti-inflammatory drugs
PGE_2_: prostaglandin E_2_
