# Supplementary material for: gem-Difluorobisarylic derivatives: design, synthesis and anti-inflammatory effect
Source: BMC Chem. 2019 Oct 31;13(1):124. doi: 10.1186/s13065-019-0640-5 (PMC6824041; doi:10.1186/s13065-019-0640-5)
Supplement: Supplementary file 1 — Additional file 1. Proton (H), carbon (C) and fluorine (F) NMR spectra for intermediates 4 to 18 and for compounds A1 to A5. [file 13065_2019_640_MOESM1_ESM.pdf]

Additional File 1: Proton (H), carbon (C) and fluorine (F) NMR spectra for intermediates **4** to **18** and for compounds **A1** to **A5**.

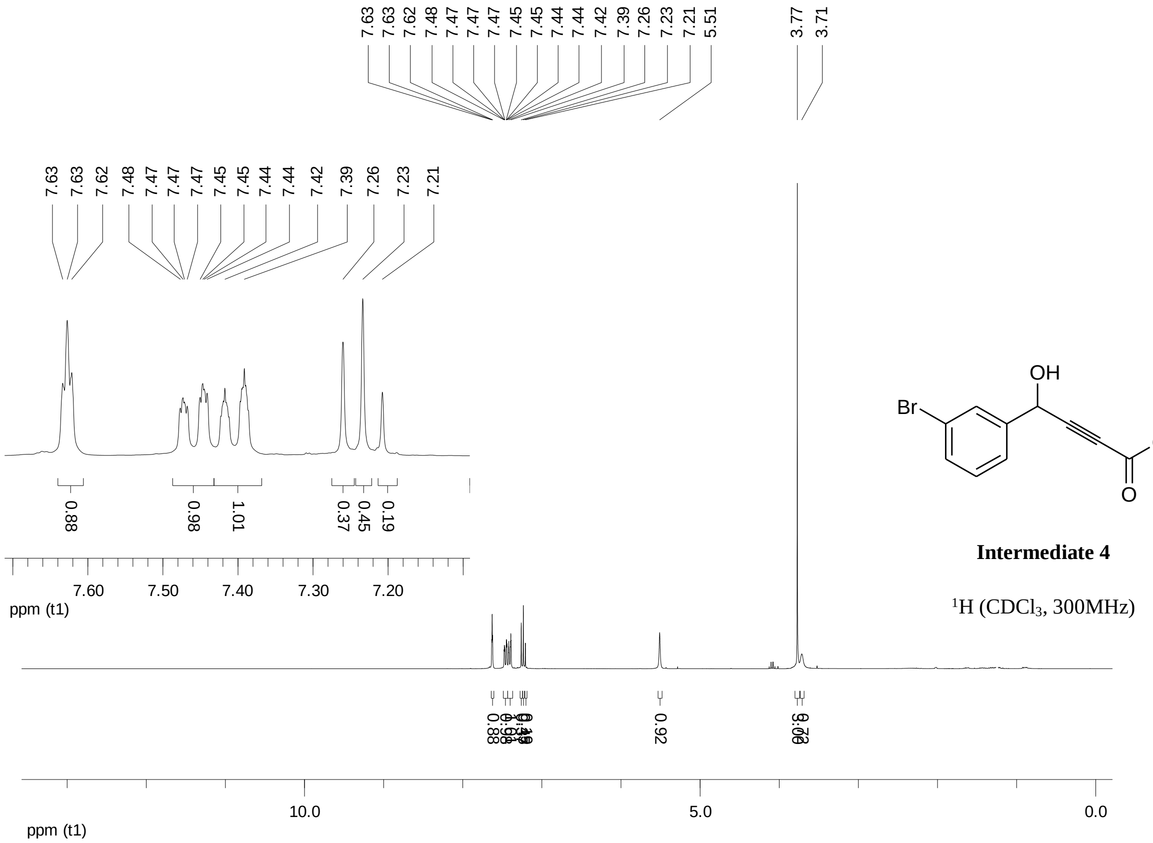

7.63 7.63 7.62 7.48 7.47 7.47 7.47 7.45 7.45 7.44 7.44 7.42 7.39 7.26 7.23 7.21 5.51
3.77 3.71
0.88 0.98 1.01 0.37 0.45 0.19
7.60 7.50 7.40 7.30 7.20
ppm (t1)
OH
Br
O
O
Intermediate 4
1H (CDCl3, 300MHz)
0.92
10.0 5.0 0.0
ppm (t1)

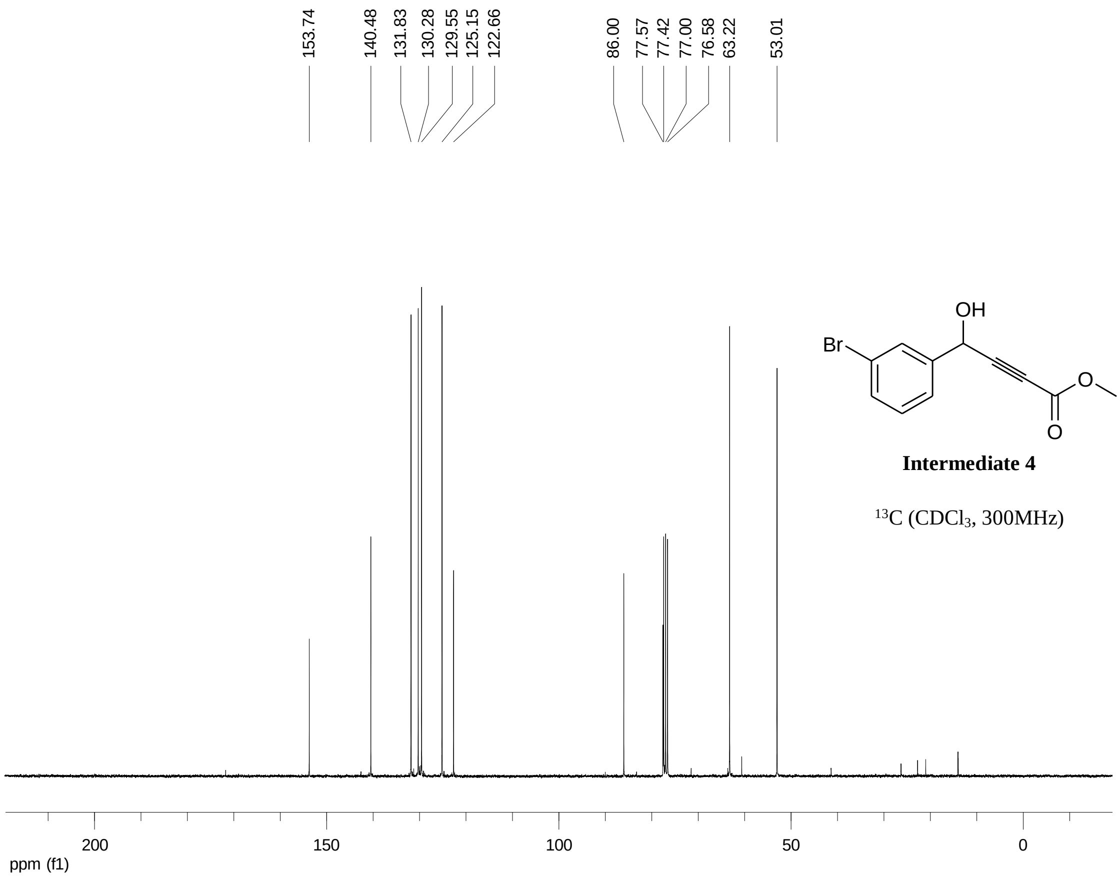

153.74 140.48 131.83 130.28 129.55 125.15 122.66
86.00 77.57 77.42 77.00 76.58 63.22 53.01
OH
Br
O
O
Intermediate 4
13C (CDCl3, 300MHz)
200 150 100 50 0
ppm (f1)

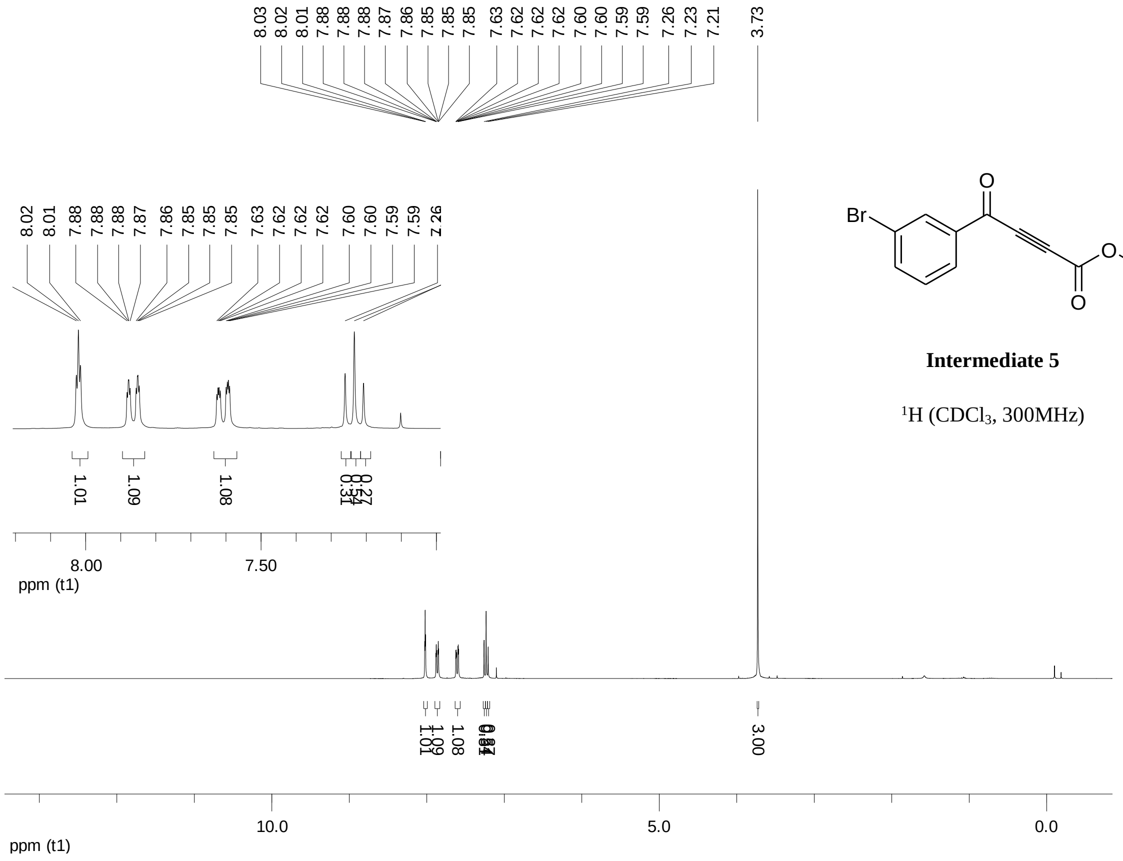

Intermediate 5

^{1}H ($CDCl_3$, 300MHz)

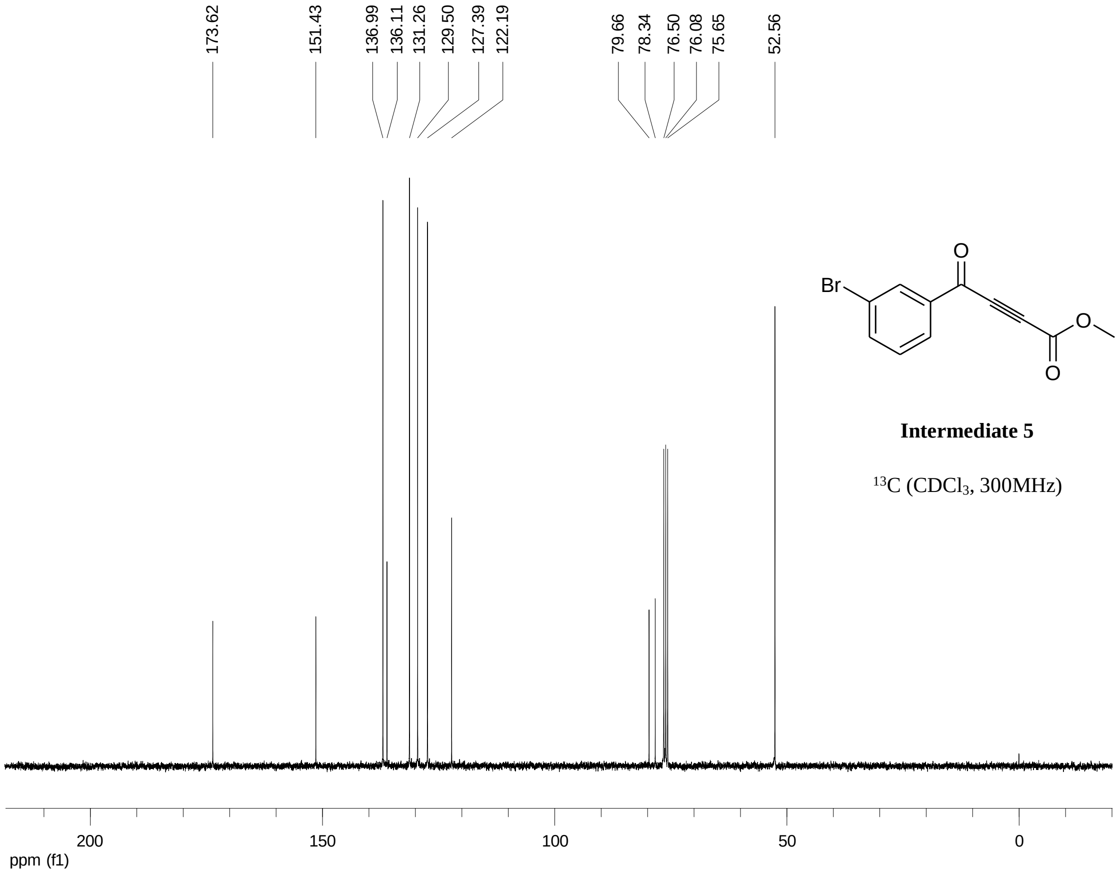

Intermediate 5

^{13}C ($CDCl_3$, 300MHz)

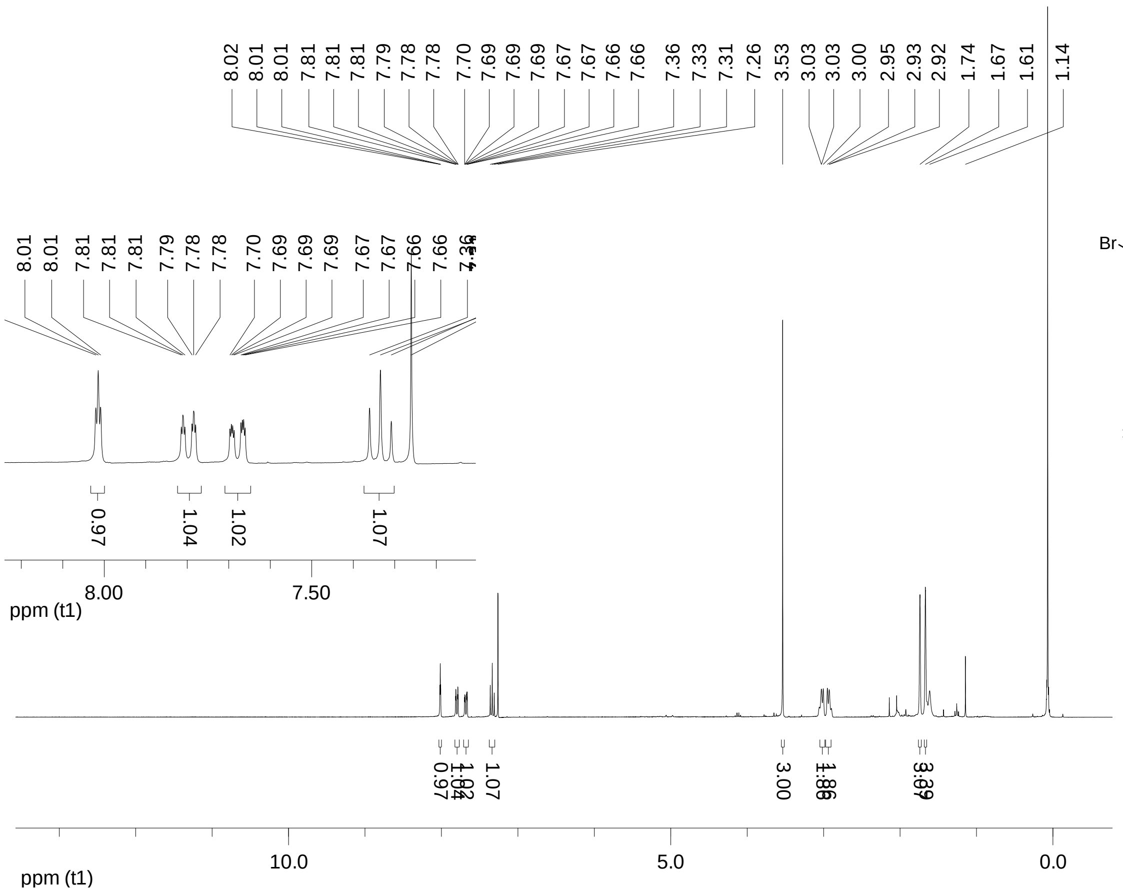

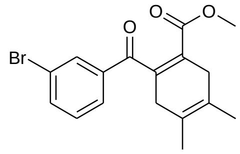

Intermediate 6

^{1}H ($CDCl_3$, 300MHz)

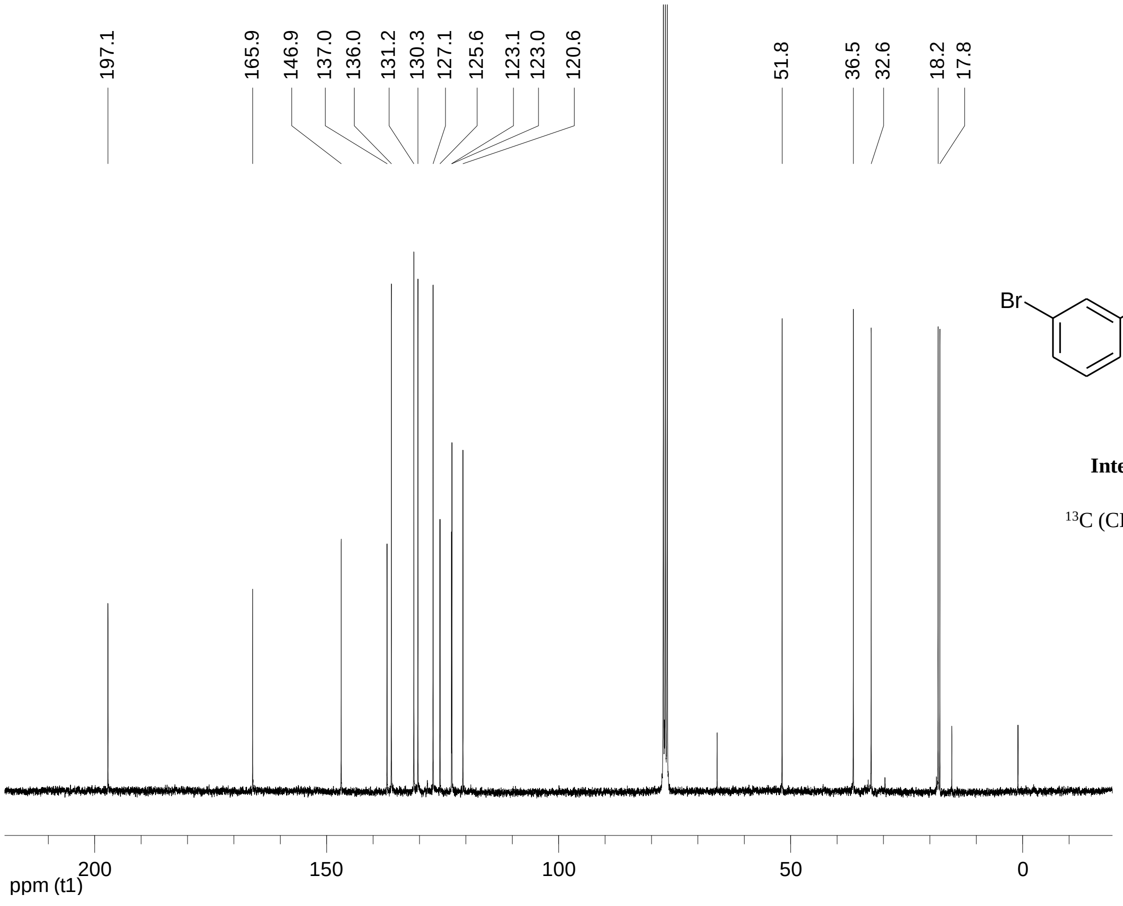

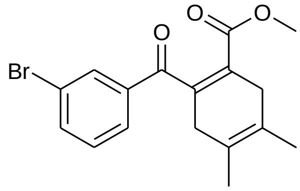

Intermediate 6

^{13}C ($CDCl_3$, 75 MHz)

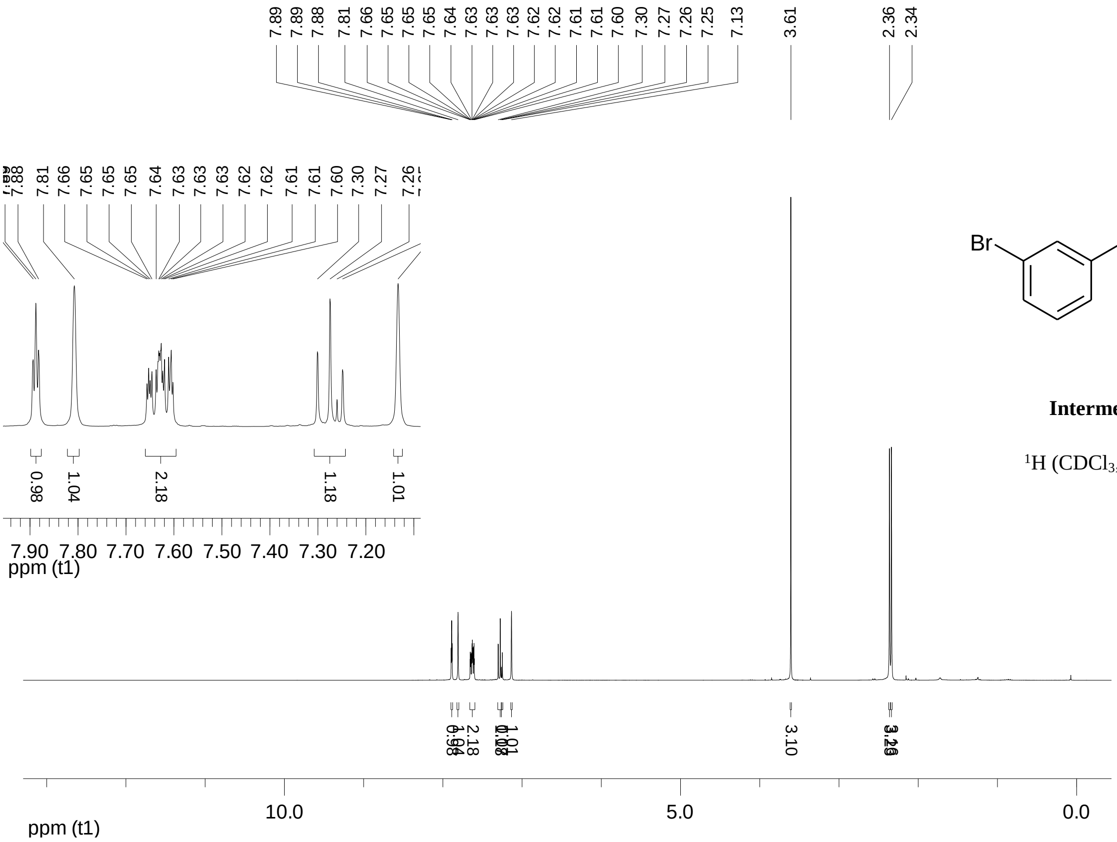

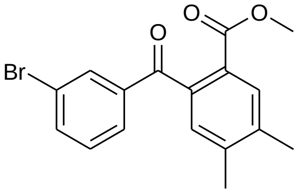

Intermediate 7

^{1}H ($CDCl_3$, 300MHz)

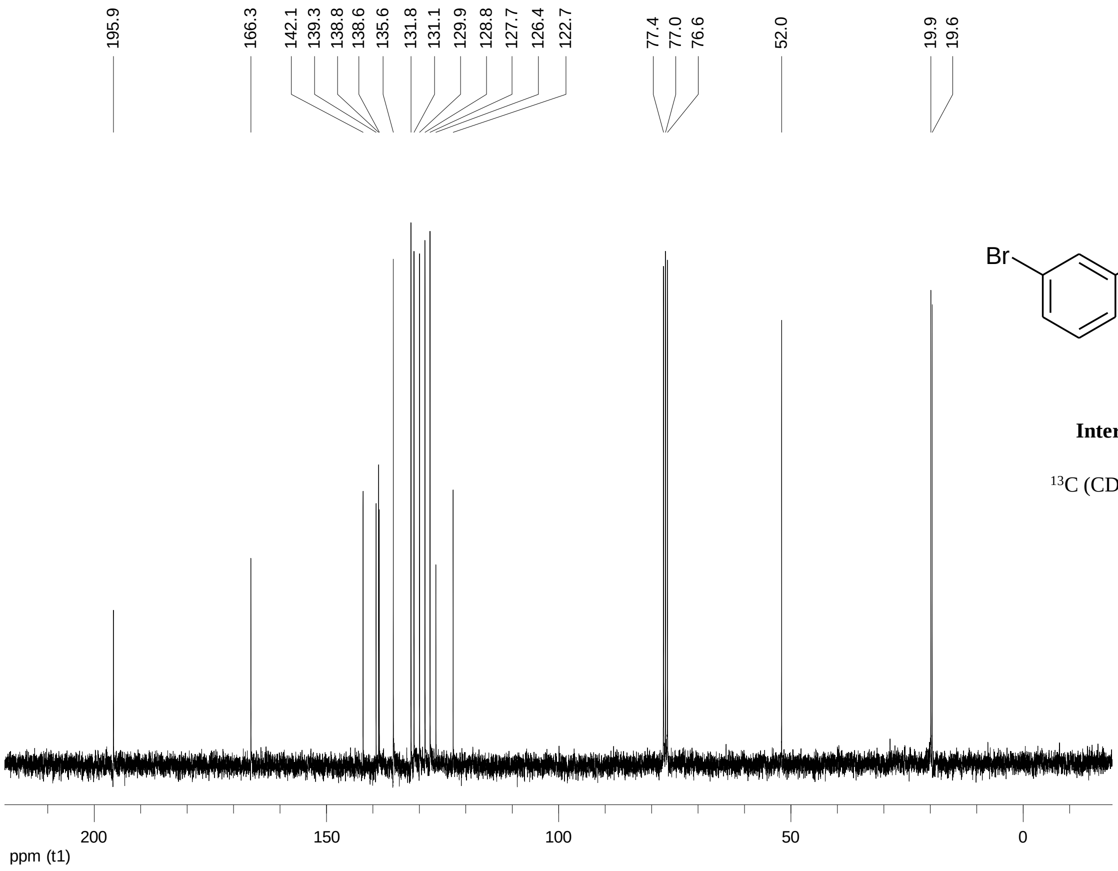

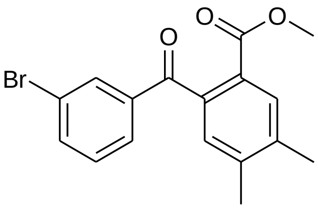

Intermediate 7

^{13}C ($CDCl_3$, 75 MHz)

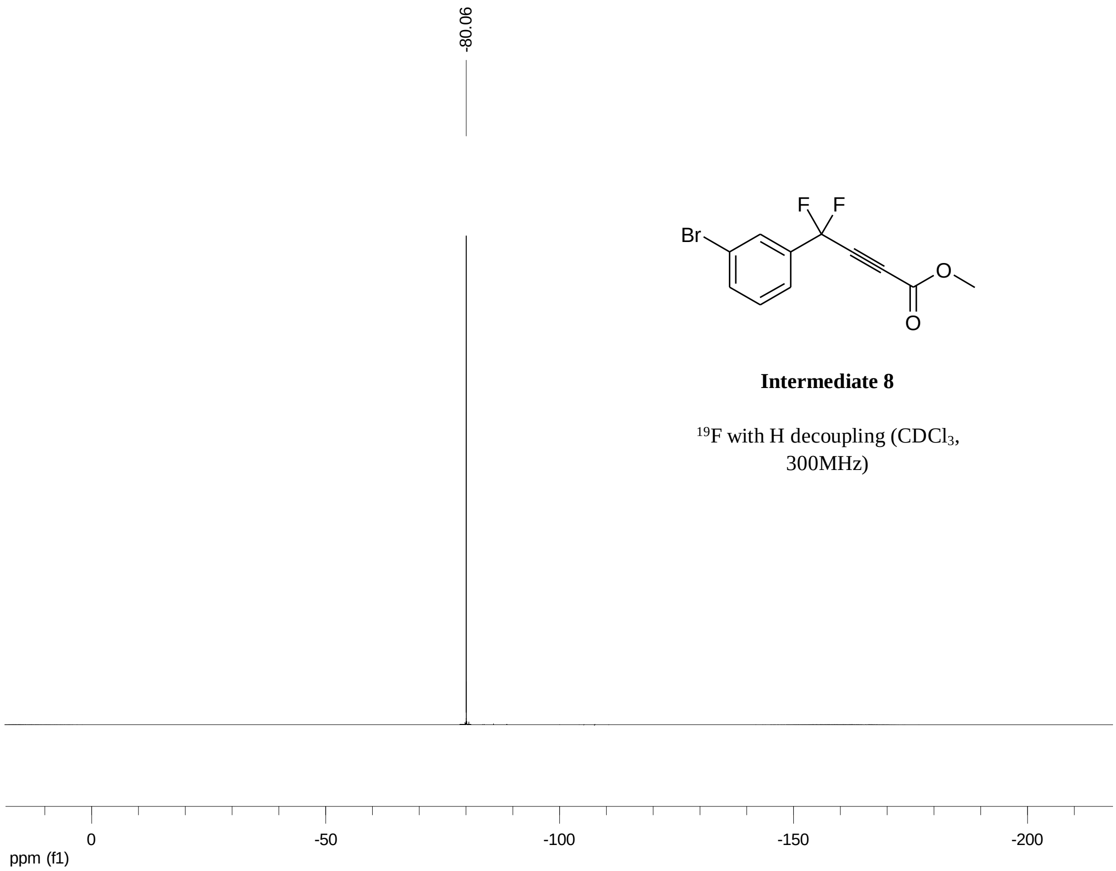

Intermediate 8

^{19}F with H decoupling ($CDCl_3$, 300MHz)

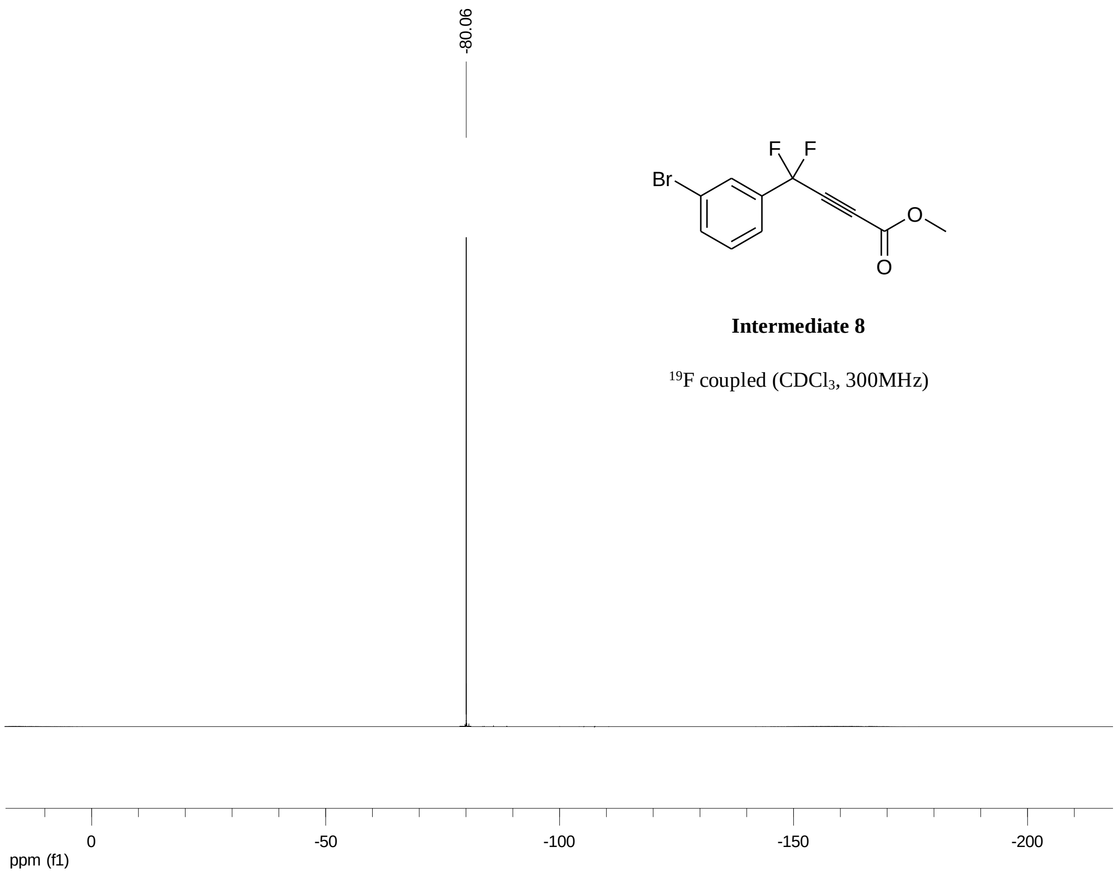

Intermediate 8

^{19}F coupled ($CDCl_3$, 300MHz)

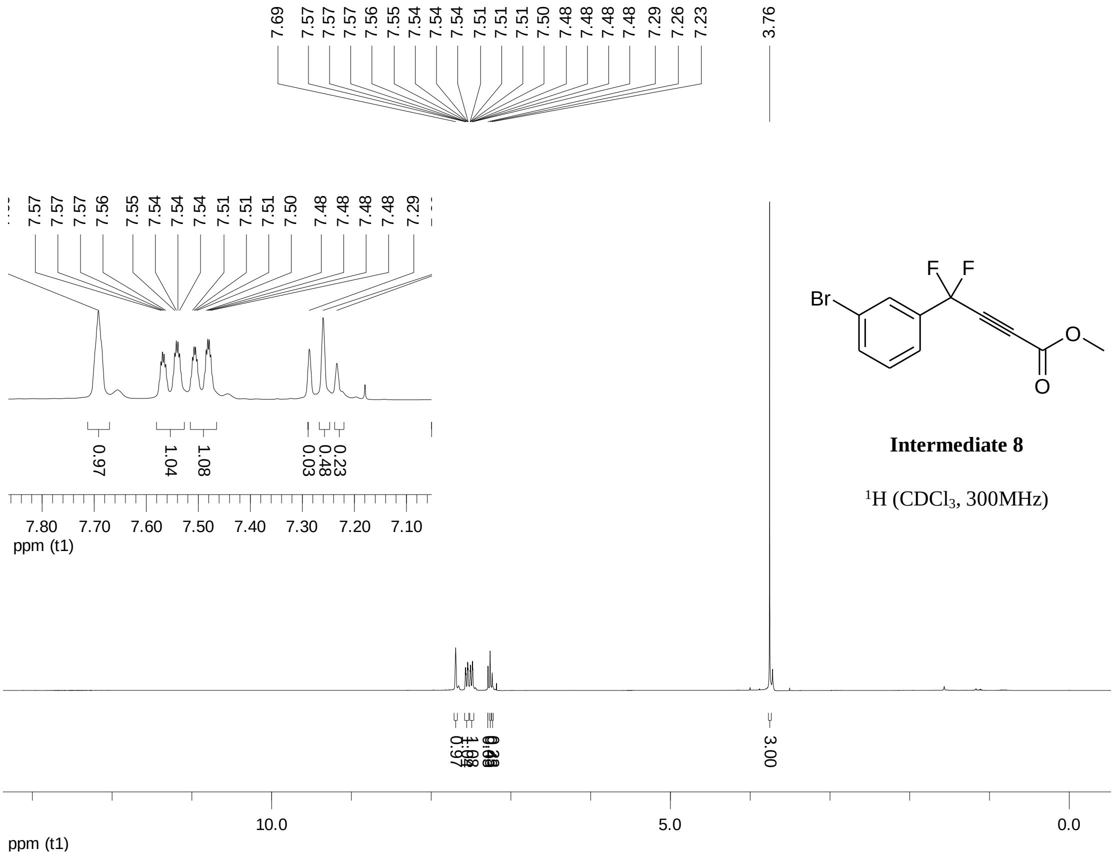

Intermediate 8

^{1}H ($CDCl_3$, 300MHz)

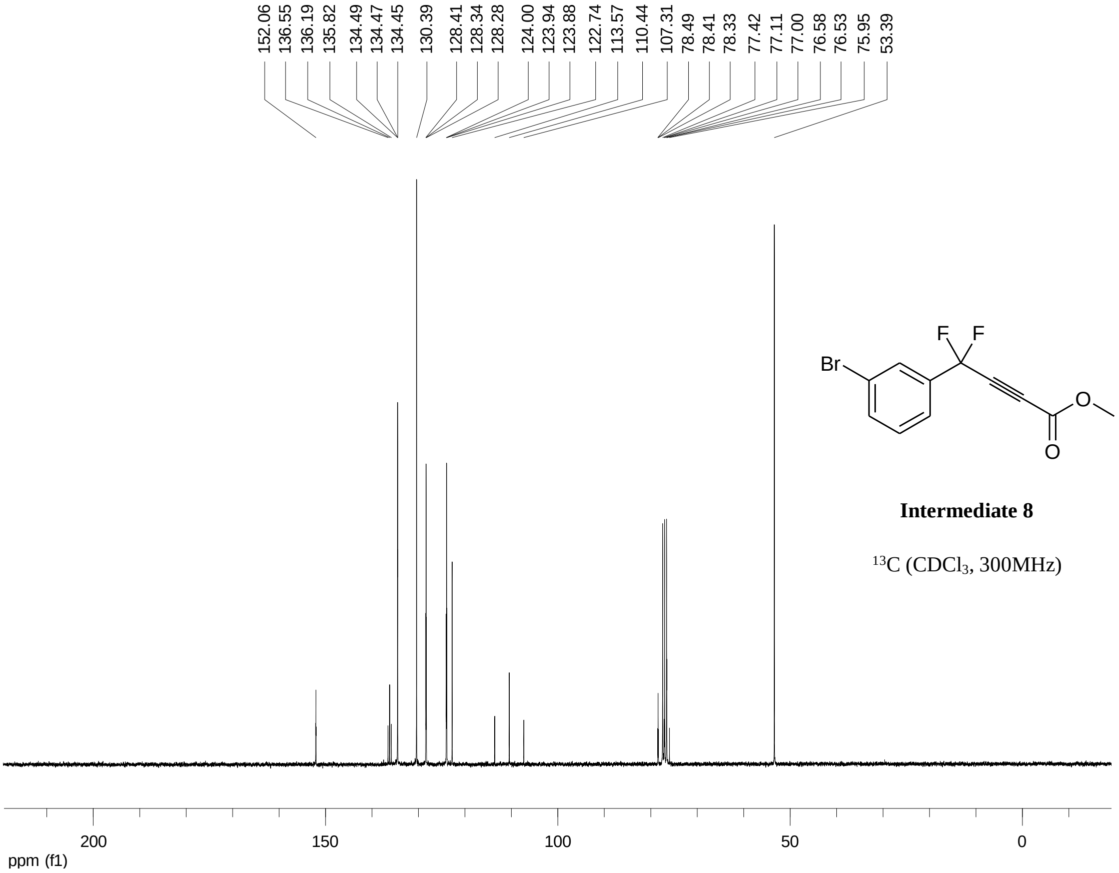

Intermediate 8

^{13}C ($CDCl_3$, 300MHz)

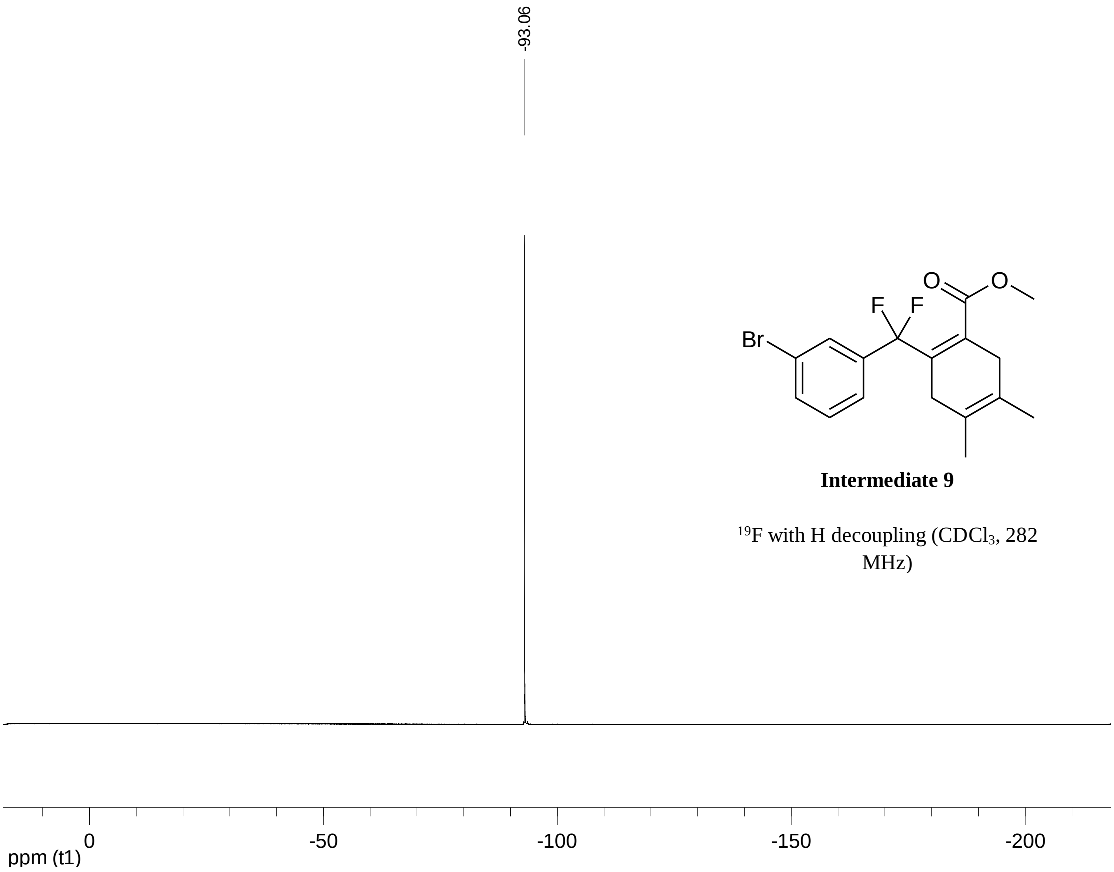

Intermediate 9

^{19}F with H decoupling ($CDCl_3$, 282 MHz)

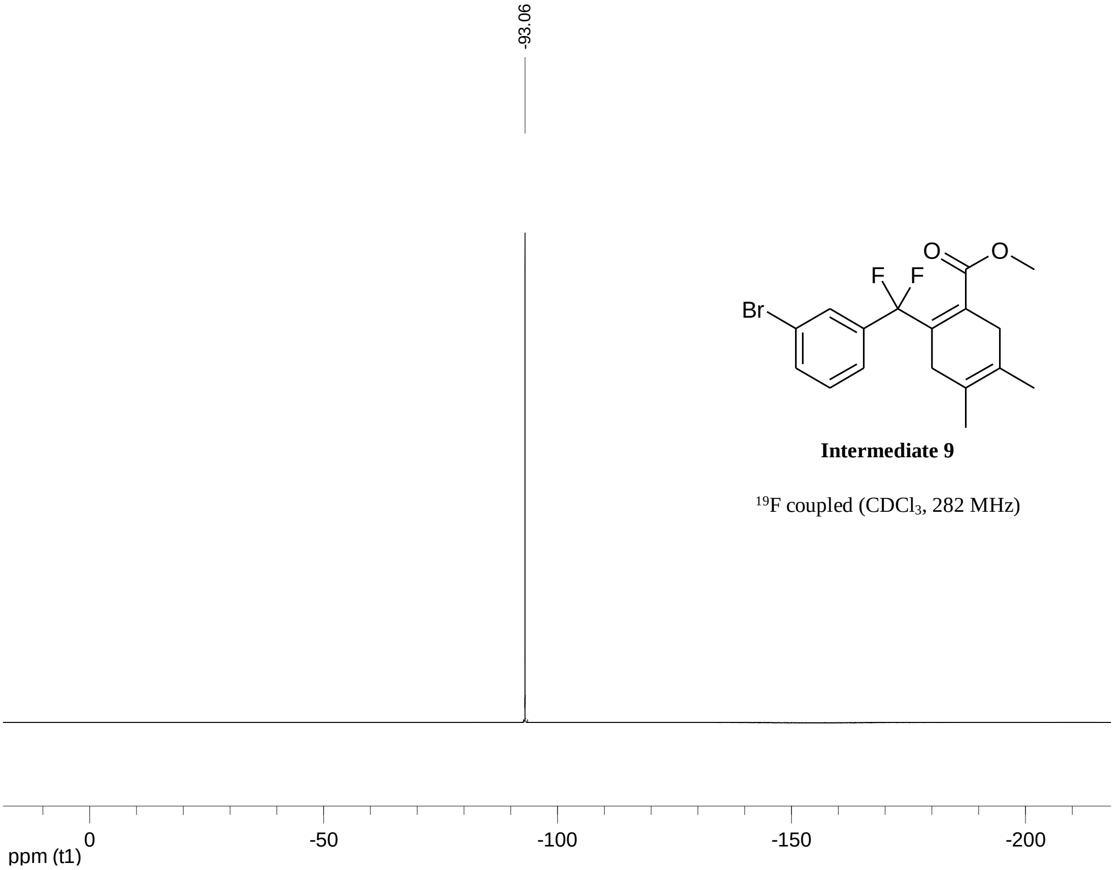

Intermediate 9

^{19}F coupled ($CDCl_3$, 282 MHz)

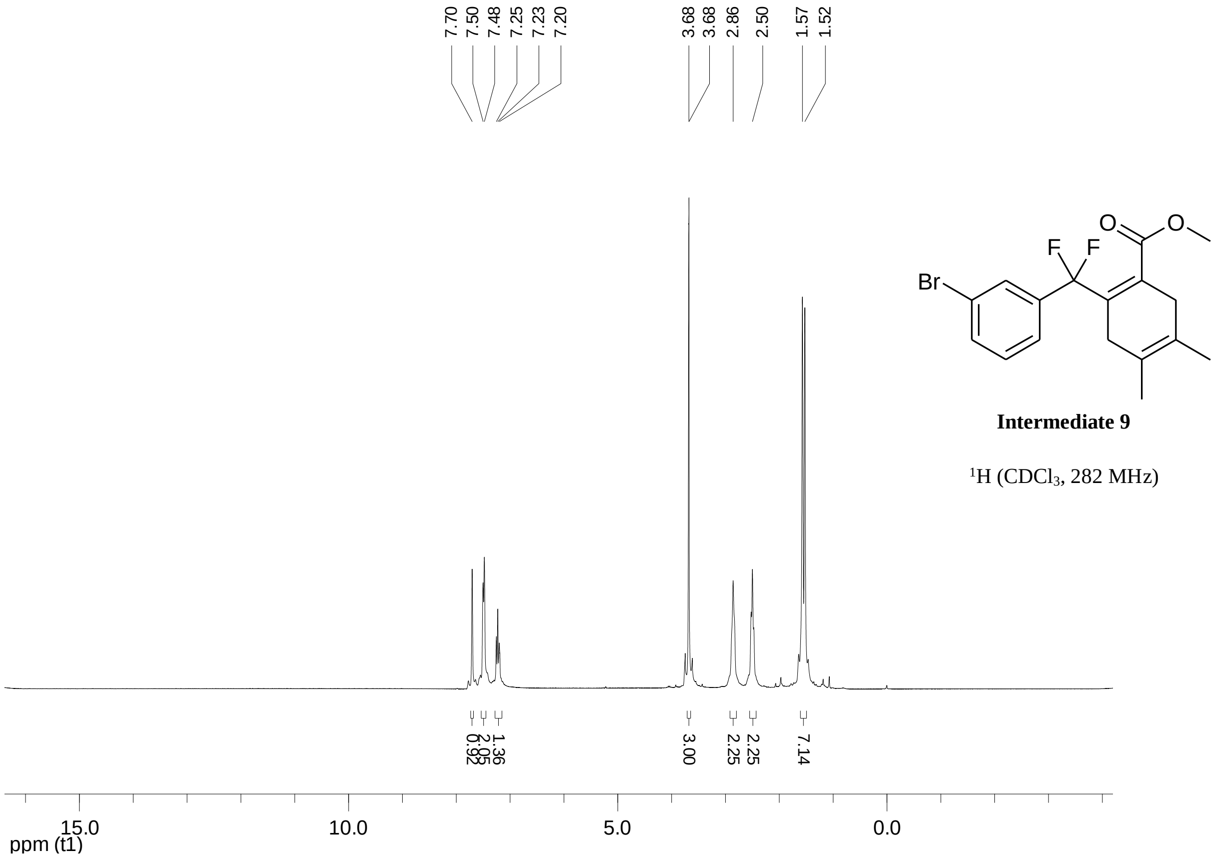

Intermediate 9

^{1}H ($CDCl_3$, 282 MHz)

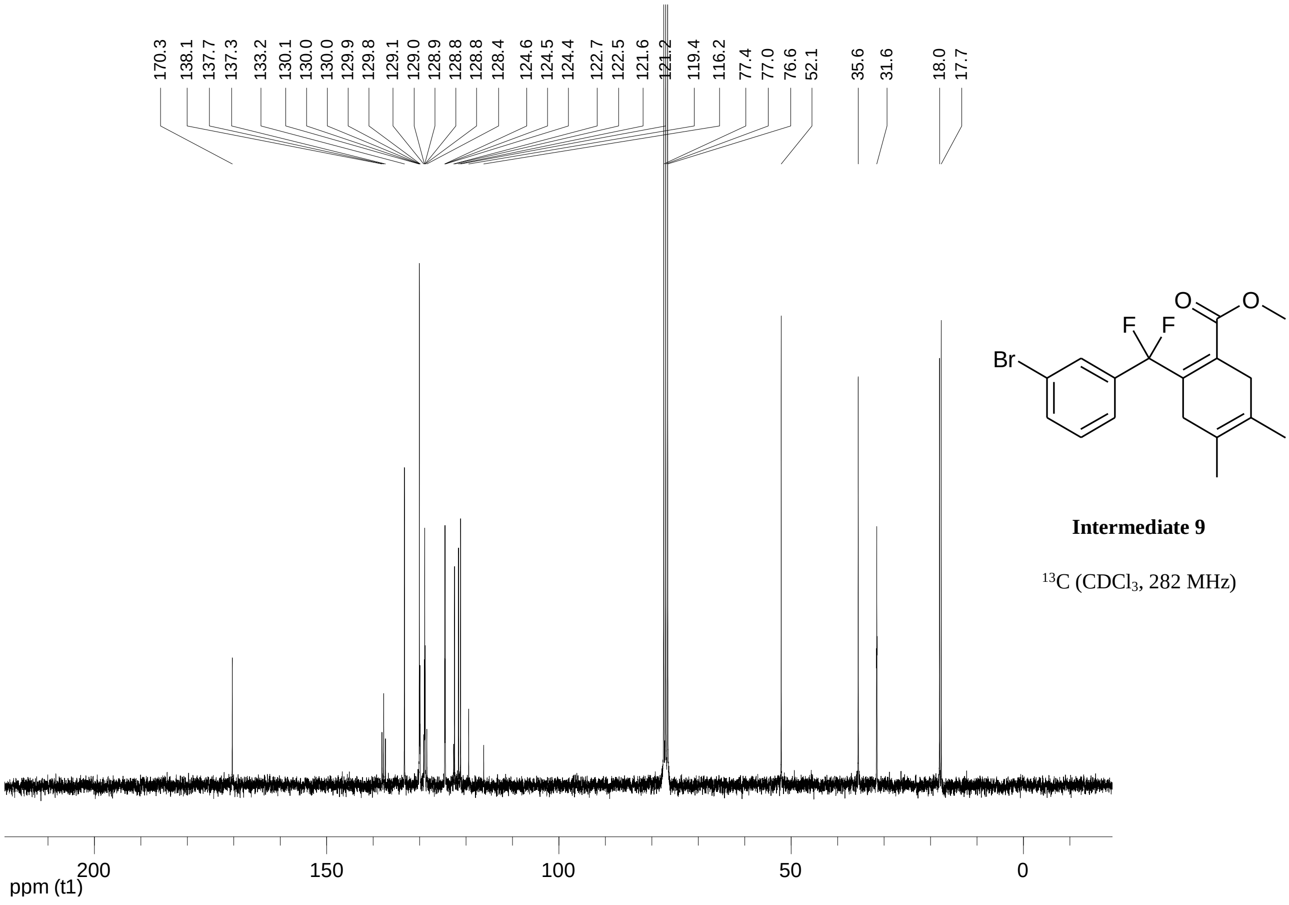

Intermediate 9

^{13}C ($CDCl_3$, 282 MHz)

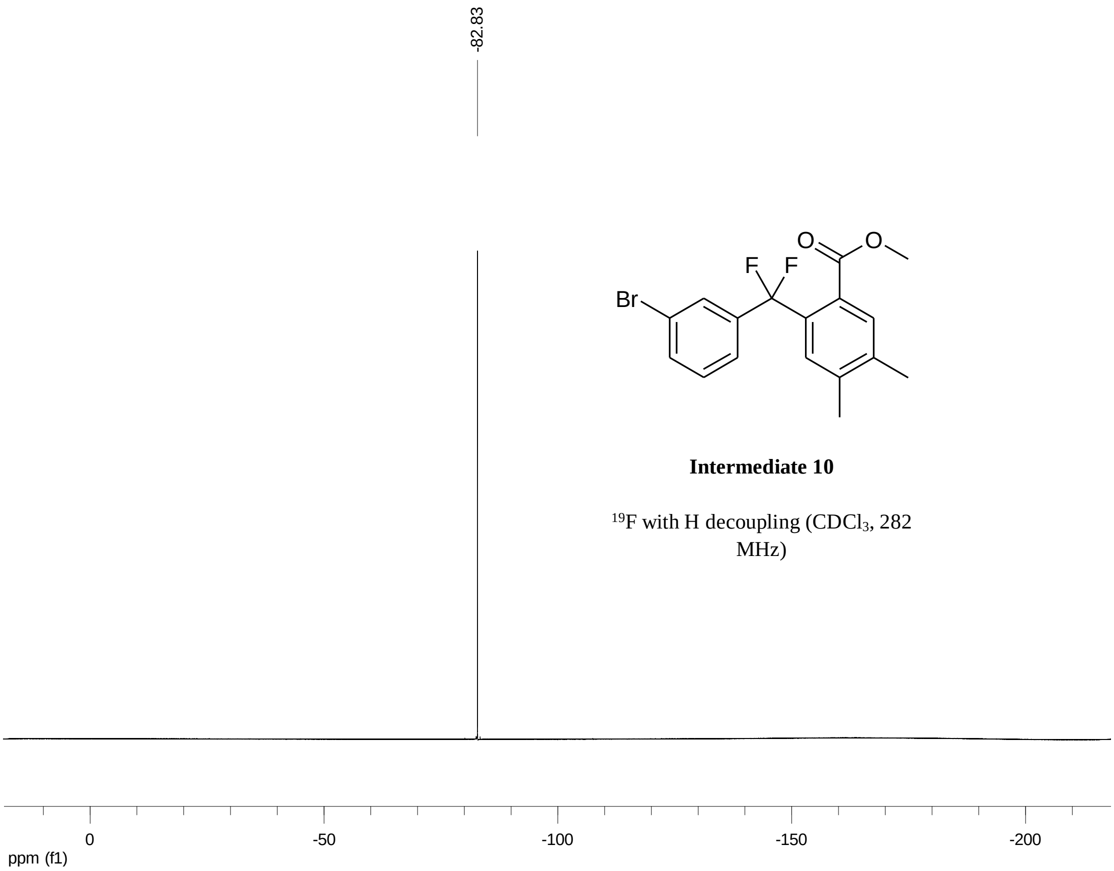

Intermediate 10

^{19}F with H decoupling ($CDCl_3$, 282 MHz)

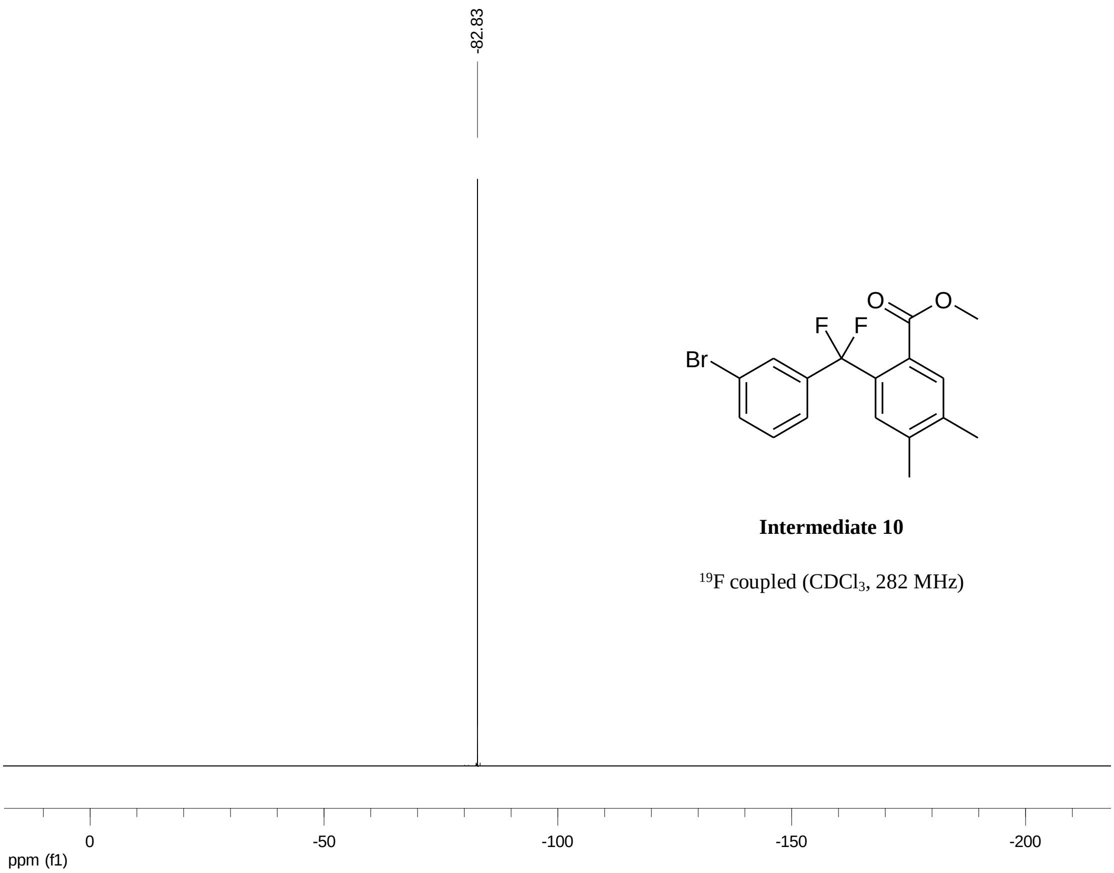

Intermediate 10

^{19}F coupled ($CDCl_3$, 282 MHz)

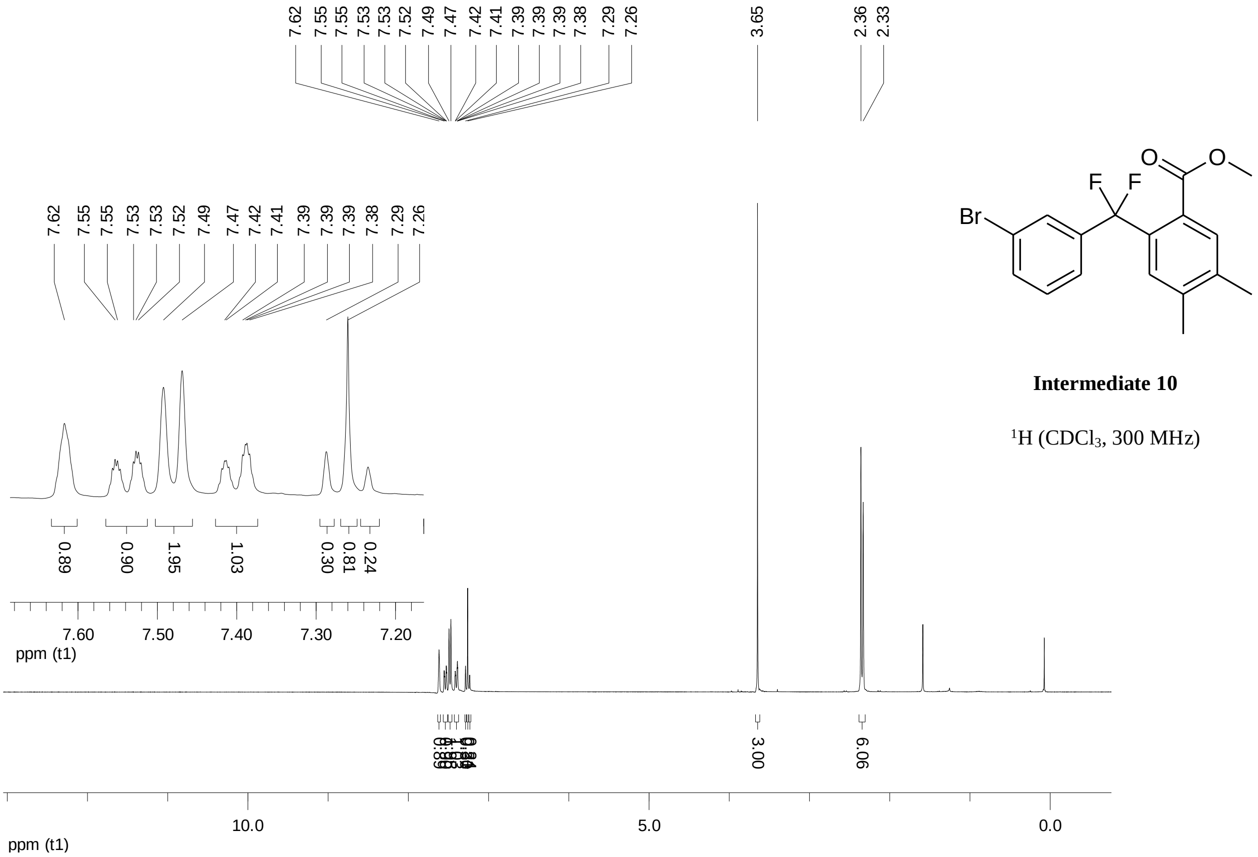

Intermediate 10

^{1}H ($CDCl_3$, 300 MHz)

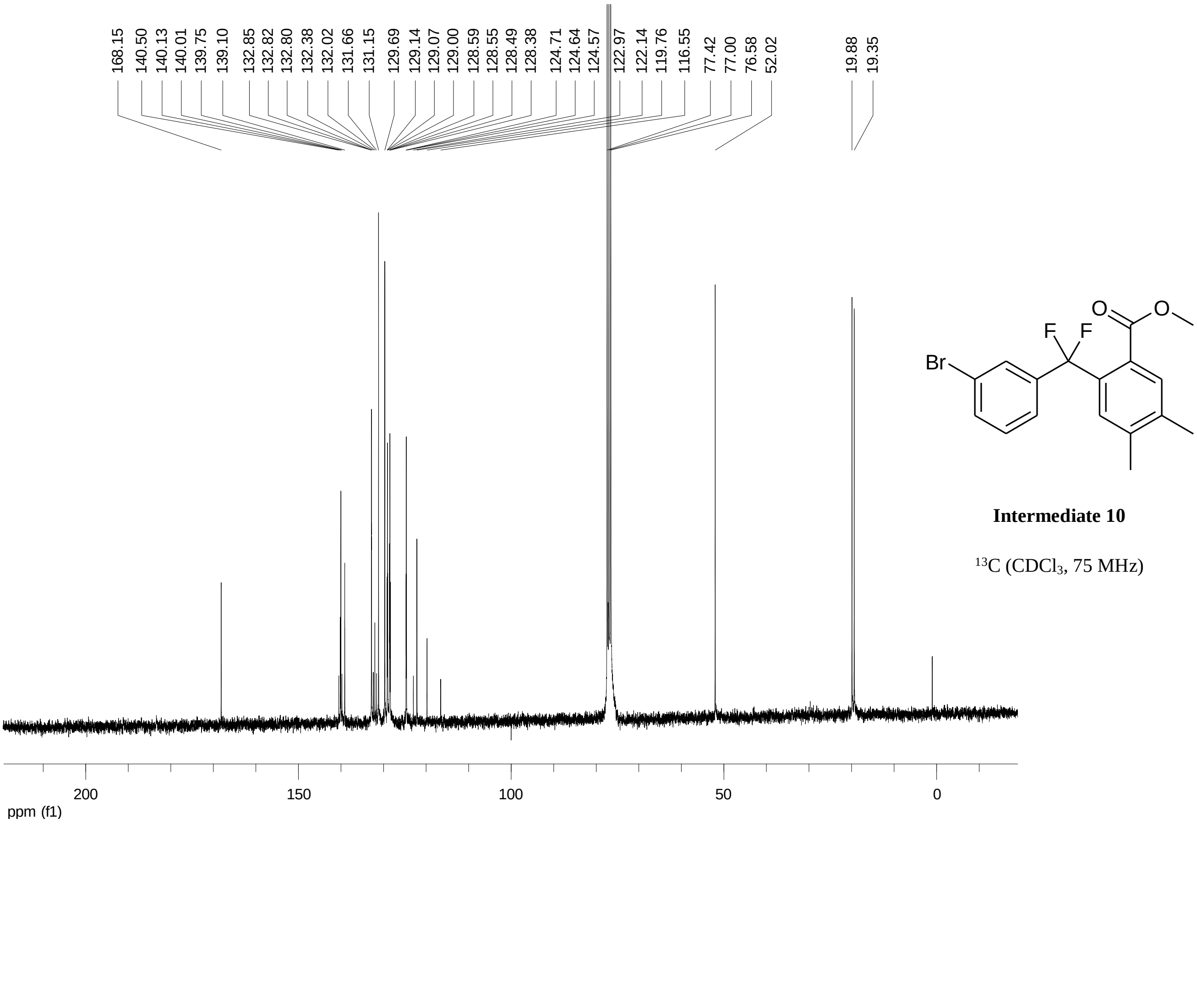

Intermediate 10

^{13}C ($CDCl_3$, 75 MHz)

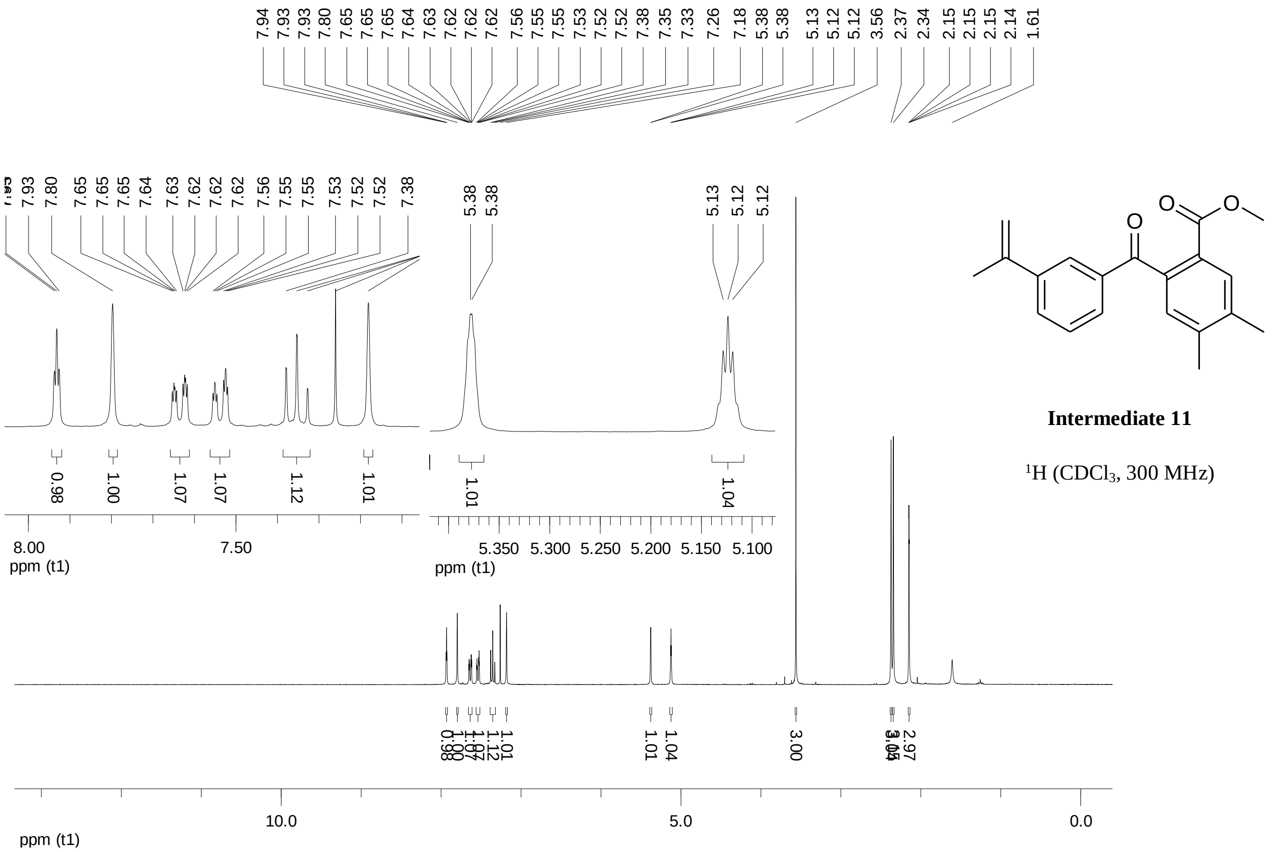

Intermediate 11

^{1}H ($CDCl_3$, 300 MHz)

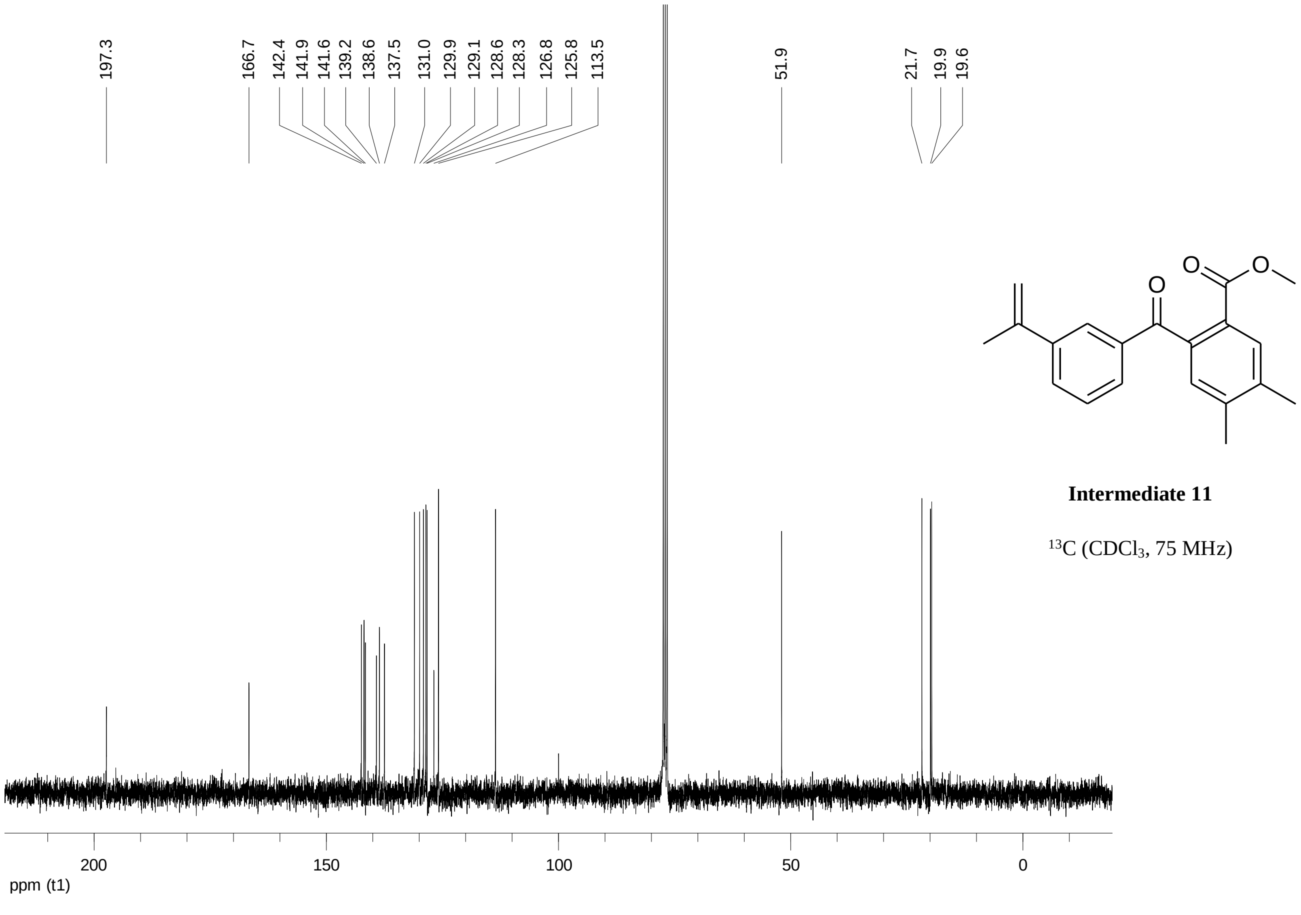

Intermediate 11

^{13}C ($CDCl_3$, 75 MHz)

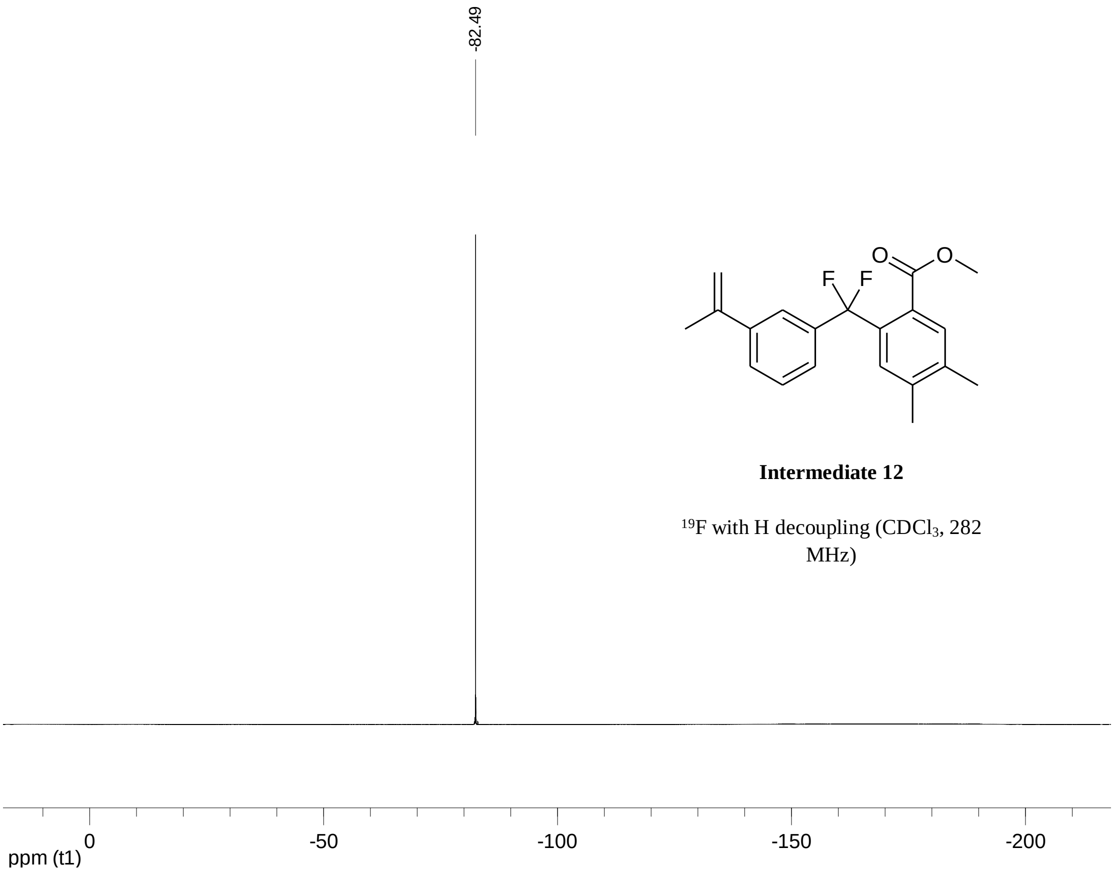

Intermediate 12

^{19}F with H decoupling ($CDCl_3$, 282 MHz)

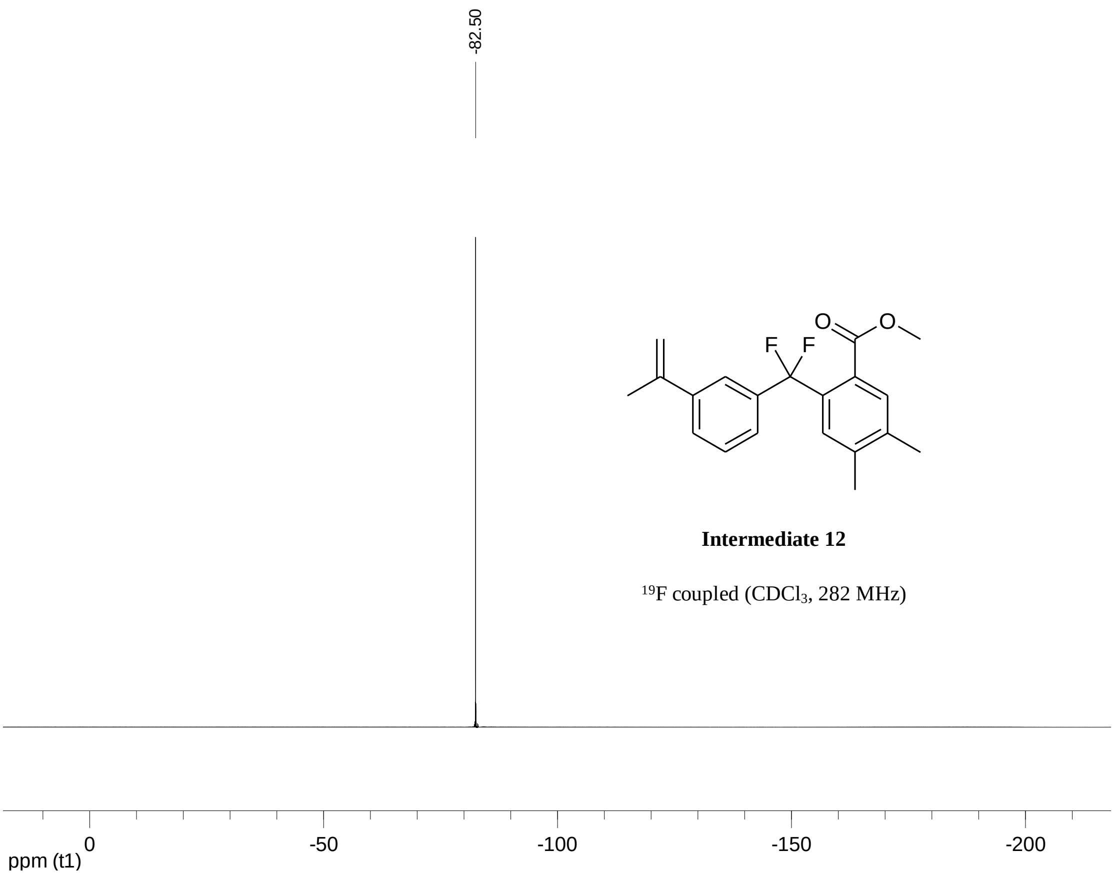

Intermediate 12

^{19}F coupled ($CDCl_3$, 282 MHz)

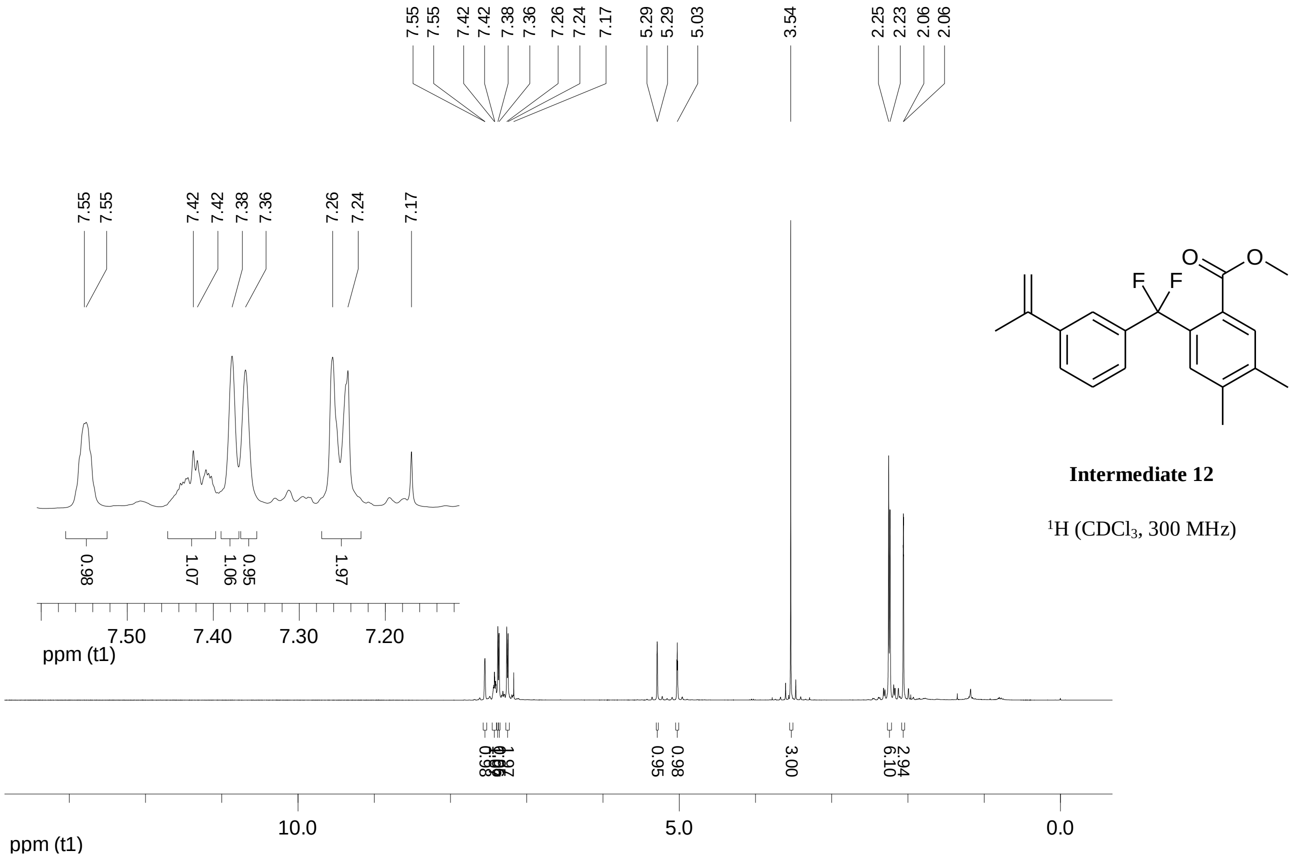

Intermediate 12

^{1}H ($CDCl_3$, 300 MHz)

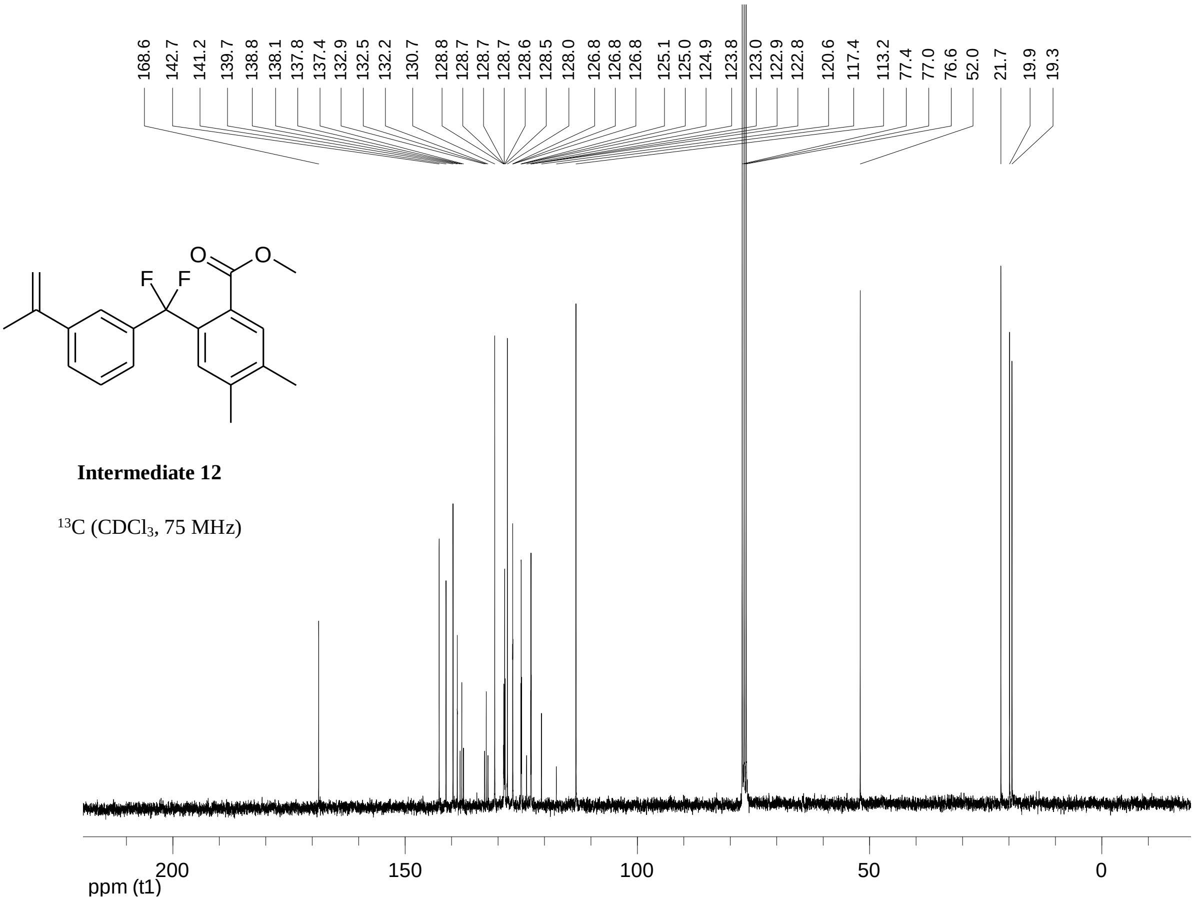

Intermediate 12

^{13}C ($CDCl_3$, 75 MHz)

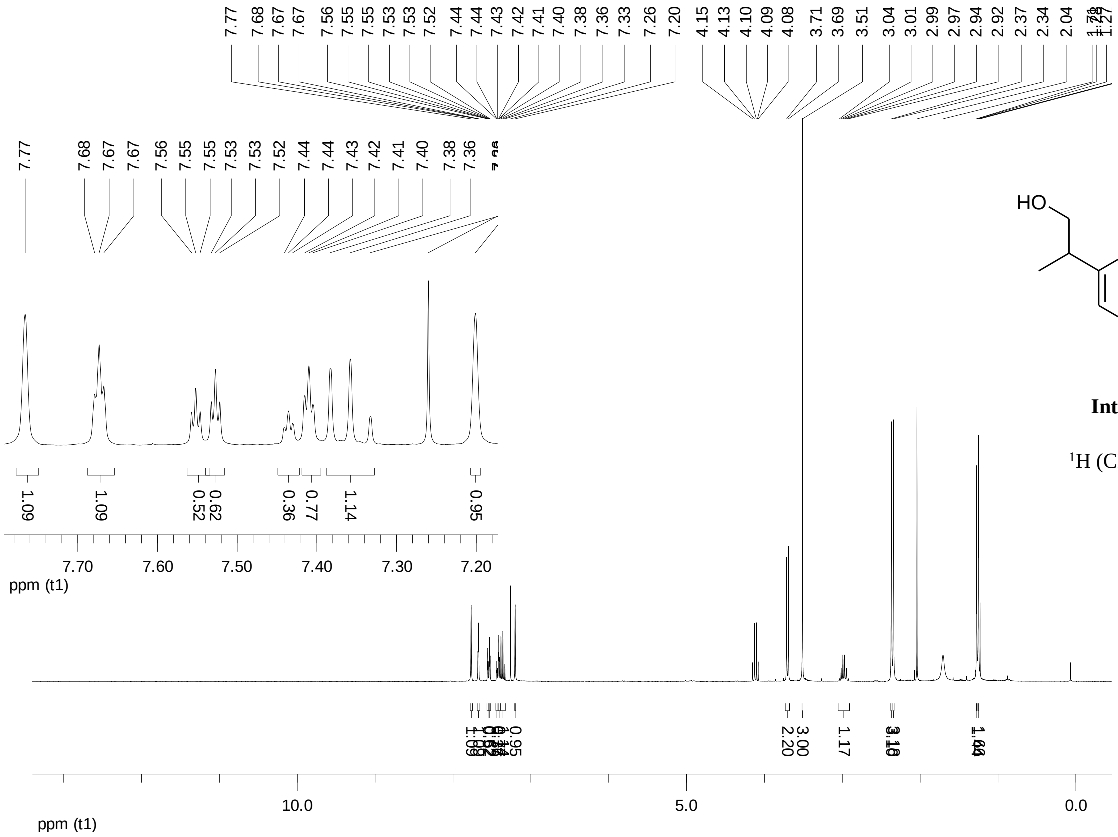

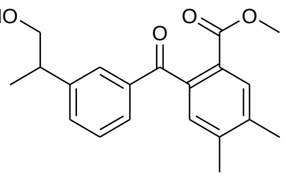

Intermediate 13

^{1}H ($CDCl_3$, 300 MHz)

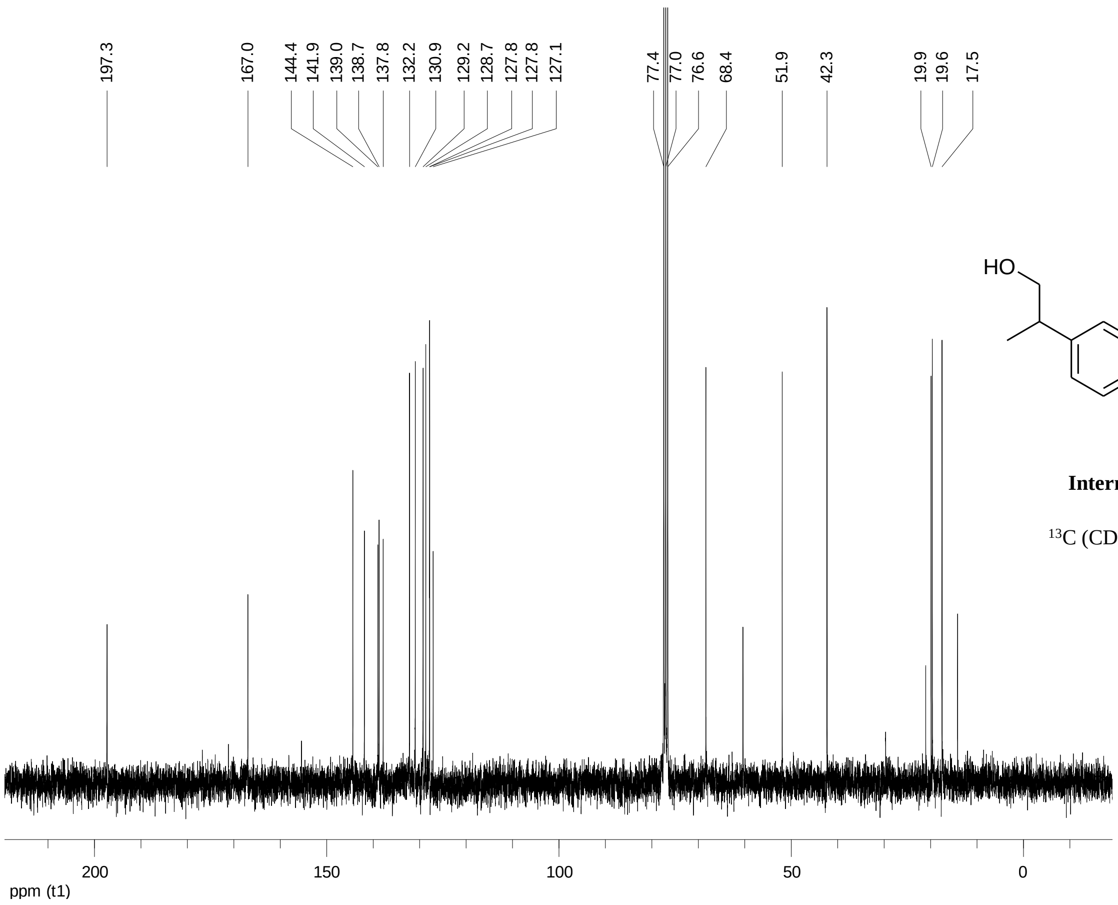

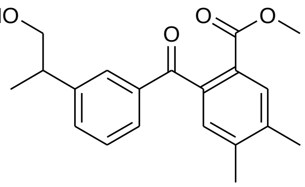

Intermediate 13

^{13}C ($CDCl_3$, 75 MHz)

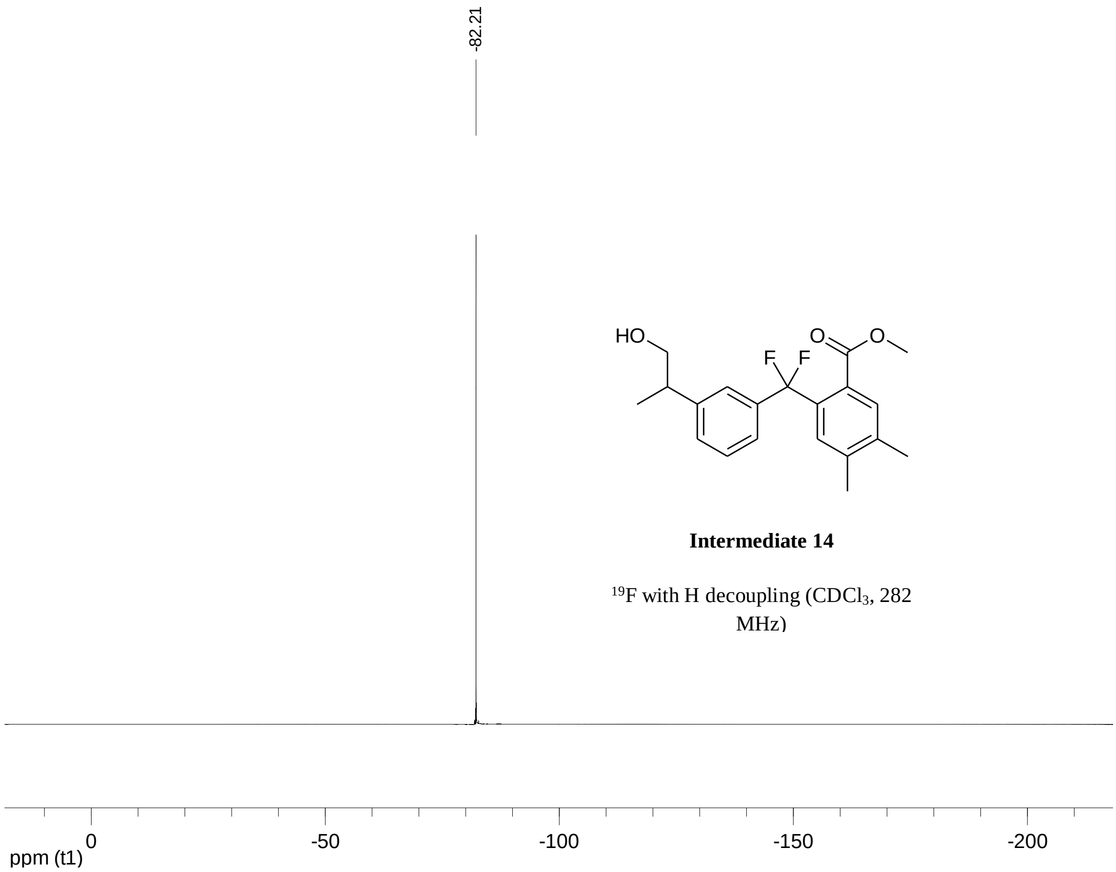

Intermediate 14

^{19}F with H decoupling ($CDCl_3$, 282 MHz)

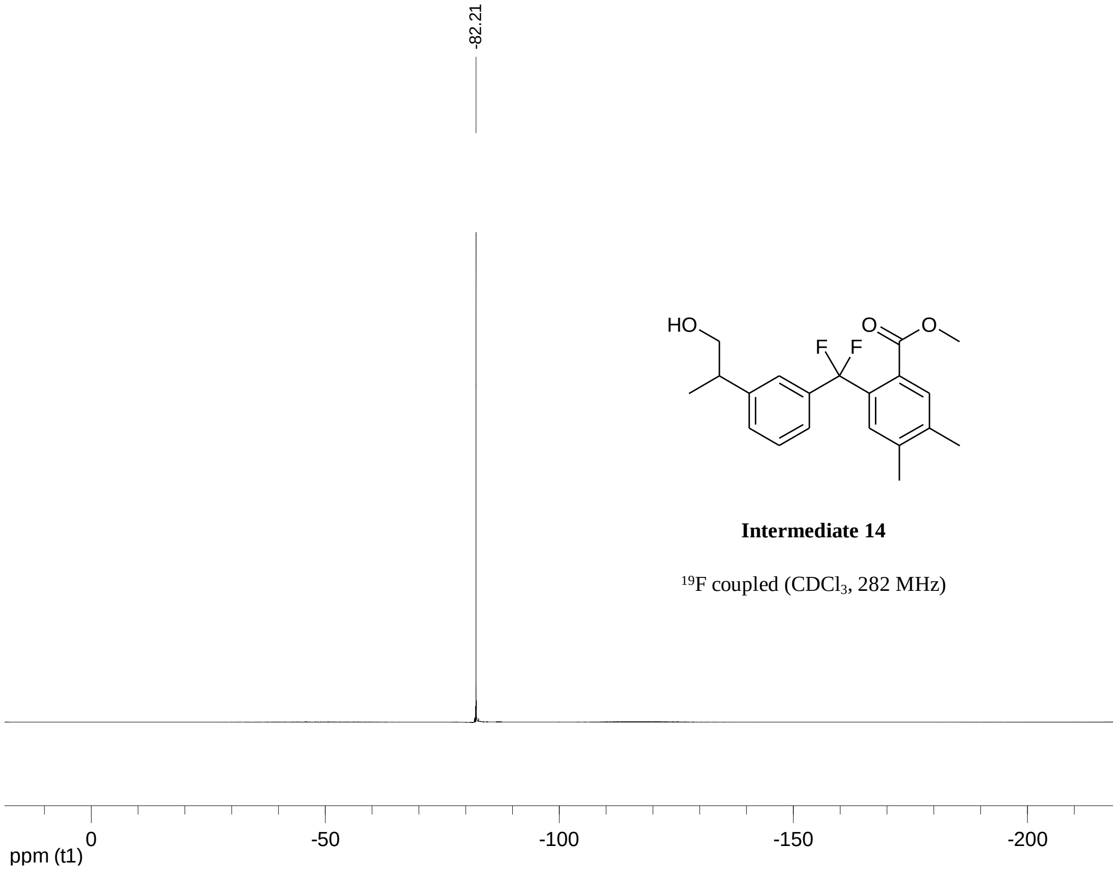

Intermediate 14

^{19}F coupled ($CDCl_3$, 282 MHz)

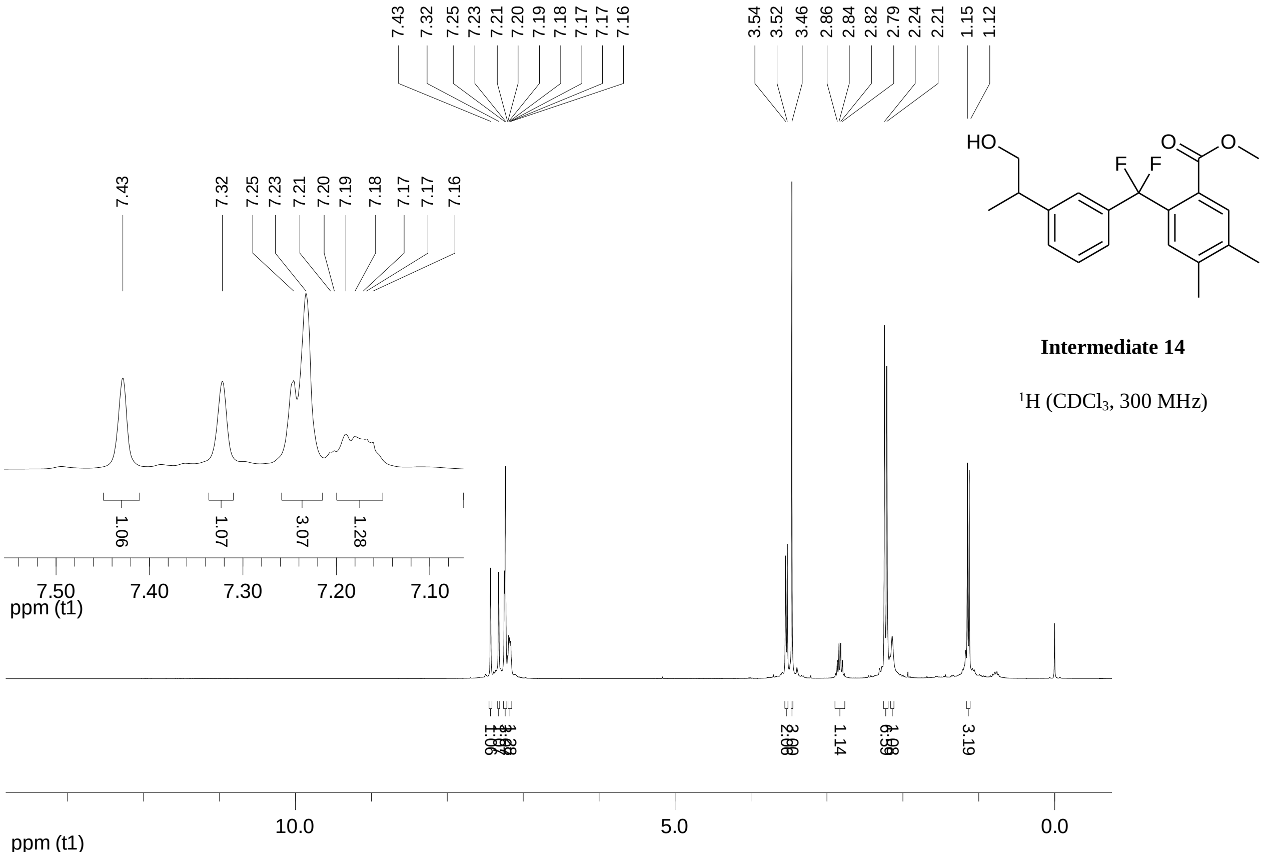

Intermediate 14

^{1}H ($CDCl_3$, 300 MHz)

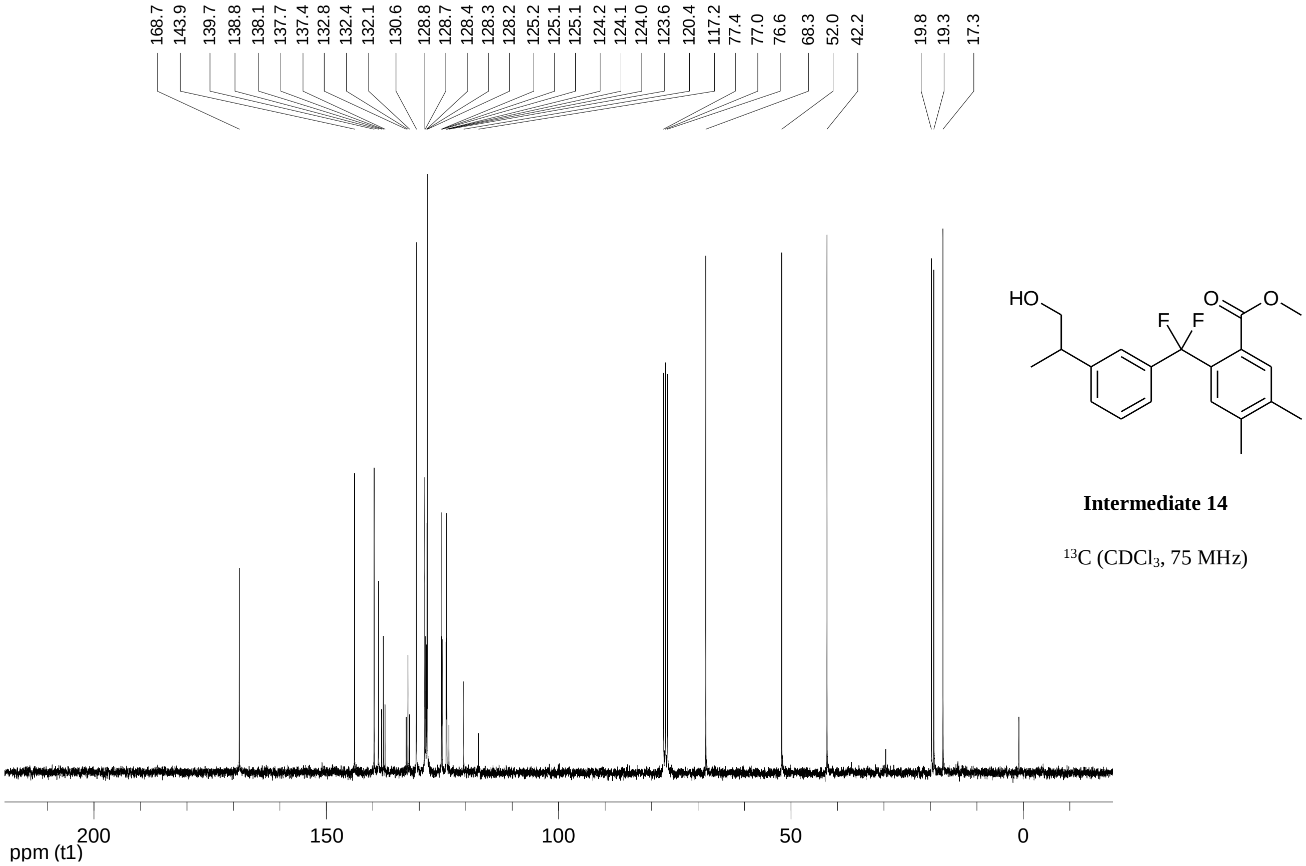

Intermediate 14

^{13}C ($CDCl_3$, 75 MHz)

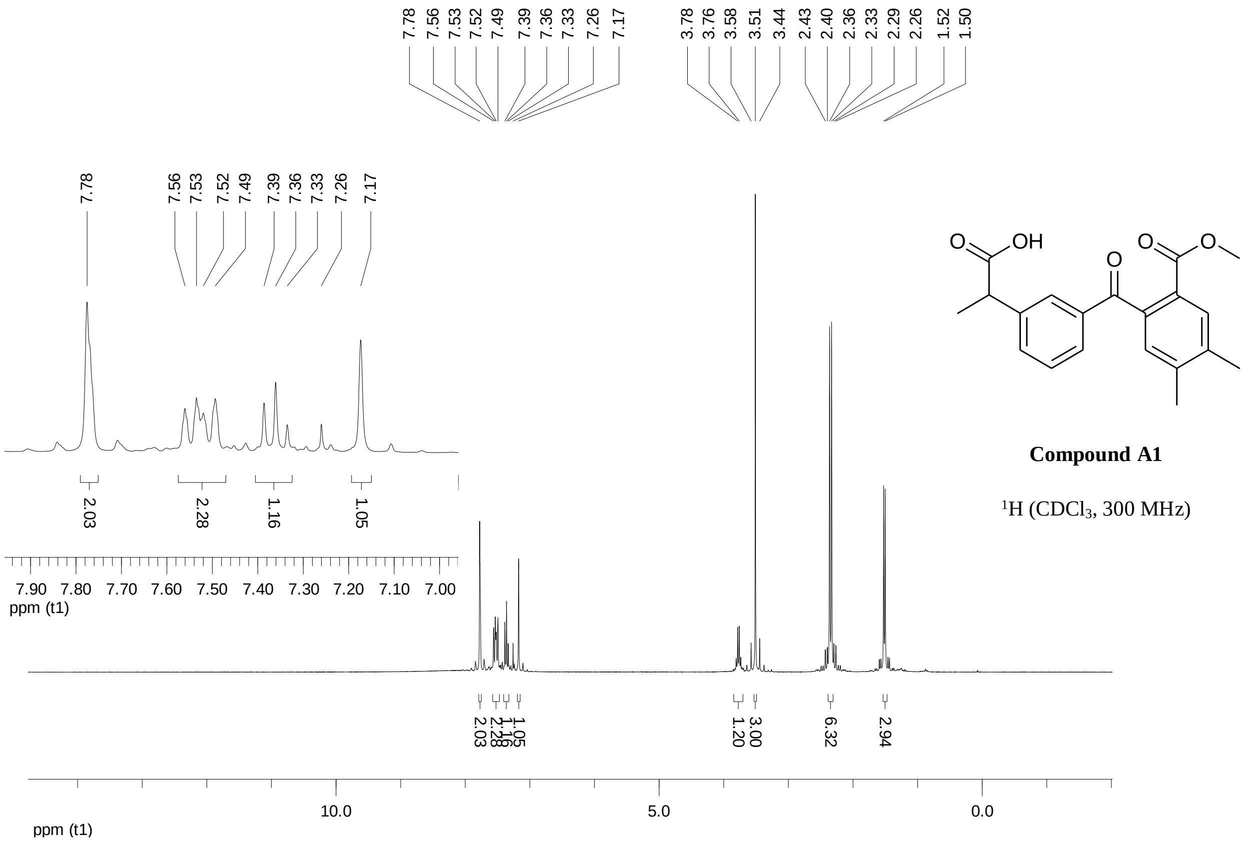
7.78 7.56 7.53 7.52 7.49 7.39 7.36 7.33 7.26 7.17
3.78 3.76 3.58 3.51 3.44 2.43 2.40 2.36 2.33 2.29 2.26 1.52 1.50
2.03 2.28 1.16 1.05
7.90 7.80 7.70 7.60 7.50 7.40 7.30 7.20 7.10 7.00
ppm (t1)
2.03 2.28 1.16 1.05 1.20 3.00 6.32 2.94
10.0 5.0 0.0
ppm (t1)
O OH O O O
Compound A1
^{1}H (CDCl$_3$, 300 MHz)

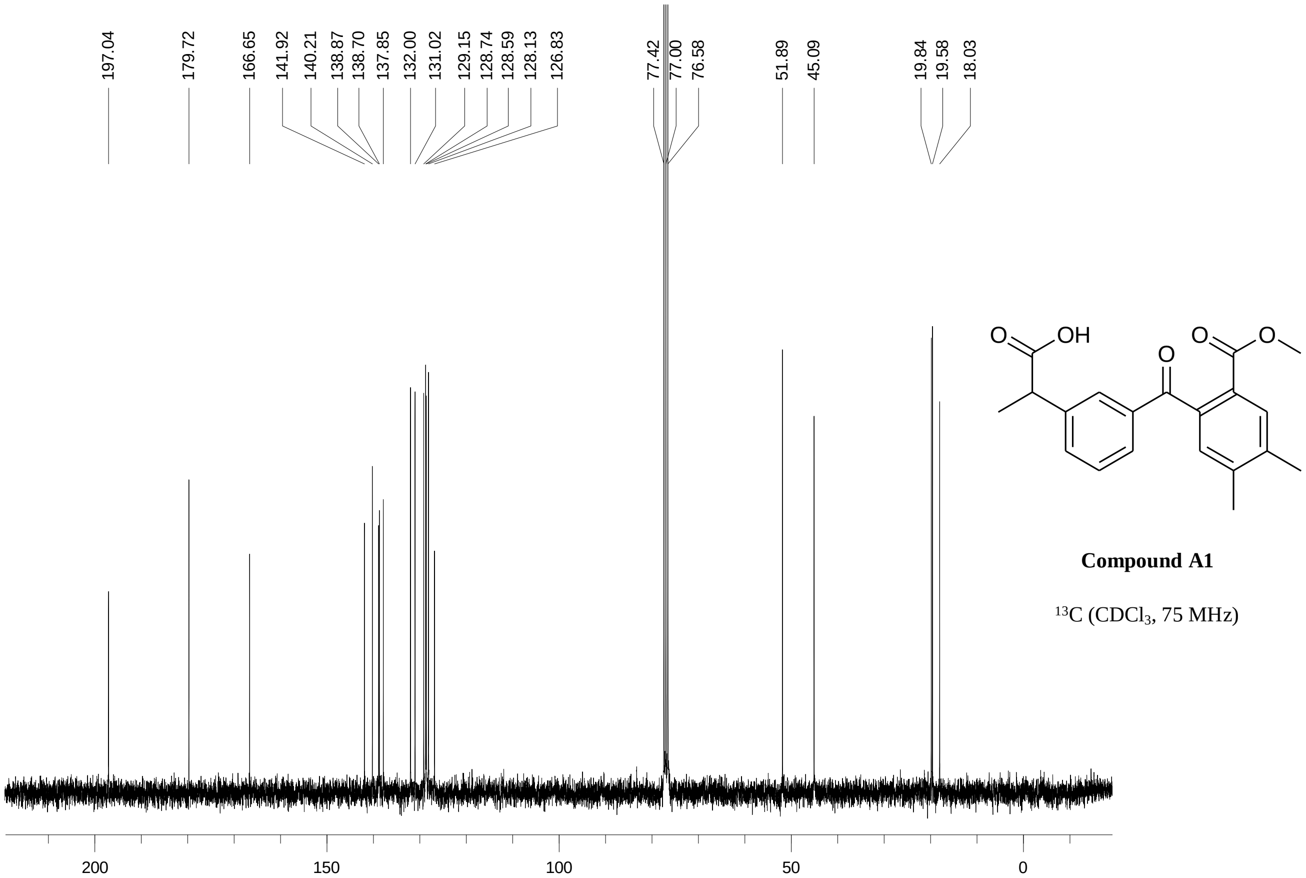
197.04 179.72 166.65 141.92 140.21 138.87 138.70 137.85 132.00 131.02 129.15 128.74 128.59 128.13 126.83
77.42 77.00 76.58 51.89 45.09 19.84 19.58 18.03
O OH O O O
Compound A1
^{13}C (CDCl$_3$, 75 MHz)
200 150 100 50 0
ppm (t1)

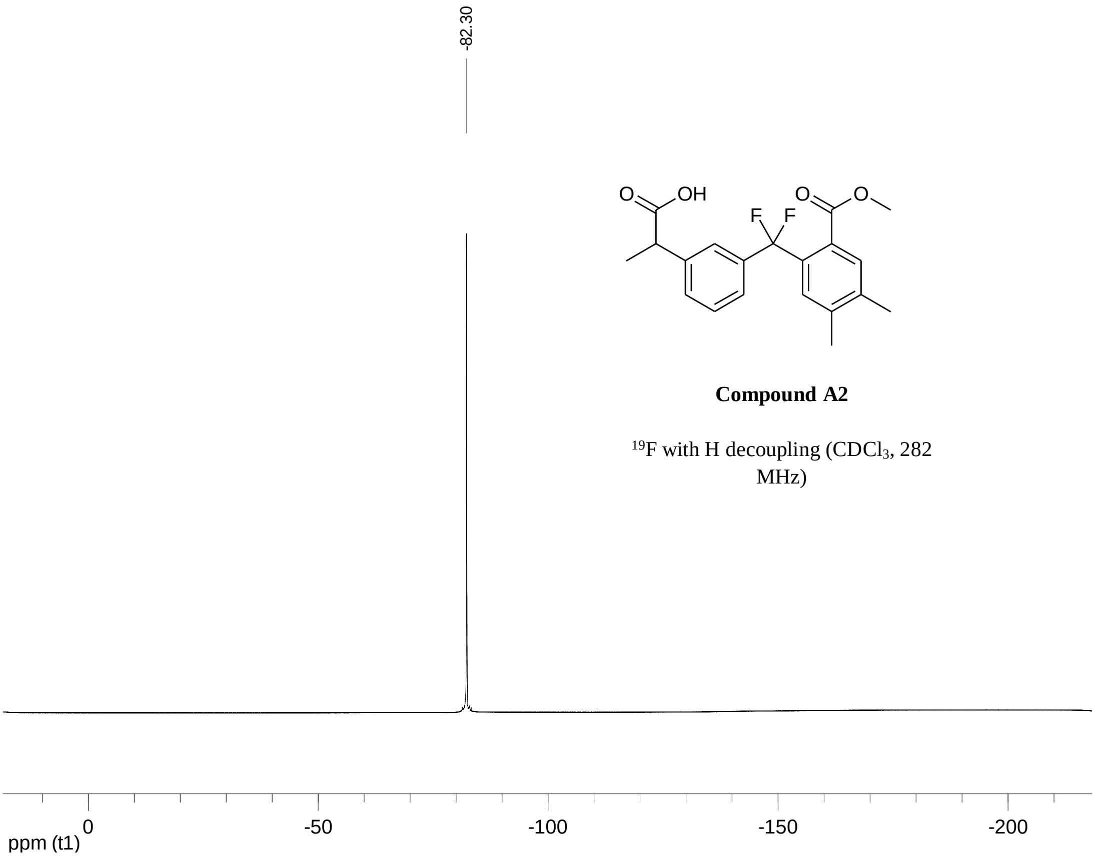

Compound A2

^{19}F with H decoupling ($CDCl_3$, 282 MHz)

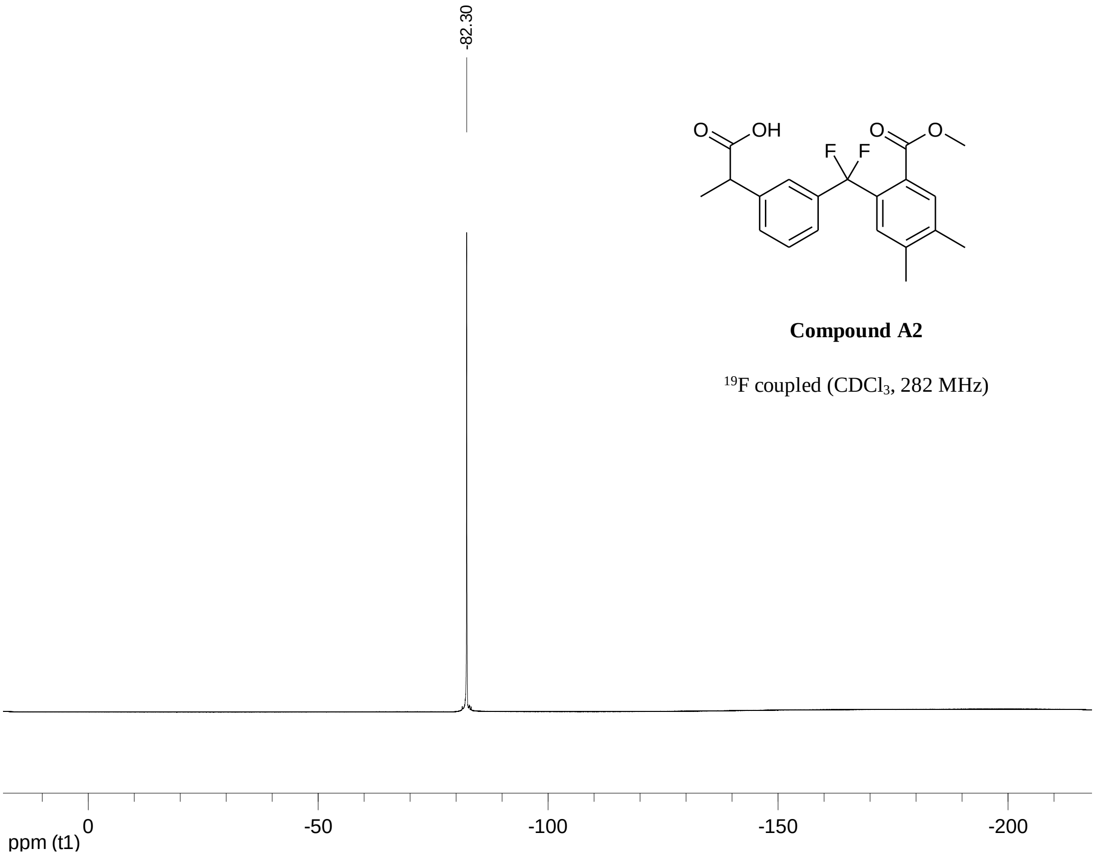

Compound A2

^{19}F coupled ($CDCl_3$, 282 MHz)

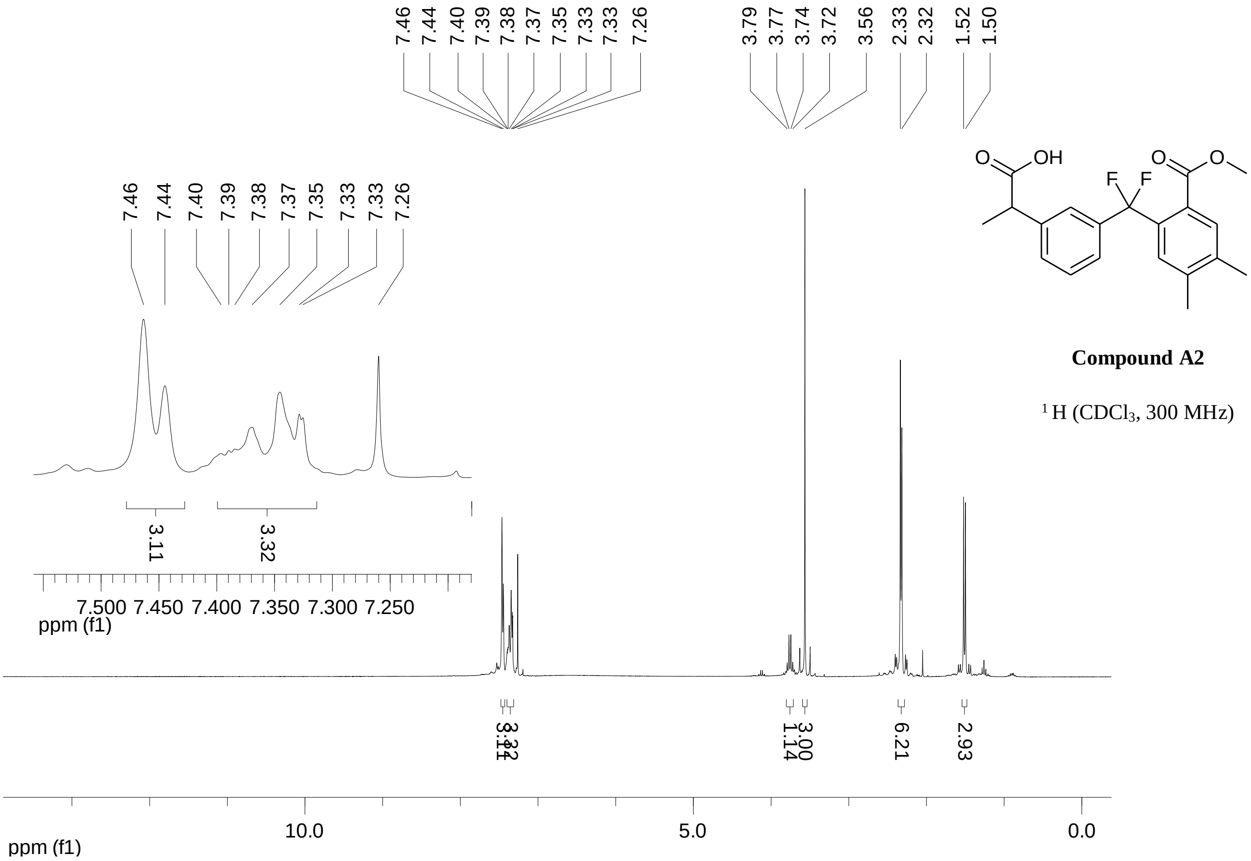

Compound A2

^{1}H ($CDCl_3$, 300 MHz)

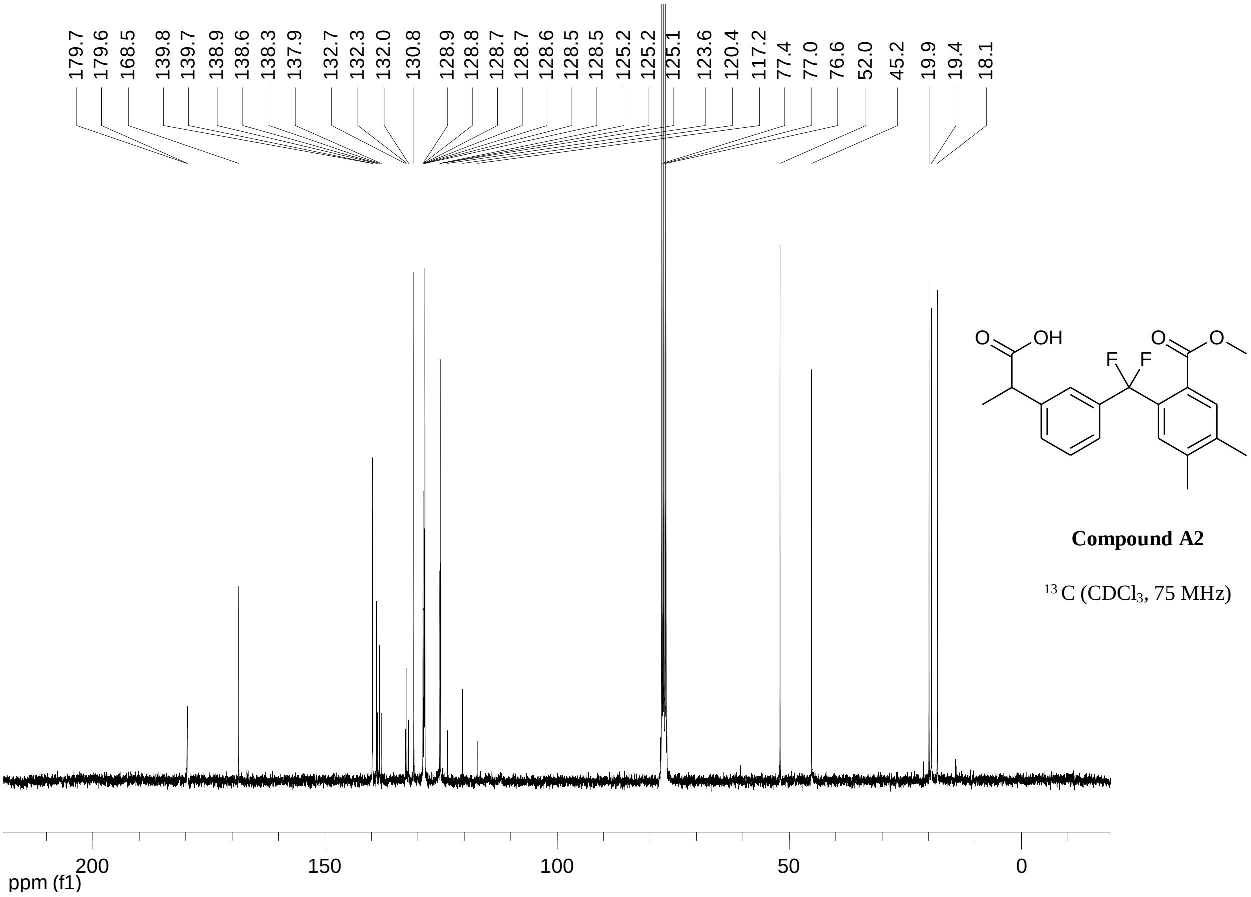

Compound A2

^{13}C ($CDCl_3$, 75 MHz)

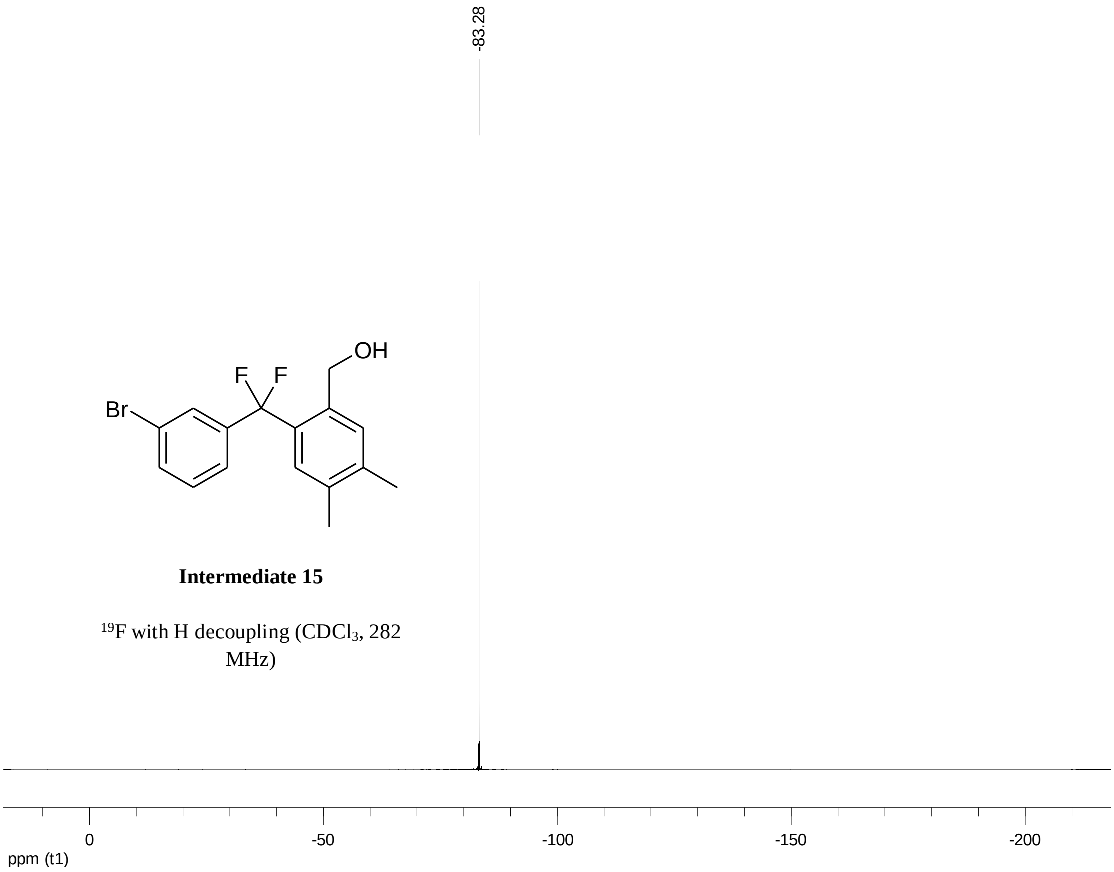

Intermediate 15

^{19}F with H decoupling ($CDCl_3$, 282 MHz)

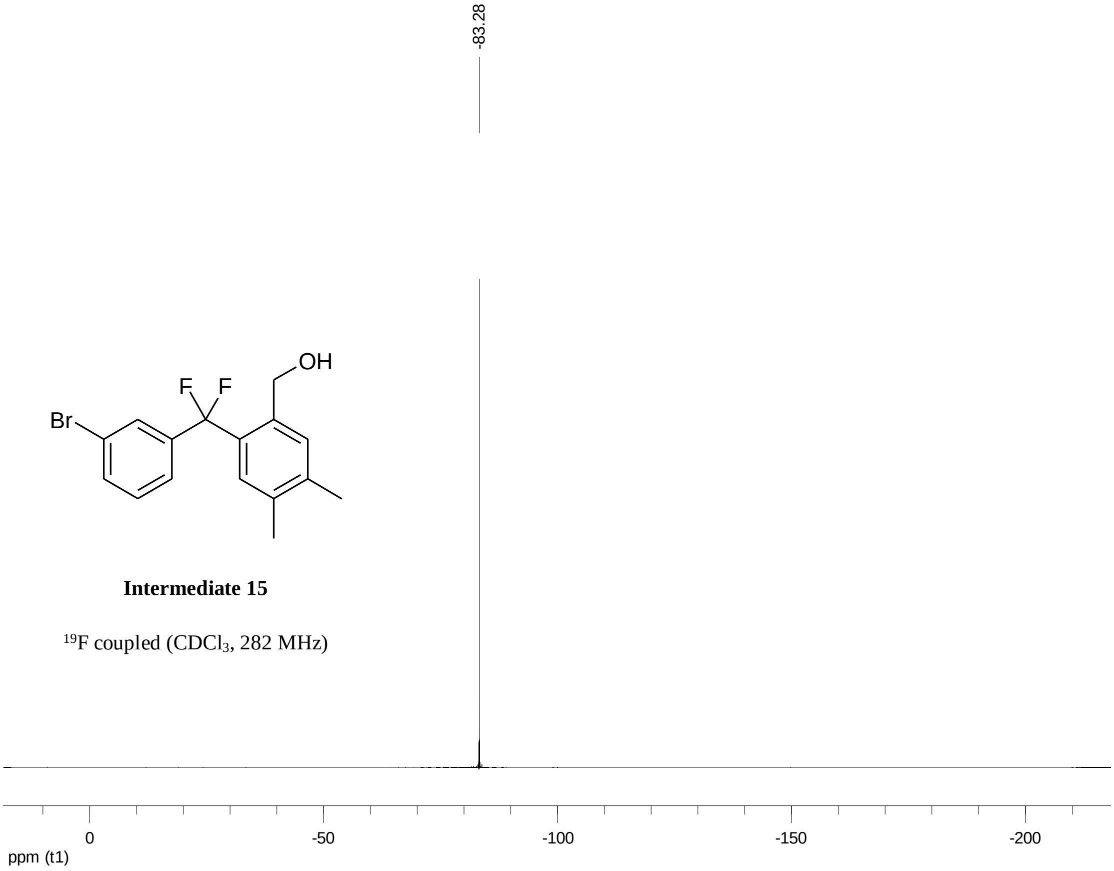

Intermediate 15

^{19}F coupled ($CDCl_3$, 282 MHz)

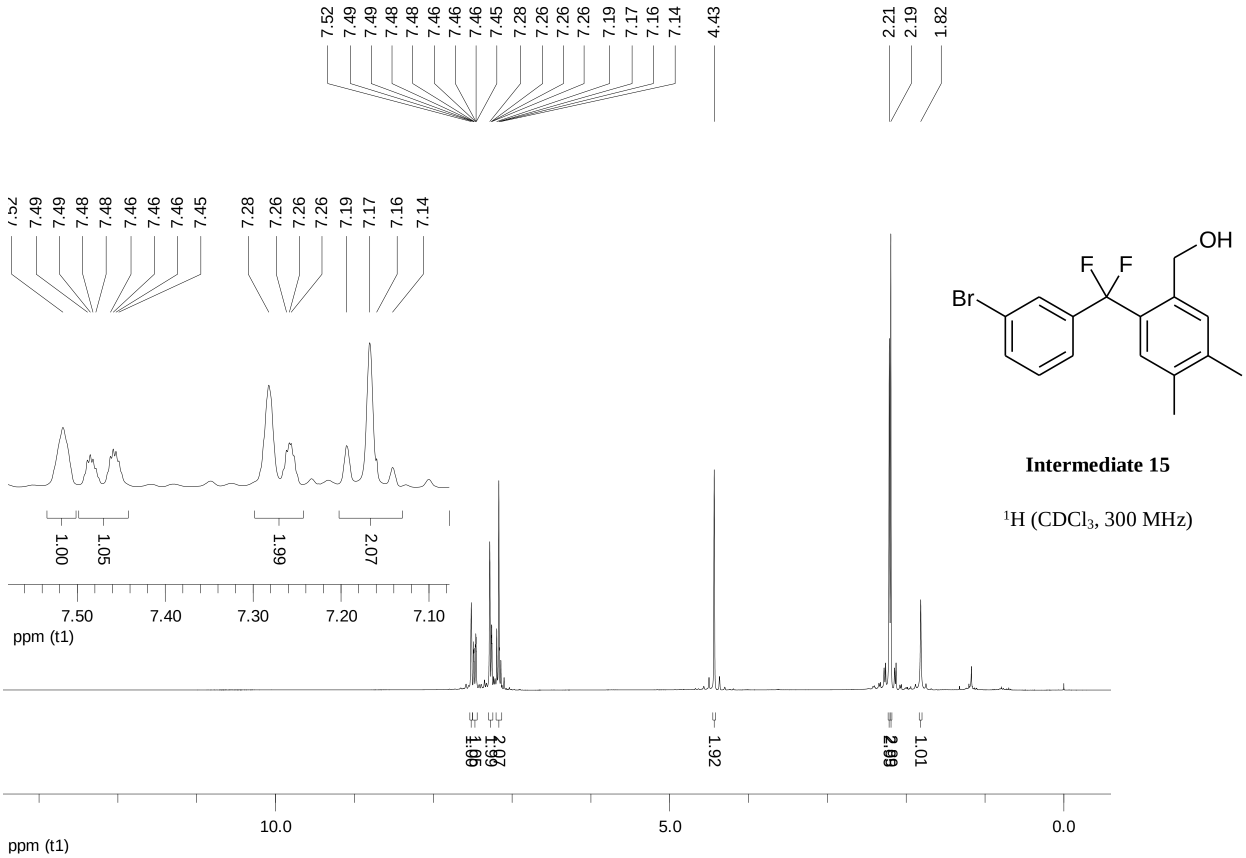

Intermediate 15

^{1}H ($CDCl_3$, 300 MHz)

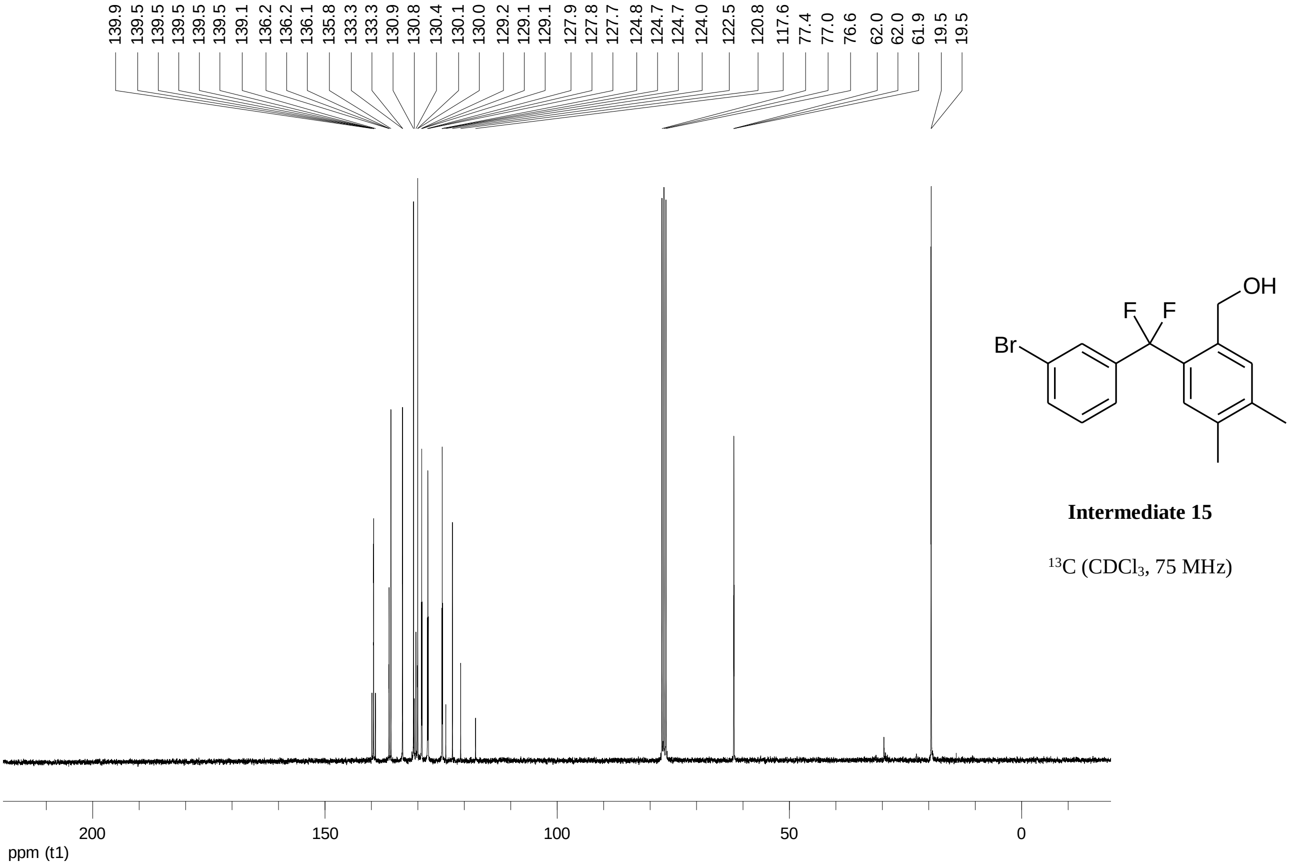

Intermediate 15

^{13}C ($CDCl_3$, 75 MHz)

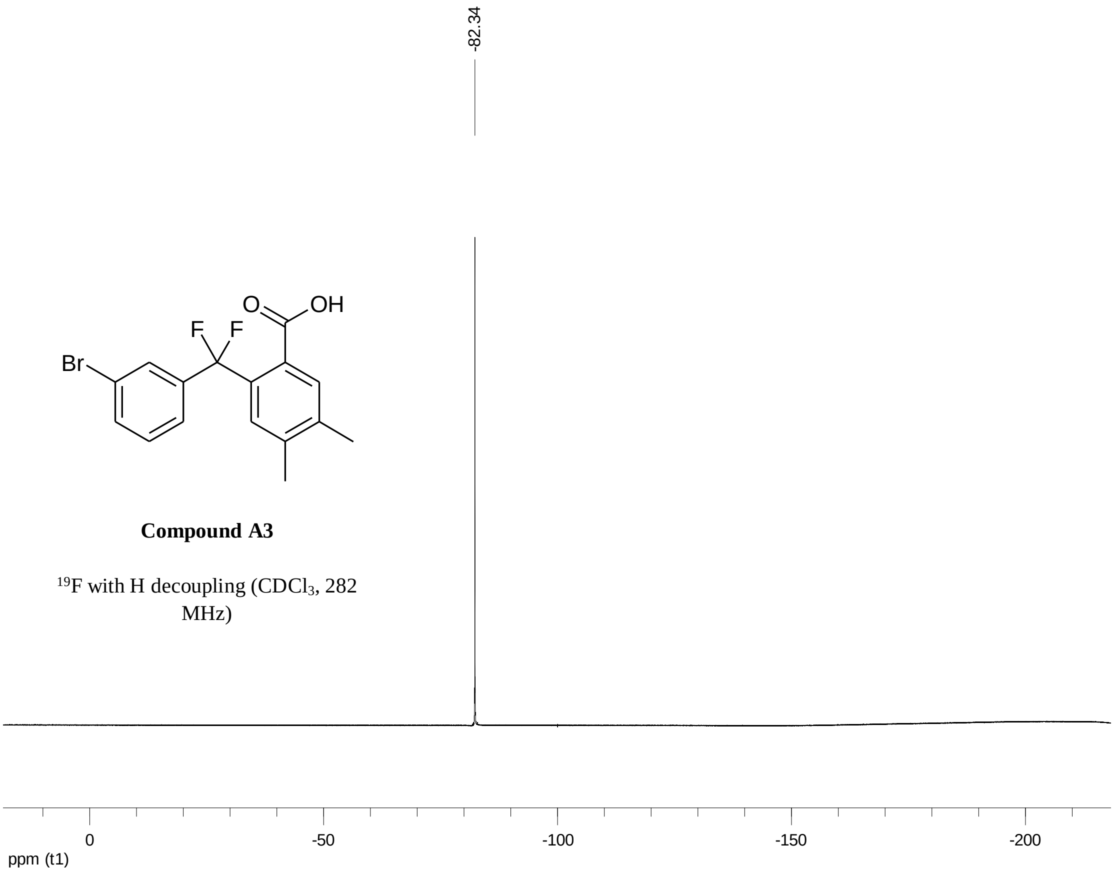
-82.34
Br
F F
O OH
Compound A3
^{19}F with H decoupling ($CDCl_3$, 282 MHz)
0
-50
-100
-150
-200
ppm (t1)

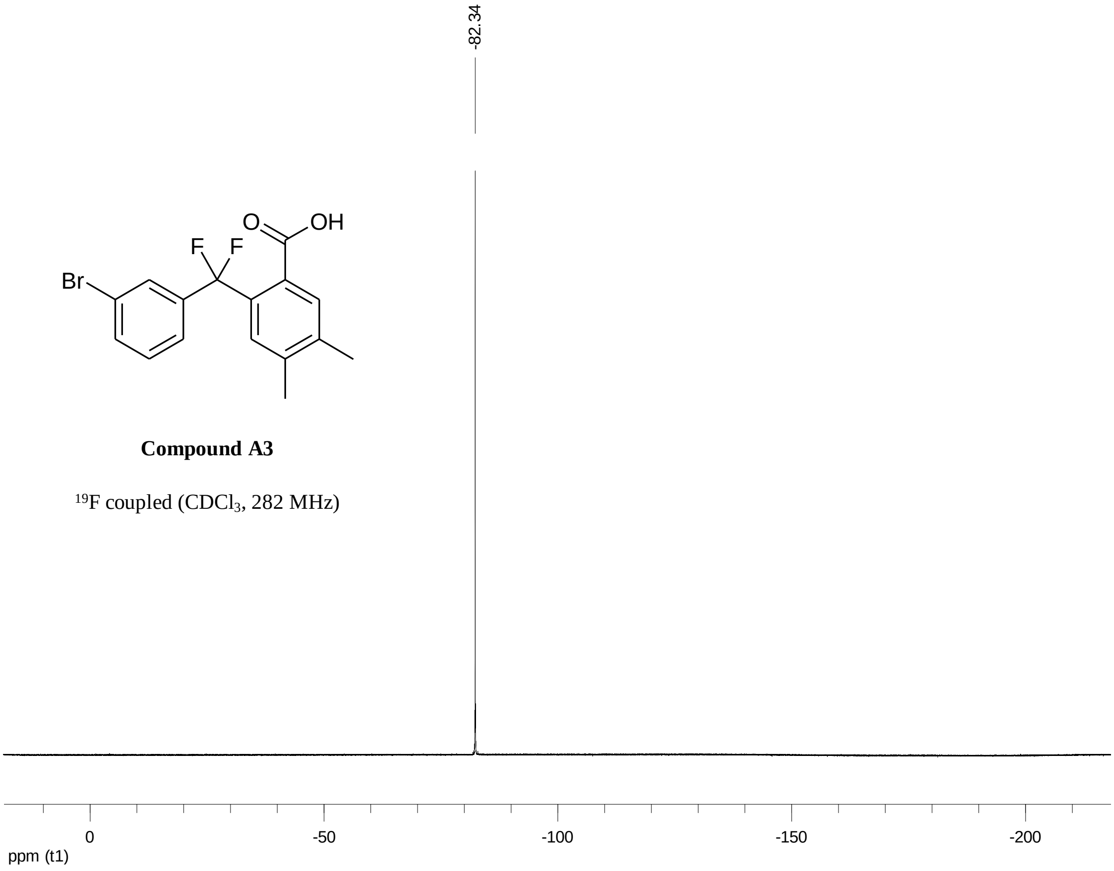
-82.34
Br
F F
O OH
Compound A3
^{19}F coupled ($CDCl_3$, 282 MHz)
0
-50
-100
-150
-200
ppm (t1)

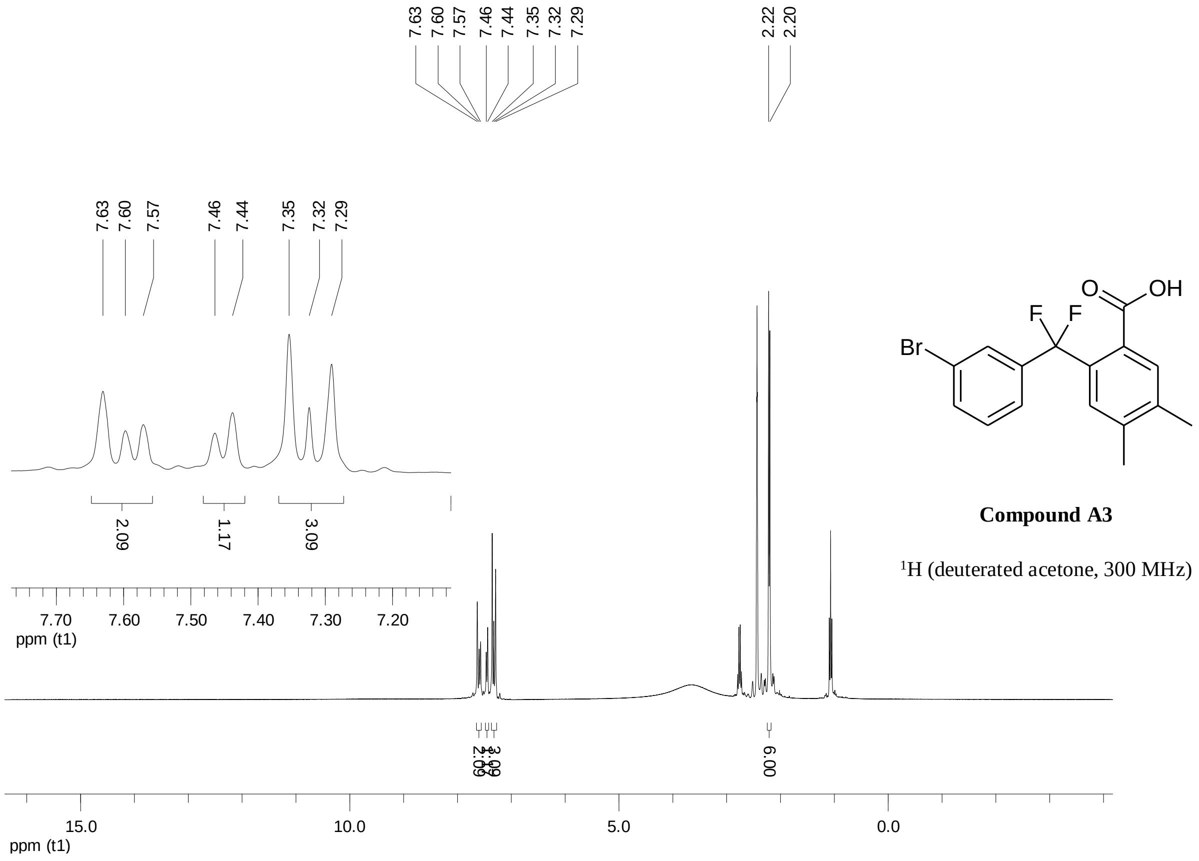

Compound A3

^{1}H (deuterated acetone, 300 MHz)

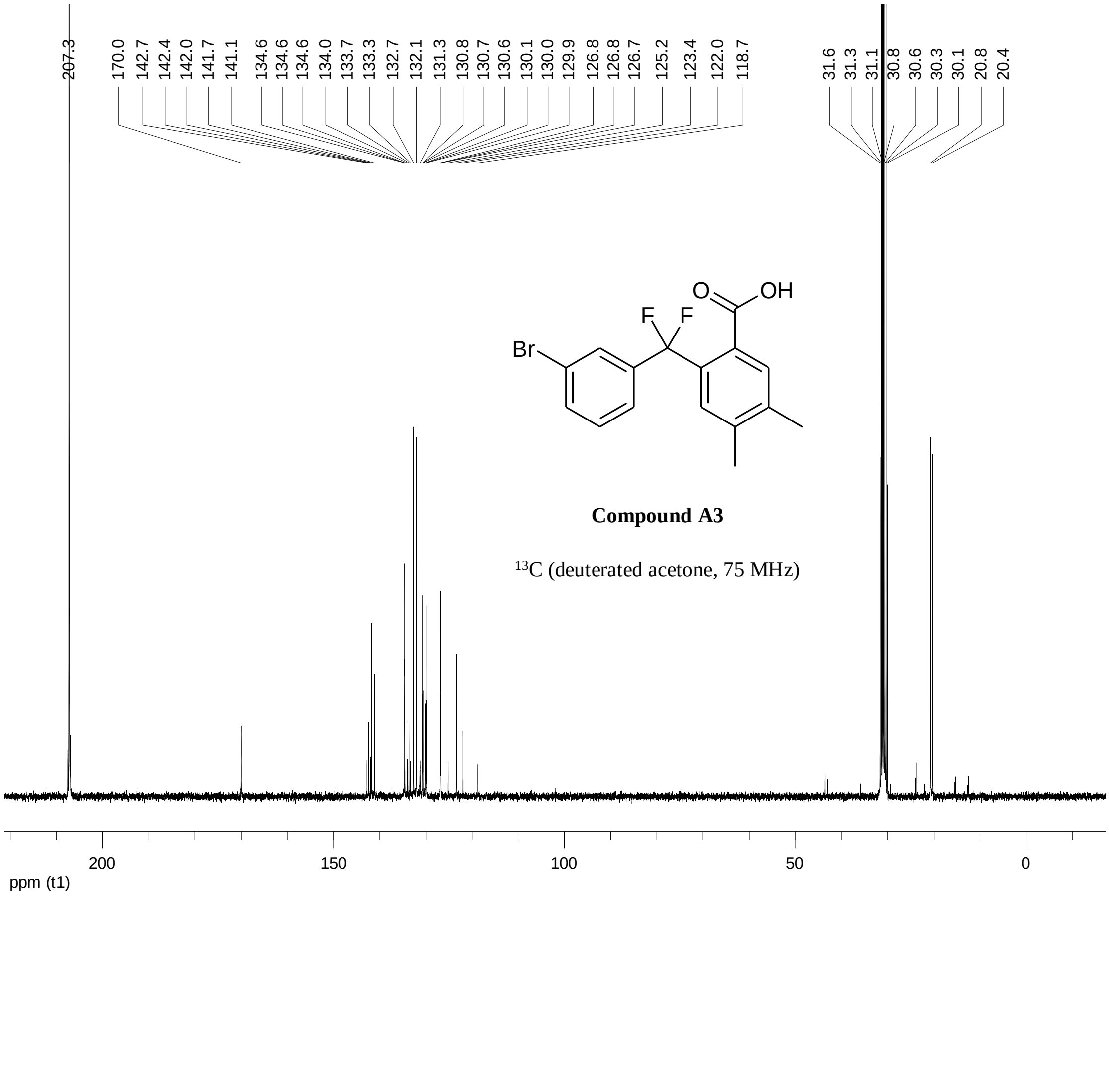

Compound A3

^{13}C (deuterated acetone, 75 MHz)

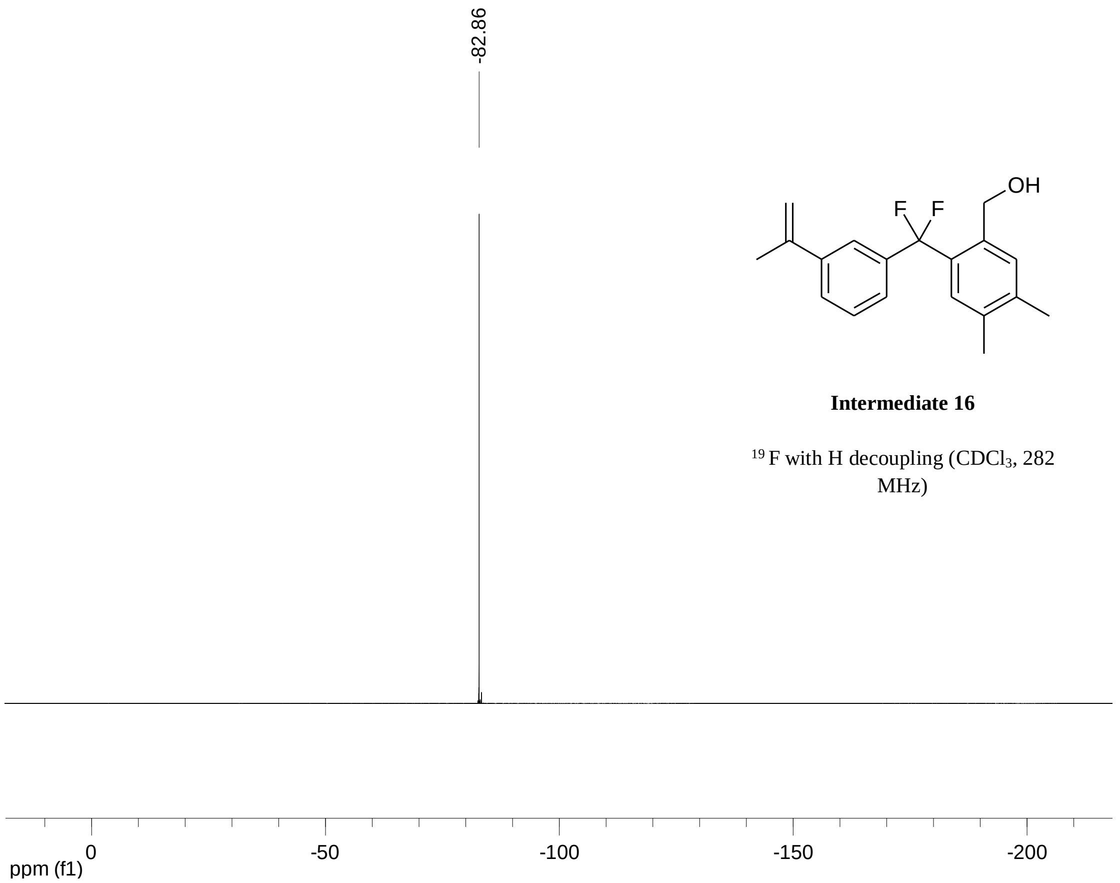
-82.86
OH
F F
Intermediate 16
^{19}F with H decoupling ($CDCl_3$, 282 MHz)
0
-50
-100
-150
-200
ppm (f1)

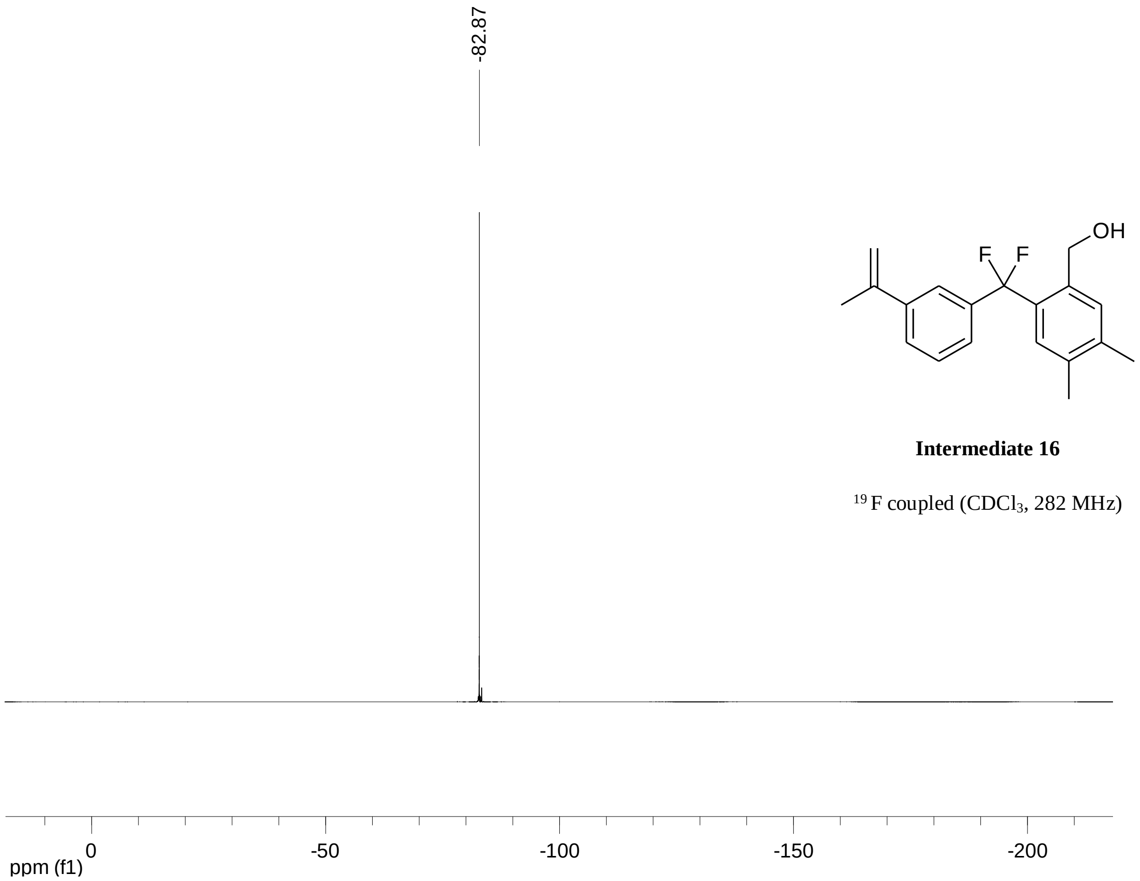
-82.87
OH
F F
Intermediate 16
^{19}F coupled ($CDCl_3$, 282 MHz)
0
-50
-100
-150
-200
ppm (f1)

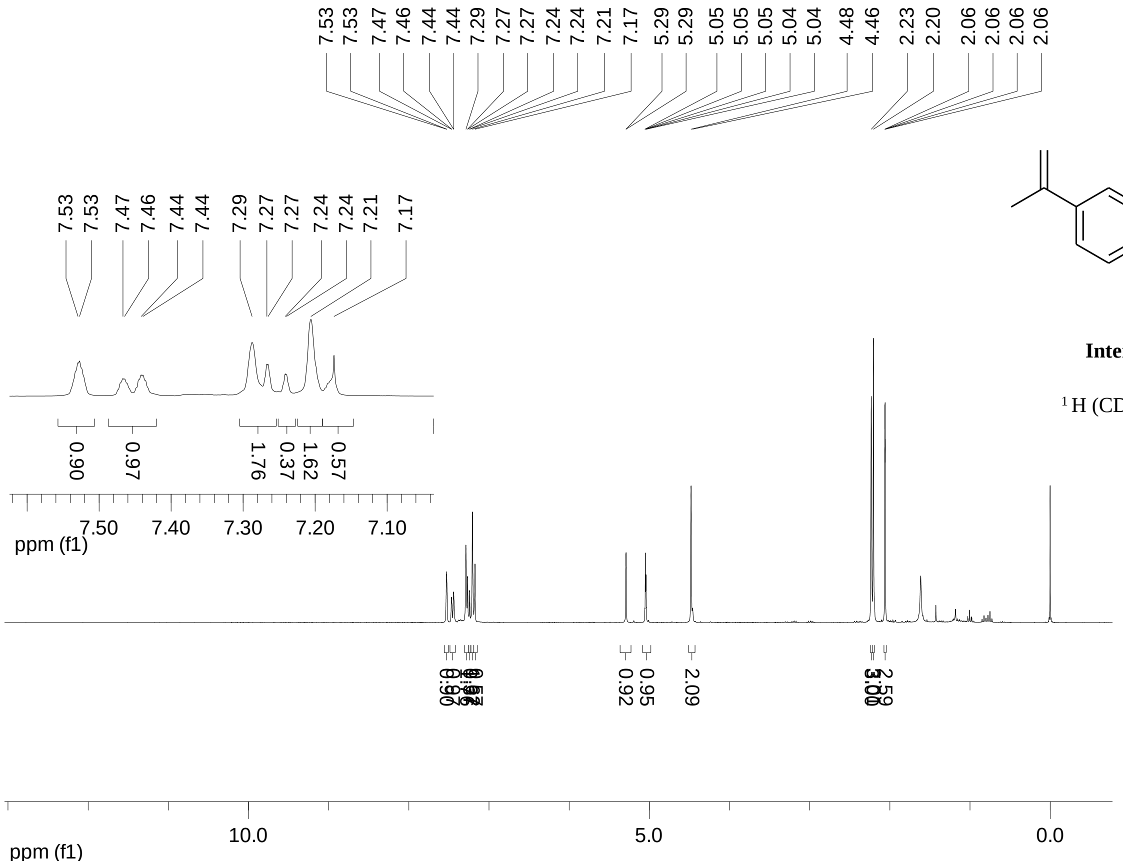

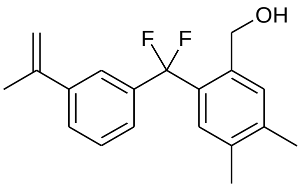

Intermediate 16

^{1}H ($CDCl_3$, 300 MHz)

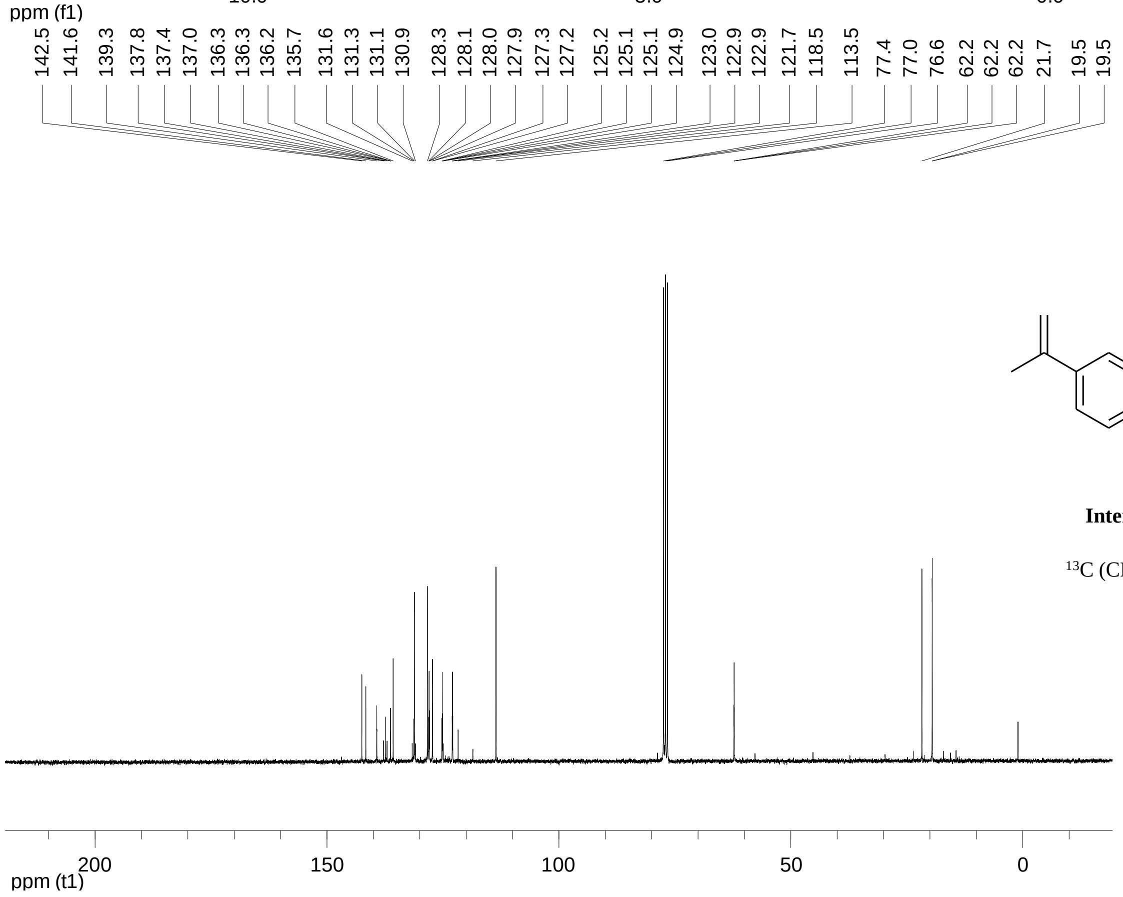

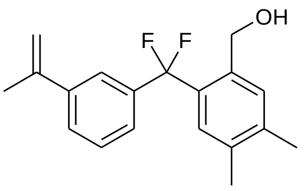

Intermediate 16

^{13}C ($CDCl_3$, 75 MHz)

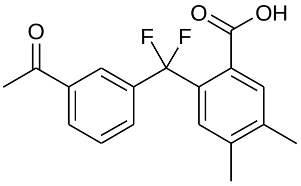

Compound A4

^{19}F with H decoupling ($CDCl_3$, 282 MHz)

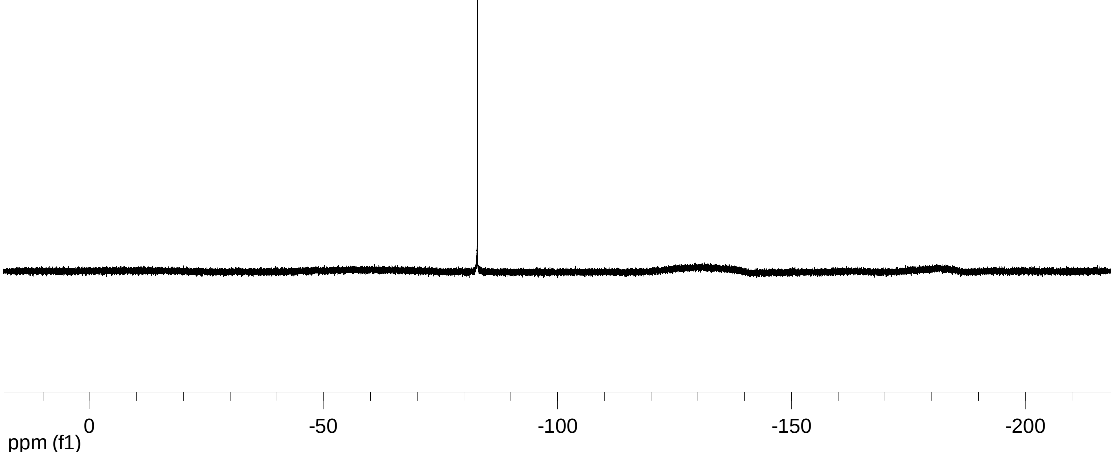

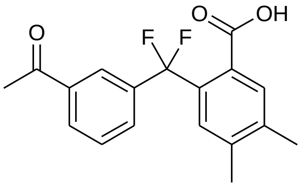

Compound A4

^{19}F coupled ($CDCl_3$, 282 MHz)

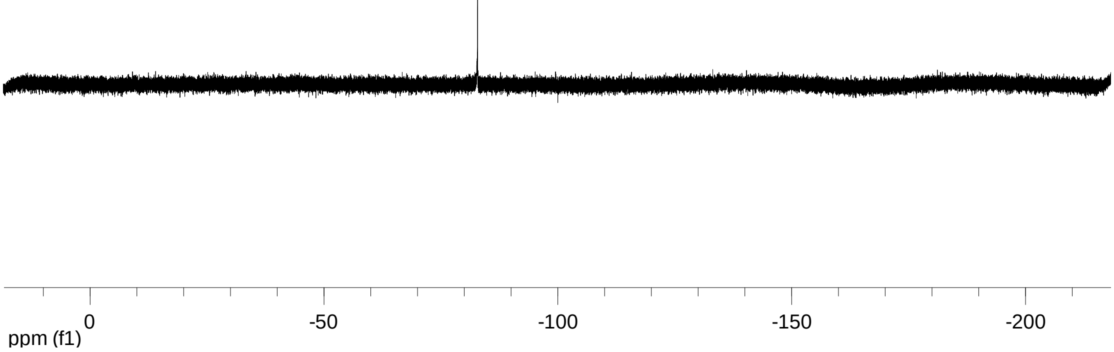

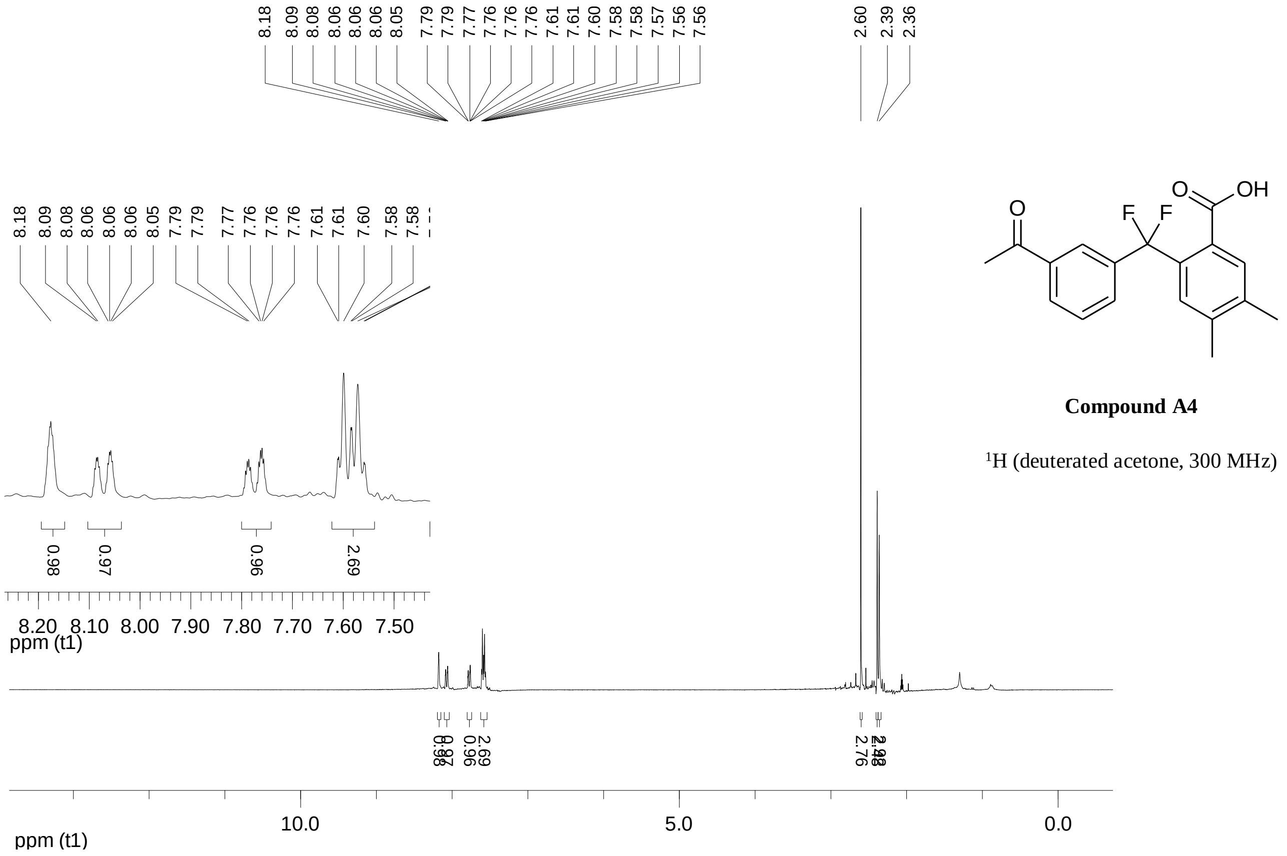

Compound A4

^{1}H (deuterated acetone, 300 MHz)

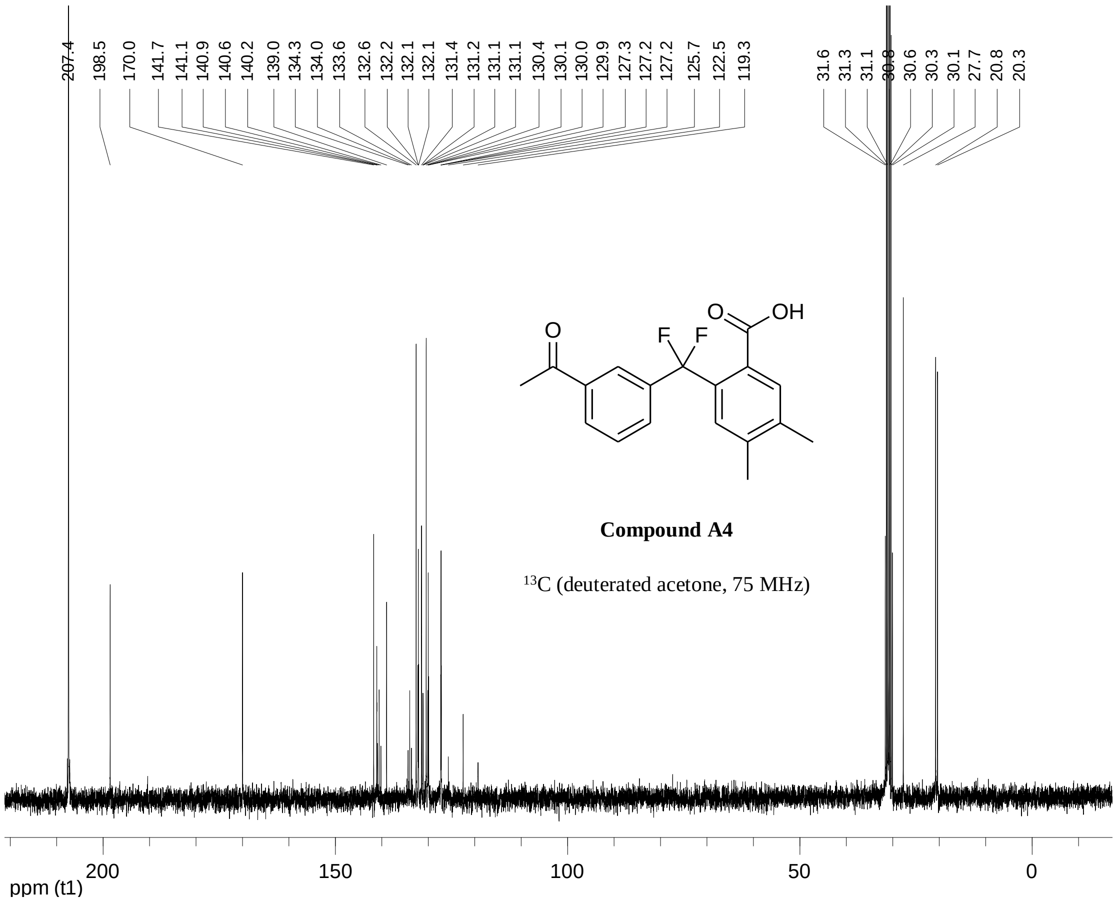

Compound A4

^{13}C (deuterated acetone, 75 MHz)

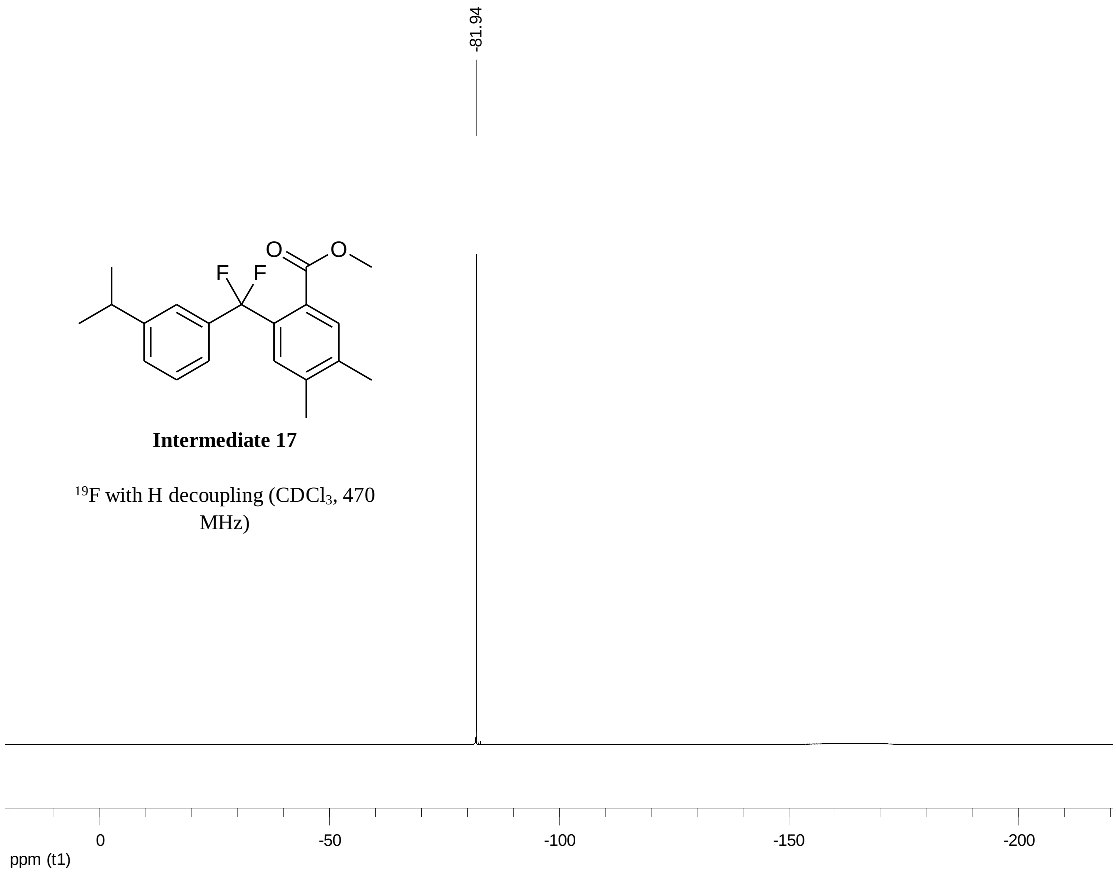

Intermediate 17

^{19}F with H decoupling ($CDCl_3$, 470 MHz)

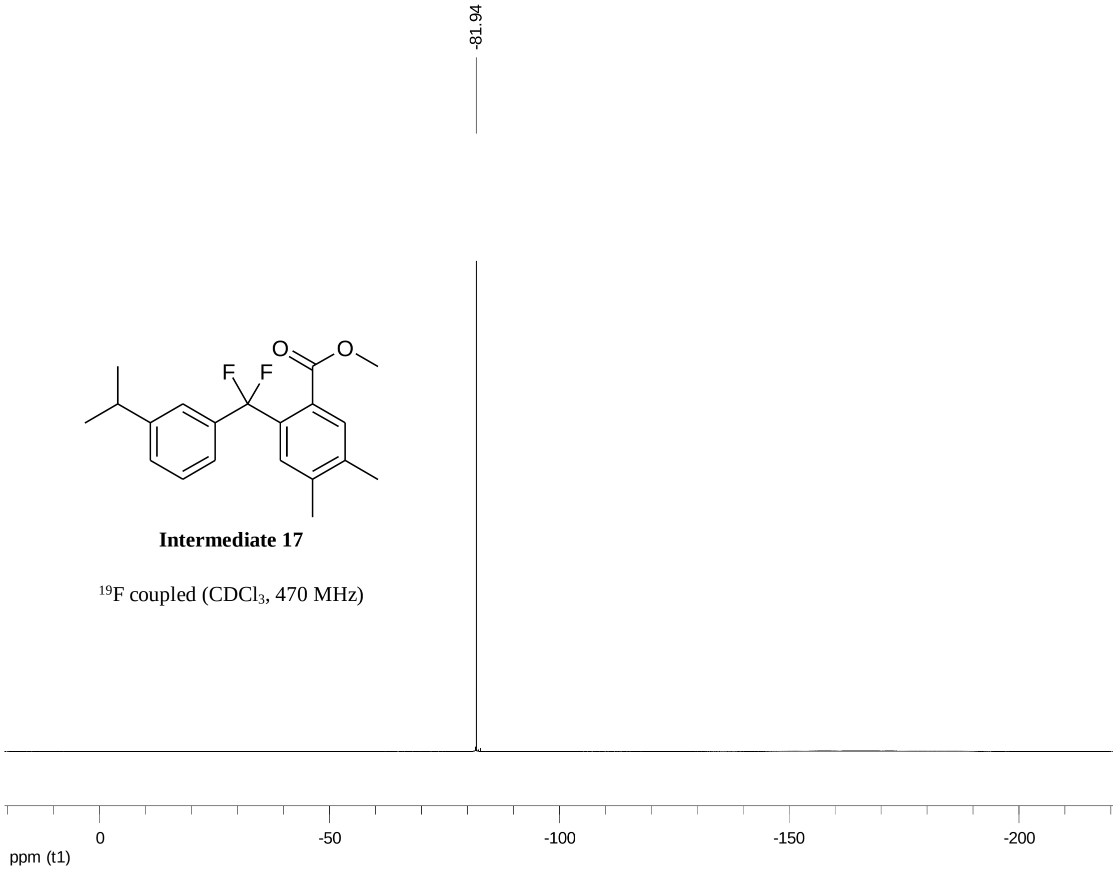

Intermediate 17

^{19}F coupled ($CDCl_3$, 470 MHz)

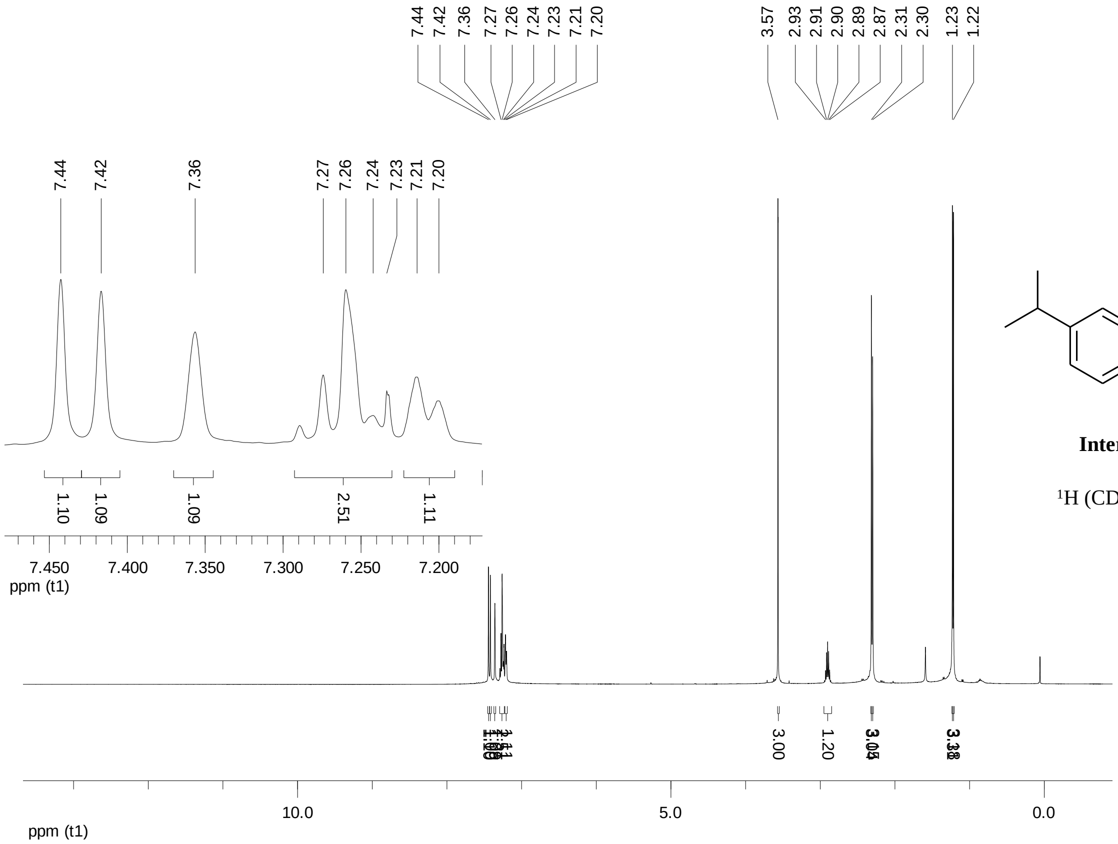

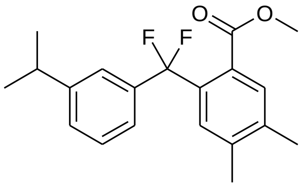

Intermediate 17

^{1}H ($CDCl_3$, 500 MHz)

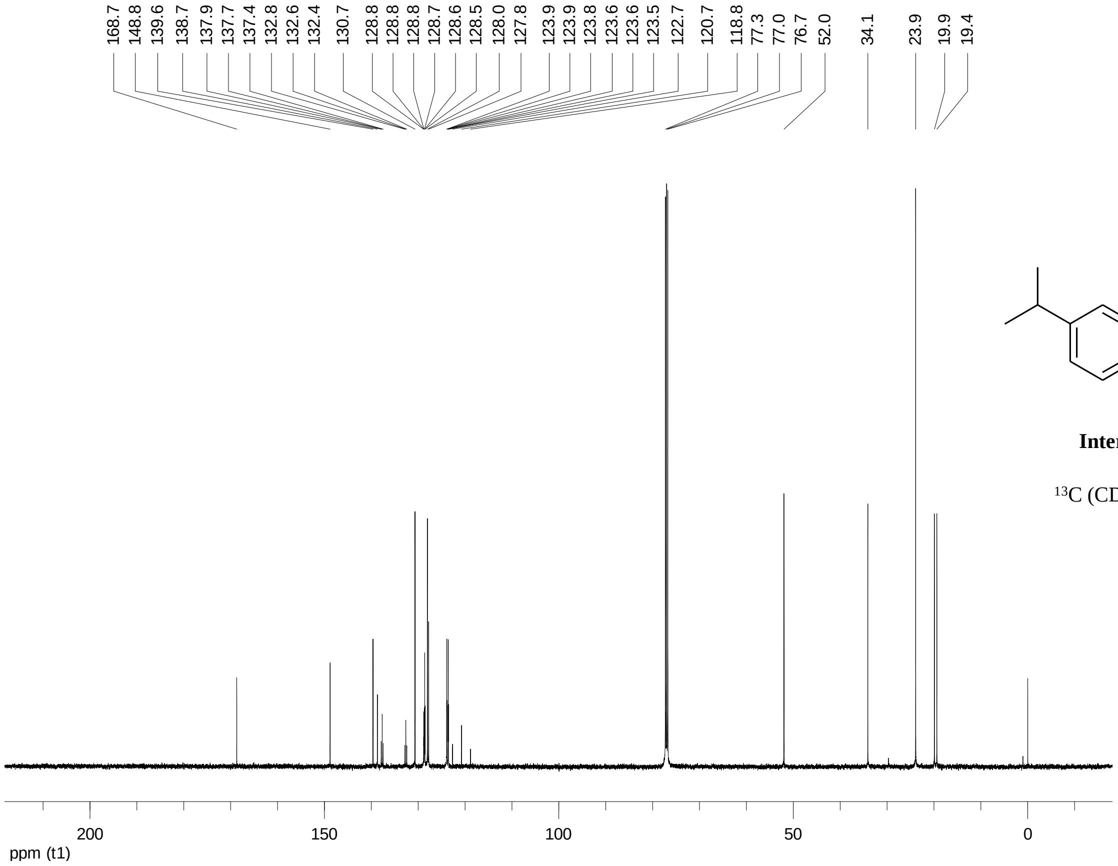

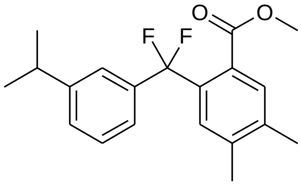

Intermediate 17

^{13}C ($CDCl_3$, 125 MHz)

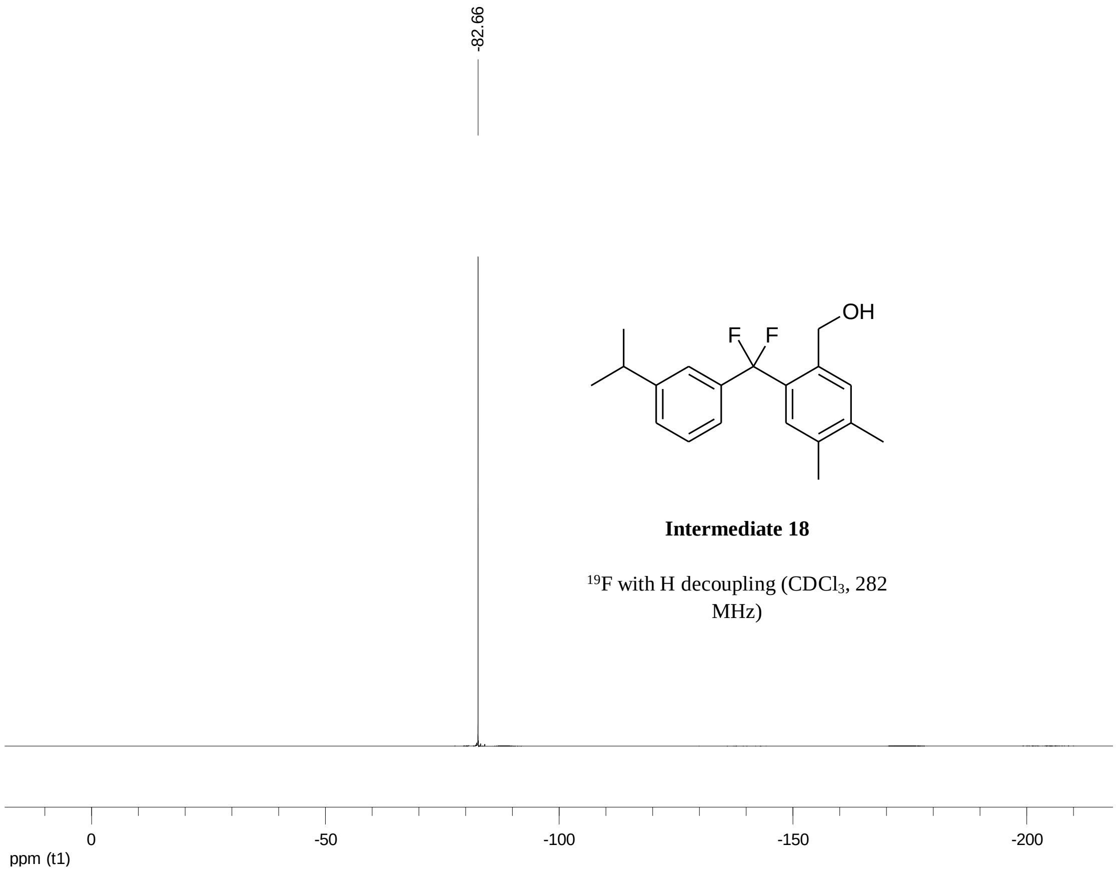
-82.66
F F
OH
Intermediate 18
19F with H decoupling (CDCl3, 282 MHz)
0
-50
-100
-150
-200
ppm (t1)

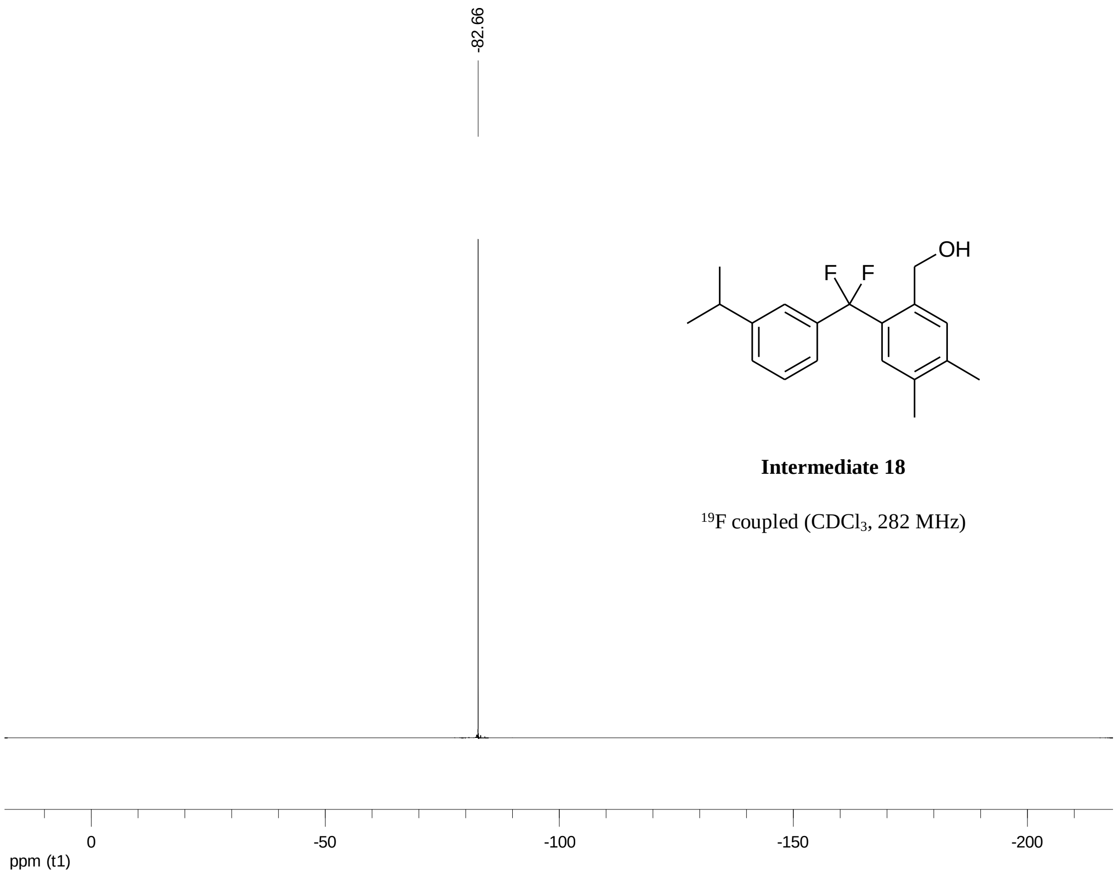
-82.66
F F
OH
Intermediate 18
19F coupled (CDCl3, 282 MHz)
0
-50
-100
-150
-200
ppm (t1)

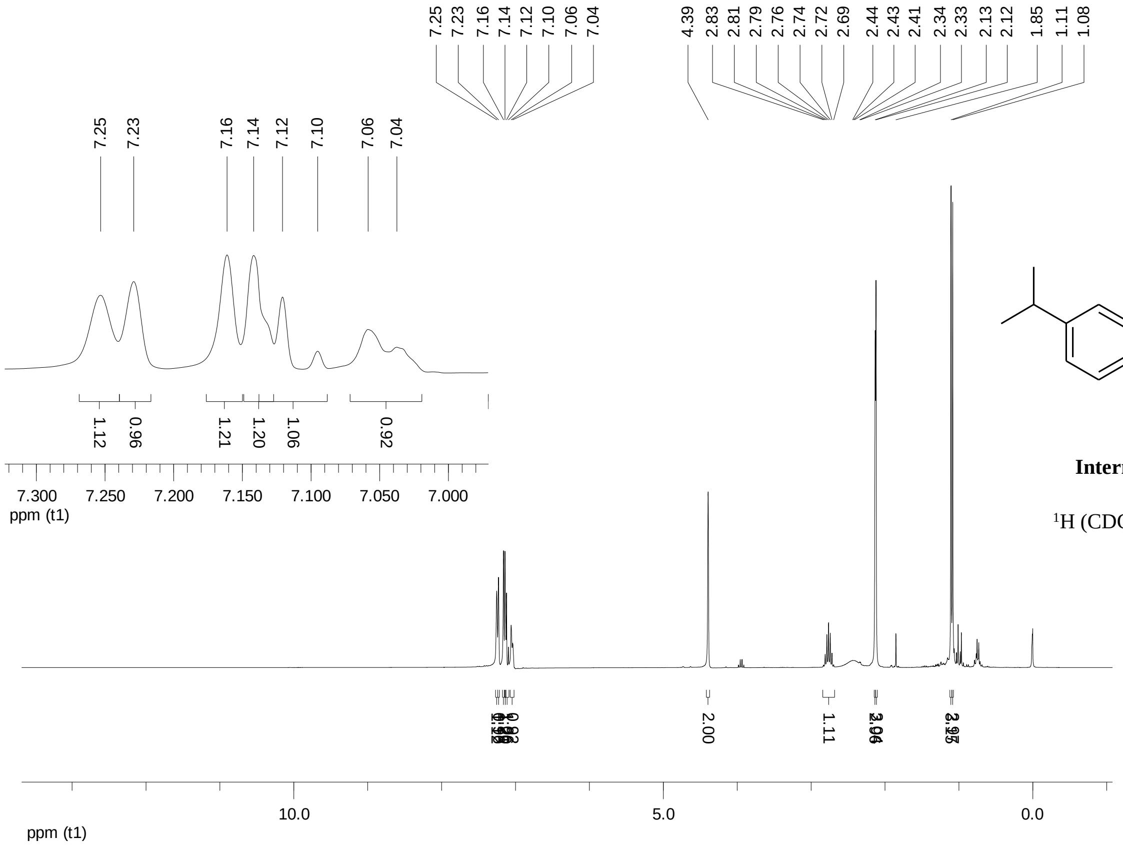

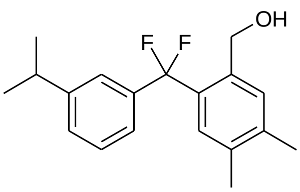

Intermediate 18

^{1}H ($CDCl_3$, 300 MHz)

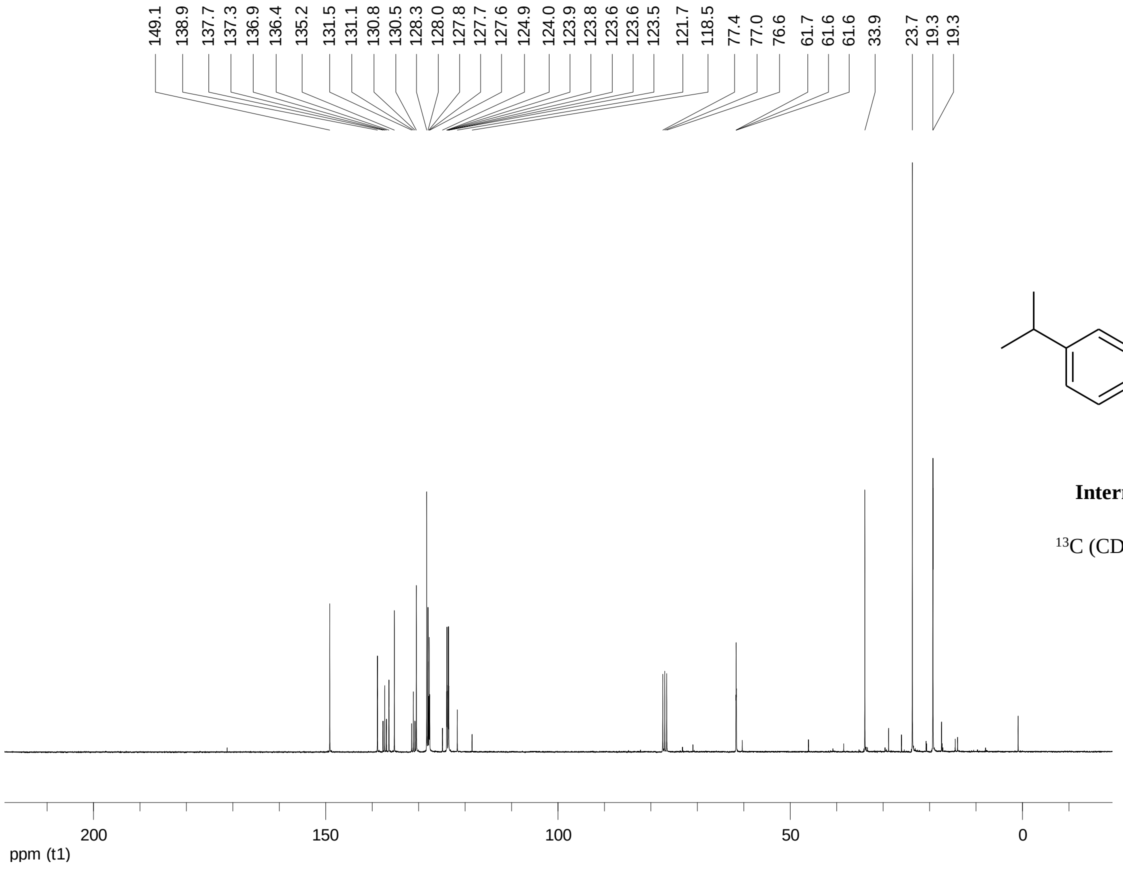

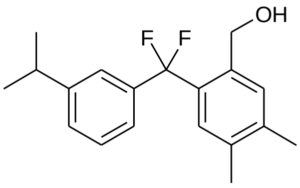

Intermediate 18

^{13}C ($CDCl_3$, 75 MHz)

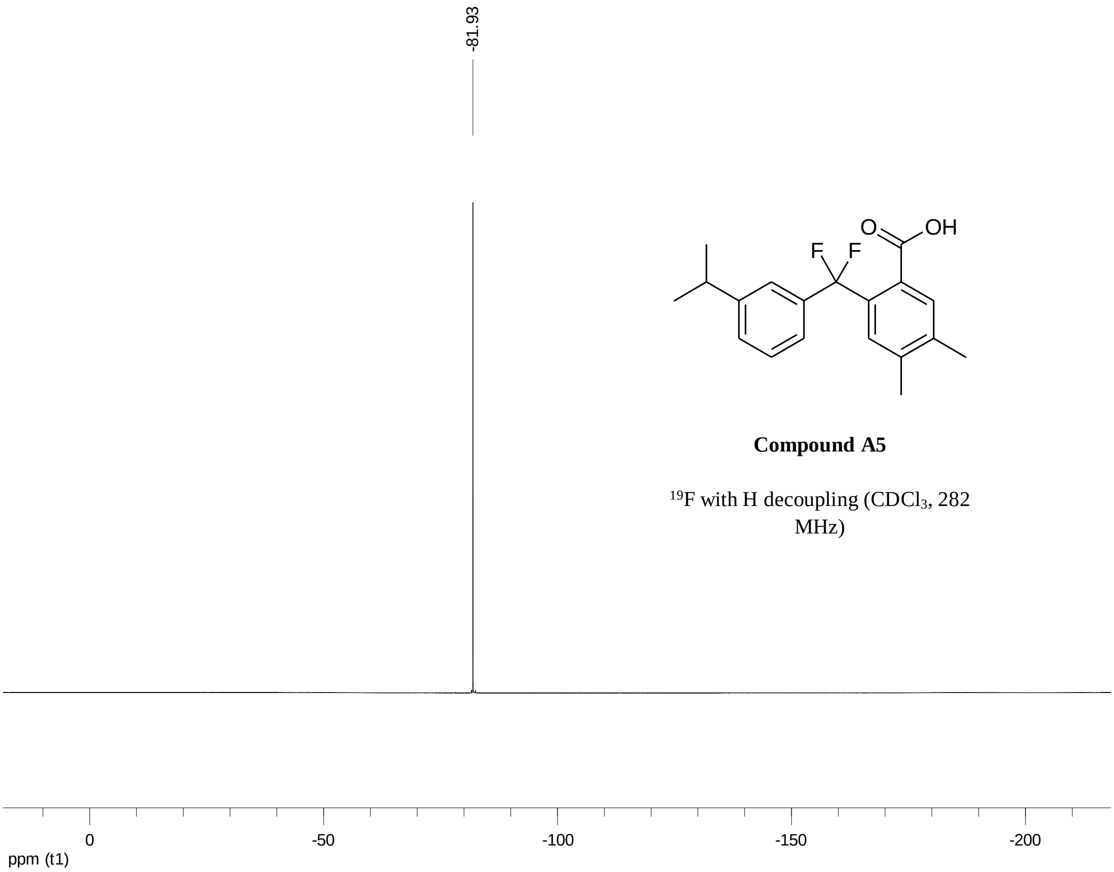

Compound A5

^{19}F with H decoupling ($CDCl_3$, 282 MHz)

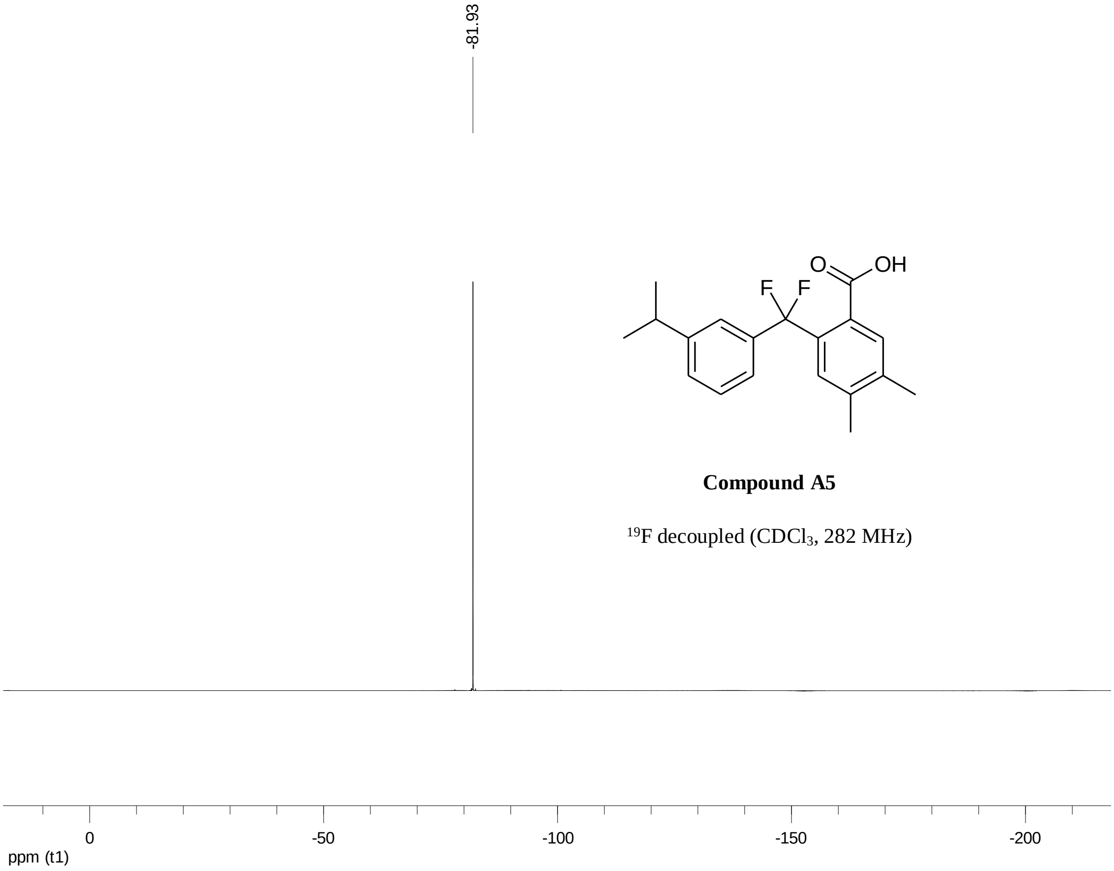

Compound A5

^{19}F decoupled ($CDCl_3$, 282 MHz)

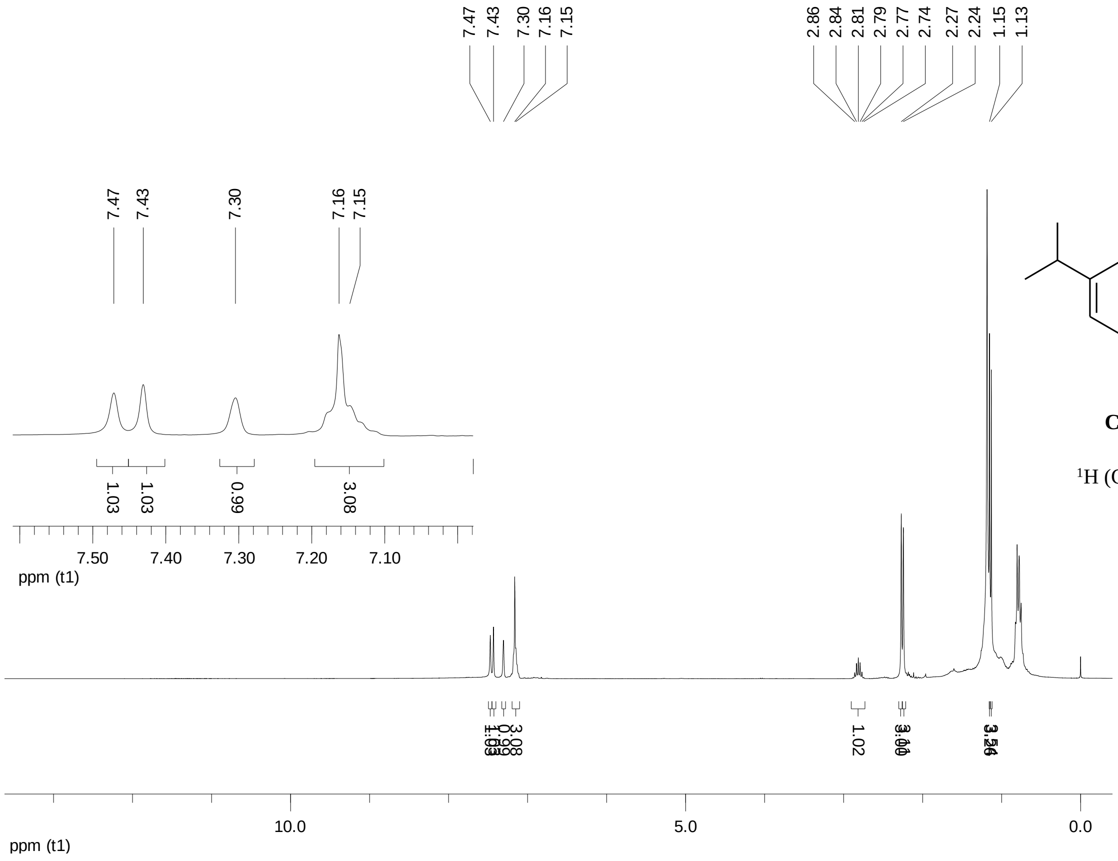

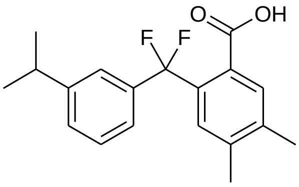

Compound A5

^{1}H (CDCl$_3$, 300 MHz)

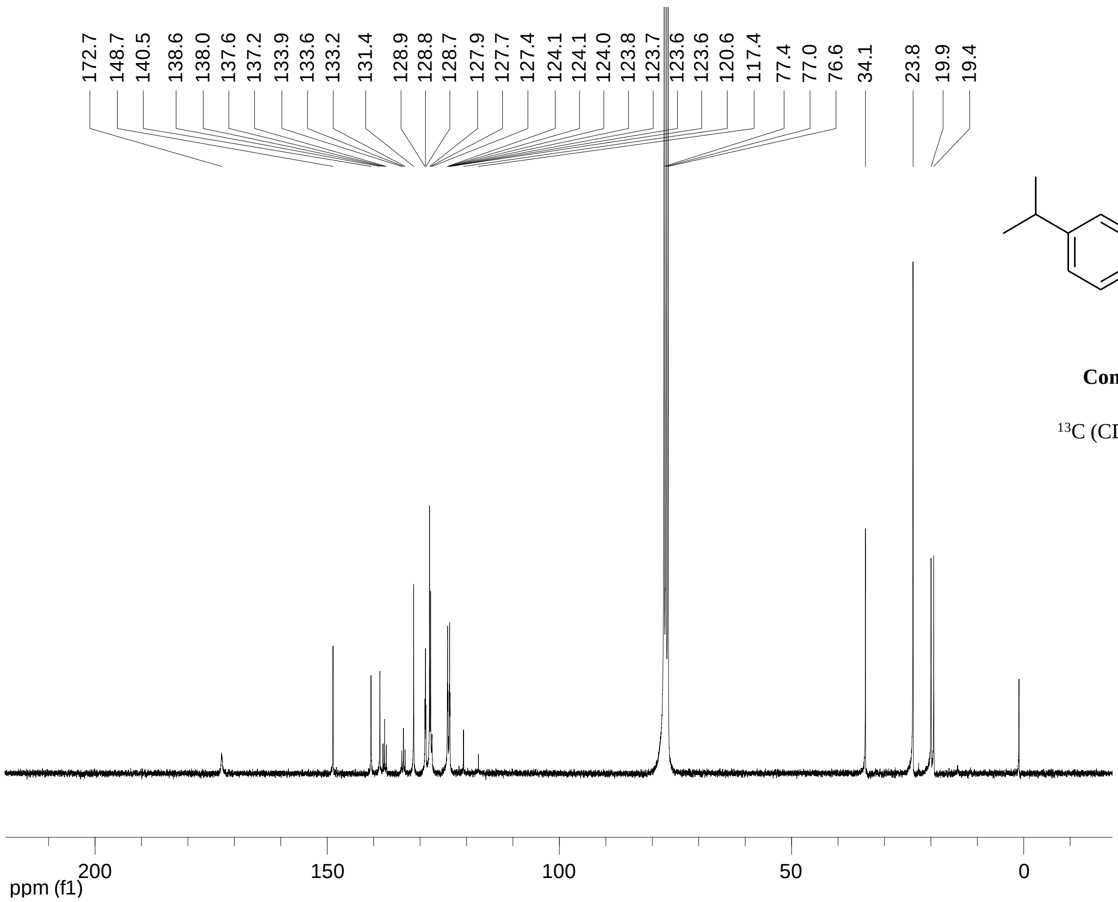

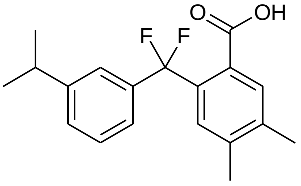

Compound A5

^{13}C (CDCl$_3$, 75 MHz)
